# Linked circadian outputs control elongation growth and flowering in response to photoperiod and temperature

**DOI:** 10.15252/msb.20145766

**Published:** 2015-01-19

**Authors:** Daniel D Seaton, Robert W Smith, Young Hun Song, Dana R MacGregor, Kelly Stewart, Gavin Steel, Julia Foreman, Steven Penfield, Takato Imaizumi, Andrew J Millar, Karen J Halliday

**Affiliations:** 1 SynthSys and School of Biological Sciences, University of EdinburghEdinburgh, UK; 2 Department of Biology, University of WashingtonSeattle, WA, USA; 3 Biosciences, University of ExeterExeter, UK

**Keywords:** gene regulatory networks, heat, hypocotyl elongation, photoperiodism, seasonal breeding

## Abstract

Clock-regulated pathways coordinate the response of many developmental processes to changes in photoperiod and temperature. We model two of the best-understood clock output pathways in *Arabidopsis*, which control key regulators of flowering and elongation growth. In flowering, the model predicted regulatory links from the clock to *CYCLING DOF FACTOR 1* (*CDF1*) and *FLAVIN-BINDING, KELCH REPEAT, F-BOX 1* (*FKF1*) transcription. Physical interaction data support these links, which create threefold feed-forward motifs from two clock components to the floral regulator *FT*. In hypocotyl growth, the model described clock-regulated transcription of *PHYTOCHROME-INTERACTING FACTOR 4* and *5* (*PIF4*, *PIF5*), interacting with post-translational regulation of PIF proteins by phytochrome B (phyB) and other light-activated pathways. The model predicted bimodal and end-of-day PIF activity profiles that are observed across hundreds of PIF-regulated target genes. In the response to temperature, warmth-enhanced PIF4 activity explained the observed hypocotyl growth dynamics but additional, temperature-dependent regulators were implicated in the flowering response. Integrating these two pathways with the clock model highlights the molecular mechanisms that coordinate plant development across changing conditions.

## Introduction

Plants are exposed to a wide range of light and temperature regimes that alter the molecular mechanisms controlling plant development. Seedling de-etiolation and floral transition represent critical stages in the plant life cycle that directly impact plant productivity. De-etiolation, which is characterised by embryonic leaf (cotyledon) greening and the cessation of embryonic stem (hypocotyl) elongation, is important for seedling establishment, whereas the time taken for the plant to reach the floral transition controls the balance between vegetative biomass and seed production. In the model plant *Arabidopsis thaliana,* long-day (LD) growth regimes lead to earlier flowering and shorter hypocotyls compared to short-day (SD) regimes (Corbesier *et al*, 1996; Kunihiro *et al*, 2011). Higher ambient temperatures promote early flowering and hypocotyl elongation (Mazzella *et al*, 2000; Halliday *et al*, 2002; Balasubramanian *et al*, 2006; Kumar *et al*, 2012).

The circadian clock is central to the photoperiodic response and provides 24-h timing information at the molecular level. While molecular clock components are not generally conserved across taxa, all circadian clocks include a gene circuit with interconnected negative feedback loops. In *Arabidopsis*, the circadian clock regulates up to 30% of genes at the transcript level, often intersecting with signalling pathways responsive to the external environment (Harmer *et al*, 2000; Harmer, 2009; Kinmonth-Schultz *et al*, 2013). This allows the clock to “gate” environmental responses to specific time windows within the daily cycle (Millar & Kay, 1996). In some cases, including photoperiodic regulation of flowering time and hypocotyl elongation, the gating circuit forms an “external coincidence” detector for time-specific environmental signals (Bünning, 1936; Roden *et al*, 2002; Yanovsky & Kay, 2002; Nozue *et al*, 2007).

**Figure 1:**
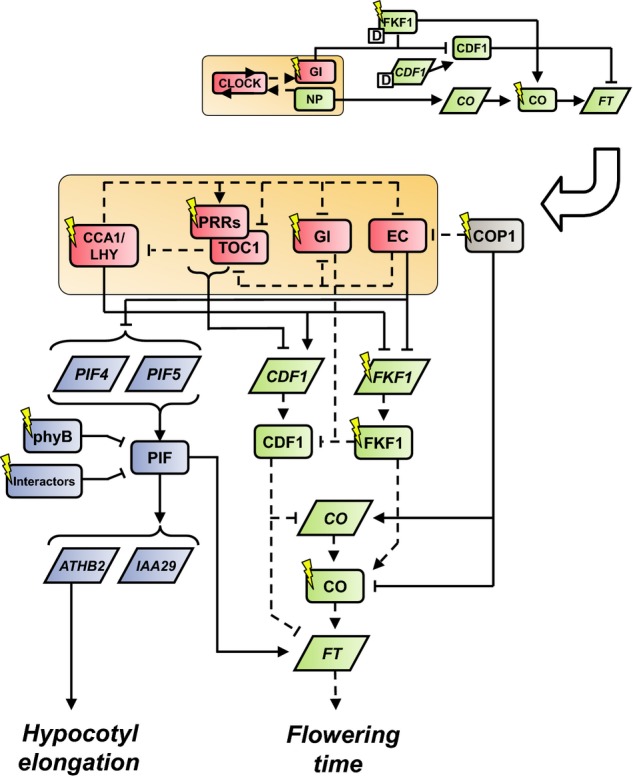
Regulatory connections present in model Our previous model of the photoperiod pathway (inset top; Song *et al*, 2012) included the circadian clock model of Locke *et al* (2005) (orange, inset top). The new model includes the circadian clock model of Pokhilko *et al* (2012) (orange, inset bottom). Circadian clock components are represented in red, flowering time pathway components in green and hypocotyl elongation pathway components in blue. Dashed lines represent forms of regulation that have been modelled previously; solid lines represent new regulatory connections. Protein components are represented by rectangles and mRNA by parallelograms. Model components are denoted by their abbreviated names (see text). PIF-interacting proteins (e.g. DELLAs, PAR1, HFR1) are designated “Interactors” in the hypocotyl elongation pathway.

In *Arabidopsis*, mathematical modelling has been an important tool for elucidating the architecture of the clock circuit, which can be viewed as an elaborated repressilator (Fig1, bottom inset; Pokhilko *et al*, 2012). Transcript levels of the key morning genes *CIRCADIAN CLOCK ASSOCIATED 1* (*CCA1*) and *LATE ELONGATED HYPOCOTYL* (*LHY*) peak at dawn (Wang & Tobin, 1998). The CCA1 and LHY proteins inhibit transcription of EVENING COMPLEX (EC) components, EARLY FLOWERING 4 (ELF4), ELF3 and LUX ARRYTHMO (LUX), delaying their accumulation until dusk (Doyle *et al*, 2002; Hazen *et al*, 2005; Dixon *et al*, 2011; Nusinow *et al*, 2011; Lu *et al*, 2012). The EC in turn inhibits transcription of *PSEUDO-RESPONSE REGULATOR 9* (*PRR9*) and *TIMING OF CAB EXPRESSION 1* (*TOC1/PRR1*) at night (Helfer *et al*, 2011; Herrero *et al*, 2012; Pokhilko *et al*, 2012). The family of PRR proteins, including PRR9, PRR7, PRR5 and TOC1, repress transcription of *CCA1* and *LHY* through the day and early night, completing the CCA1/LHY-EC-PRR repressilator (Huang *et al*, 2012; Nakamichi *et al*, 2012). In addition to this central loop, GI protein suppresses EC formation and TOC1 accumulation through interactions with ELF3 and ZEITLUPE (ZTL) proteins, respectively (Kim *et al*, 2007; Yu *et al*, 2008; Pokhilko *et al*, 2012).

In the flowering pathway, GI controls expression of floral activators *CONSTANS* (*CO*) and *FLOWERING LOCUS T* (*FT*) by forming a blue light-dependent complex with FLAVIN-BINDING, KELCH REPEAT, F-BOX 1 (FKF1) (Imaizumi *et al*, 2003, 2005; Sawa *et al*, 2007; Sawa & Kay, 2011). The GI-FKF1 complex degrades CYCLING DOF FACTOR 1 (CDF1) protein that represses *CO* and *FT* transcription (Imaizumi *et al*, 2005; Fornara *et al*, 2009; Song *et al*, 2012). Under LDs, activation of *FLOWERING LOCUS T (FT)* expression is principally controlled by CO protein levels, which are stabilised by FKF1 at the end of the long photoperiod (Suárez-López *et al*, 2001; Song *et al*, 2012). Furthermore, CO protein is regulated by light such that CO levels are low in red light, while blue and far-red light stabilise CO (Valverde *et al*, 2004). This control reinforces the accumulation of CO protein levels in the evening of LDs, leading to an increase in the floral signal. Conceptually, these molecular interactions result in a double external coincidence mechanism involving multiple clock outputs, but the combined effects of this rhythmic mechanism have not been tested quantitatively or incorporated into previous mathematical models (Song *et al*, 2012).

Similarly, photoperiodic elongation growth is controlled by clock- and light-regulated processes (Nozue *et al*, 2007; Niwa *et al*, 2009; Kunihiro *et al*, 2011). The circadian clock regulates the transcription of morning-expressed genes *PHYTOCHROME-INTERACTING FACTORS 4* and *5* (*PIF4, PIF5*) through repression by the EC (Nozue *et al*, 2007; Nusinow *et al*, 2011). The resulting PIF proteins control gene expression by forming homo- and hetero-dimers that bind to G- and E-box motifs in targeted promoters (Hornitschek *et al*, 2009, 2012; Zhang *et al*, 2013). During the day, PIF activity is thought to be compromised due to interactions with phytochrome B (phyB), the key red light photoreceptor, resulting in rapid PIF phosphorylation and degradation (Park *et al*, 2004, 2012; Al-Sady *et al*, 2006; Nozue *et al*, 2007; Jang *et al*, 2010). This is proposed to restrict PIF activity to the end of the night in SDs, coinciding with the time of maximal hypocotyl growth rate (Nozue *et al*, 2007, 2011; Michael *et al*, 2008a), a hypothesis that we re-examine here. Several other light-regulated proteins also repress PIF signalling, including DELLAs, PHY RAPIDLY REGULATED 1 (PAR1), LONG HYPOCOTYL IN FAR-RED 1 (HFR1) and ELONGATED HYPOCOTYL 5 (HY5) (de Lucas *et al*, 2008; Foreman *et al*, 2011; Hao *et al*, 2012; Chen *et al*, 2013). Among the known targets of PIF4 and PIF5 are *INDOLE-3-ACETIC ACID INDUCIBLE 29* (*IAA29*) and *ARABIDOPSIS THALIANA HOMEOBOX 2* (*ATHB2*) (Kunihiro *et al*, 2011), genes which are involved in auxin signalling. Thus, PIF4 and PIF5 appear to regulate hypocotyl elongation through auxin signalling (Kunihiro *et al*, 2011; Nozue *et al*, 2011; Hornitschek *et al*, 2012). While a number of the key molecular interactions in this pathway have been experimentally characterised, a model of the dynamic regulation of PIF activity in light:dark (L:D) cycles has not previously been developed.

PIF4 and, to a lesser extent, PIF5 promote hypocotyl elongation in response to warm ambient temperatures (27°C; Koini *et al*, 2009; Stavang *et al*, 2009). Increased temperature leads to higher PIF4 transcript and protein levels and longer hypocotyls (Koini *et al*, 2009; Stavang *et al*, 2009; Foreman *et al*, 2011; Nomoto *et al*, 2012a; Yamashino *et al*, 2013; Mizuno *et al*, 2014). Recently, PIF4 has also been implicated in the warm temperature-induced acceleration of flowering in SDs by binding to the *FT* promoter independently of the CO-*FT* photoperiodic pathway (Kumar *et al*, 2012). Other temperature-sensitive regulators of *FT* have recently been identified that are not thought to be part of the photoperiodic response (Lee *et al*, 2013; Posé *et al*, 2013). Once again, while a number of regulators have been identified in this pathway, their combined effects have not previously been described in a mathematical model.

In this study, we have constructed a mathematical model to integrate and reconcile the complex molecular mechanisms in the photoperiodic pathways of flowering and hypocotyl elongation in *Arabidopsis*. In the flowering pathway, we extended our previous model, which was built to determine how FKF1 protein regulates levels of *FT* mRNA through CDF1 and CO protein interactions (Song *et al*, 2012). The updated model was able to match *CO* and *FT* mRNA rhythmic expression data in different photoperiods and in mutants of the flowering pathway (e.g. *fkf1*, *gi*, *cdf1*, *CO-ox*, *CO-ox;fkf1*, *CO-ox;CDF1-ox*). However, this model required FKF1 protein and *CDF1* mRNA timeseries data to be input into the model, meaning that simulation of multiple photoperiods and mutants would require the generation of multiple input data sets (Fig1, top inset; Song *et al*, 2012). To improve this aspect of the model, we wished to incorporate circadian regulation of *CDF1* and *FKF1* mRNA, removing data inputs to the model (Fig1, bottom inset). This modification improved the predictive power of the model and allowed us to investigate how changes in clock dynamics affect components of the flowering pathway in clock mutants and different photoperiods. By postulating and experimentally validating circadian regulators of *CDF1* and *FKF1* transcription, the model recapitulates the acceleration of flowering in LDs.

In the hypocotyl elongation pathway, we demonstrate that known transcriptional and post-transcriptional regulation of PIF explain phenotypes and PIF target transcript dynamics under a variety of environmental and genetic manipulations. We then use microarray data to identify other transcripts that have similar dynamics and that are therefore likely to be under the control of PIFs in light:dark cycles. Finally, we explore crosstalk between the flowering and hypocotyl pathways by simulating PIF regulation of *FT* mRNA, in order to test the hypothesis that temperature regulates flowering independently of CO. The results highlight the complexity of the network structure underlying circadian-, light- and temperature-regulated processes.

## Results

### Refining the photoperiodic flowering model

**Figure 2:**
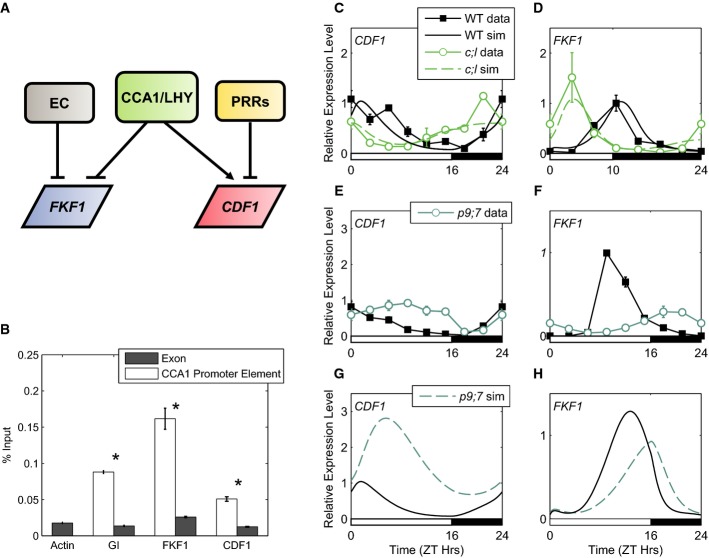
Modelling the circadian regulation of *CDF1* and *FKF1* mRNA

We determined potential mechanisms by which the circadian clock might regulate *FKF1* and *CDF1* mRNA by inspection of published data sets (Mizoguchi *et al*, 2005; Niwa *et al*, 2007; Ito *et al*, 2008; Edwards *et al*, 2010). From these, we observed that *FKF1* mRNA peaks at a similar phase to *GI* transcription across multiple photoperiods, while both respond in a similar manner to perturbations of the circadian clock. Under 10L:14D and 16L:8D cycles, the peak of *FKF1* expression at ZT9–10 matches that of *GI* (ZT = zeitgeber time, where dawn in an L:D cycle is at ZT0; note: throughout, we will refer to 8L:16D and 10L:14D as short-day (SD) conditions, and 16L:8D as long-day (LD) conditions). Both *FKF1* and *GI* expression have an earlier peak phase in *cca1;lhy* mutants, while they exhibit only minor phase changes in *prr* mutants (Imaizumi *et al*, 2003; Niwa *et al*, 2007; Ito *et al*, 2008). Furthermore, *FKF1* transcription, like that of *GI*, is acutely stimulated by red light (Tepperman *et al*, 2004; Locke *et al*, 2005). Based on this evidence, we modelled *FKF1* transcription similar to *GI*: to be inhibited by CCA1/LHY proteins and the EC, and acutely activated by light (see Computational Methods in Supplementary Information; Figs1 and 2A). This model is additionally supported by recent work highlighting similarities between the promoter sequences of *FKF1* and *GI* (Berns *et al*, 2014). In deciphering how *CDF1* transcription is regulated by the clock, we noted that previous reports have shown that *CDF1* mRNA levels are strongly regulated by the transcription-repressing PRR protein family (Nakamichi *et al*, 2007, 2012; Niwa *et al*, 2007; Ito *et al*, 2008; Huang *et al*, 2012). Mutations of the *PRRs* (e.g. the *prr9;7* double mutant in Fig2E) lead to elevated daytime expression of *CDF1* (Nakamichi *et al*, 2007; Ito *et al*, 2008). However, if *CDF1* mRNA was solely regulated by the PRRs, we would predict an increase in *CDF1* expression at dawn in *cca1;lhy* double mutants, since PRR levels are low in this mutant (Dixon *et al*, 2011). Instead, the *cca1;lhy* double mutant has an advanced phase of *CDF1* expression, with decreased expression at dawn in both SDs and LDs (Fig2C, Supplementary Figs S1A and S2A; Nakamichi *et al*, 2007; Niwa *et al*, 2007). The simplest explanation for this difference between predicted and observed rhythms of *CDF1* mRNA in *cca1;lhy* is that CCA1/LHY proteins play a role in activating *CDF1* expression alongside repression by the PRR proteins. By incorporating both regulatory features, the model qualitatively matched the peak of *CDF1* mRNA expression at dawn in the WT. The model can also describe *CDF1* transcript profiles in the *prr9;7* and *cca1;lhy* double mutants (Fig2E and G; Supplementary Figs S1D and S2D), indicating that this combination of regulatory mechanisms is sufficient to explain the observed transcript profiles.

Schematic of proposed circadian regulators of *FKF1* and *CDF1* transcription.

Experimental validation for CCA1 regulation of the flowering pathway. ChIP data showing CCA1 enrichment in regions containing CCA1 elements (CBS or EE; white bars) in *GI*, *FKF1* and *CDF1* promoters, compared to regions of their respective exons (dark bars). Locations of primers for *GI* (GI-a, GI-N), *FKF1* (FKF1-a, FKF1-N) and *CDF1* (CDF1-a, CDF1-N) are shown in Supplementary Fig S3. Seedlings were grown for 14 days 12L:12D cycles at 22°C and harvested at ZT2. Statistical analysis performed using Welch tests, *P < 0.005. Error bars represent standard error of technical replicates.

*CDF1* mRNA in LDs (C) and *FKF1* mRNA in SDs (D), from WT (data: black lines, filled squares; simulation: black lines), and the *cca1;lhy* mutant (data: green lines, open circles; simulation: dashed green lines) (data sets used for parameter optimisation).

*CDF1* (E) and *FKF1* (F) mRNA data in LDs, from WT (as in C, D) and the *prr9;7* mutant (blue-green line, open circles).

*CDF1* (G) and *FKF1* (H) mRNA simulations in LDs, from WT (as in C, D) and the *prr9;7* mutant (dashed blue-green line). Data information: Data in (C, E, F) from Nakamichi *et al* (2007). Data in (D) from Niwa *et al* (2007). Error bars in (C–F) represent standard deviation.

Our model proposes that the morning component CCA1/LHY regulates both *CDF1* and *FKF1* transcription. These new hypotheses were tested experimentally with chromatin immunoprecipitation (ChIP) experiments using *pCCA1:CCA1-HA-YFP* (Fig2B and Supplementary Fig S3; Yakir *et al*, 2009). Promoter sequences containing the CCA1-binding sites (CBS = AAAATCT; Wang *et al*, 1997) and evening elements (EE = AATATCT; Michael & McClung, 2002) within 3 kbp of the transcription start sites of *CDF1* and *FKF1* were enriched in the *pCCA1:CCA1-HA-YFP* ChIP (Fig2B and Supplementary Fig S3; Supplementary Dataset S11). These data, therefore, suggest that *CDF1* and *FKF1* are directly regulated by CCA1.

To further validate the models of *CDF1* and *FKF1* mRNA regulation, we compared simulations of *CDF1* and *FKF1* transcription to data sets that were not used for model optimisation (Fig2E–H). Figure2G shows that the mean level of simulated *CDF1* mRNA is increased in *prr9;7* double mutants in LDs, while *FKF1* mRNA has a lower amplitude and delayed phase (Fig2H), both of which qualitatively match the data (Figs2E and F). *CDF1* mis-regulation ends earlier in the night in the data but persists to the late night in the model, suggesting that additional modes of *CDF1* regulation may exist. As the models were constructed and parameterised using data from WT and *cca1;lhy* lines (see Computational Methods in Supplementary Information; Fig2C and D), the qualitative match to the *prr9;7* double mutant validates our simple assumptions for circadian regulation of *CDF1* and *FKF1* mRNA. Thus, the model captures the main features of regulation of *CDF1* and *FKF1* by the circadian clock, and their model-predicted regulation by CCA1/LHY was experimentally supported. We next examined the downstream regulation of the CDF1 and FKF1 target genes *CO* and *FT*.

### Model of transcriptional regulation of *CO* and *FT* mRNA suggests novel roles for circadian clock components

**Figure 3:**
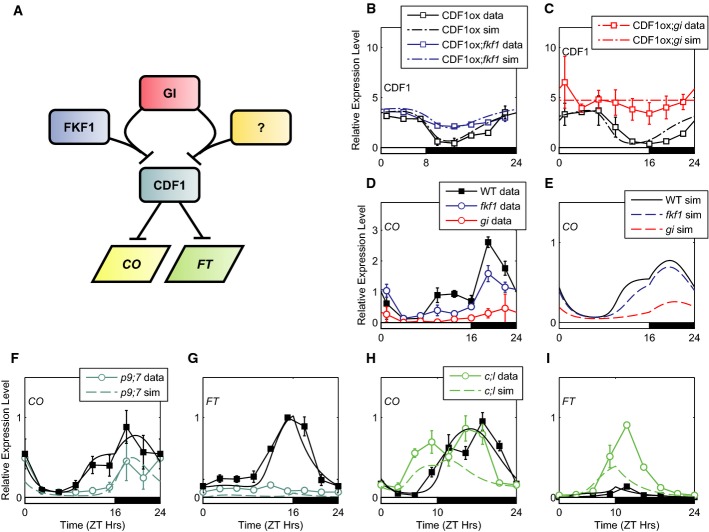
Modelling the regulation of *CO* and *FT* mRNA

Previous studies have highlighted that CDF1 protein turnover is regulated by the blue light-dependent GI-FKF1 protein complex (Imaizumi *et al*, 2005; Sawa *et al*, 2007). Consistent with this notion, when the *fkf1* mutation is introduced into lines expressing *CDF1* transcript, either constitutively or under the control of the *CDF1* promoter, CDF1 protein is degraded at a slower rate than WT, resulting in rhythmic CDF1 with reduced amplitude and higher mean levels (Fig3B; Supplementary Fig S4A; Imaizumi *et al*, 2005). However, CDF1 protein levels are still rhythmic in the *CDF1-ox;fkf1* plants, suggesting that CDF1 turnover is also regulated by the circadian clock independently of FKF1. As GI acts in protein complexes with other members of the FKF1 protein family, notably ZTL in the circadian clock system (Kim *et al*, 2007), we wanted to determine how the absence of GI function altered CDF1 levels. To study the effect of the *gi* mutation on the post-transcriptional regulation of CDF1 protein, we used a constitutive *CDF1* overexpressor line that carries the *gi-2* mutation (35S:HA-CDF1;*gi-2*). *CDF1-ox* and *CDF1*-ox;*gi-2* plants were grown for 13 days in 16L:8D cycles and harvested at 3-h intervals, and CDF1 protein levels were measured by immunoblotting. Our data show that the *gi-2* mutant had high mean levels of CDF1 protein, similar to *fkf1* mutants, but in *gi,* the level did not vary significantly among time points (Fig3C; Supplementary Dataset S2; Imaizumi *et al*, 2005; Sawa *et al*, 2007). However, in the case of the *fkf1* mutant, a residual shallow rhythm in CDF1 protein levels was observed (Fig 3B), suggesting that GI may play an additional role in regulating CDF1. As CDF1 is a key negative regulator of *CO* mRNA, high mean level of CDF1 protein in the *gi* and *fkf1* mutants leads to low expression levels of *CO* mRNA in these mutants (Fig3D; Suárez-López *et al*, 2001; Sawa *et al*, 2007; Fornara *et al*, 2009). However, *CO* transcript is lower in the *gi* mutant than in the *fkf1* mutant (Fig3D), providing further support for an FKF1-independent role for GI in the regulation of CDF1 protein. The inclusion of FKF1-dependent and FKF1-independent effects of GI on CDF1 protein stability in the model are sufficient to explain the lower *CO* transcript levels observed in the *gi* mutant, as compared to the *fkf1* mutant (Fig3D), and the low *FT* levels were seen in LDs in both mutants (Supplementary Figs S5 and S6).

Schematic of the role of GI in destabilising CDF1 through an FKF1-dependent and FKF1-independent mechanisms, with resulting effects on *CO* and *FT* mRNA abundance.

CDF1 protein data and simulations in a 35S:3HA-CDF1 line (CDF1ox; black lines, open squares) and 35S:HA-CDF1;*fkf1* mutant (CDF1ox;*fkf1*; blue lines, open squares) in LDs. Data from Imaizumi *et al* (2005).

CDF1 protein data and simulations in a 35S:HA-CDF1 line (CDF1ox; as in B) and 35S:HA-CDF1;gi-2 mutant (CDF1ox;*gi*; red lines, open squares) in LDs. Plants were grown for 10 days in 16L:8D cycles.

*CO* data and simulations in 16L:8D in WT (data: black lines, filled squares; simulation: black lines), the *gi-2* mutant (data: red lines, open circles; simulation: dashed red lines) and *fkf1* mutants (data: blue lines, open circles; simulation: dashed blue lines). Data from Sawa *et al* (2007).

*CO* and *FT* mRNA data and simulations in LDs in WT (as in D, E) and the *prr9;7* mutant (data: blue-green lines, open circles; simulation: dashed blue-green lines). Data from Nakamichi *et al* (2007).

*CO* and *FT* mRNA data and simulations in 10L:14D in WT (as in D) and the cca1;lhy mutant (data: green lines, open circles; simulation: dashed green lines). Data from Nakamichi *et al* (2007). Data information: Error bars in (B–D) represent standard error. Error bars in (F–I) represent standard deviation.

Having connected the clock model to a model of *CO/FT* regulation, we then compared model simulations and data for *CO* and *FT* mRNA from plants with mutations in clock genes. In the case of the *prr9;prr7* and *CCA1-ox* mutants, model simulations matched data showing reduced levels of *CO* and *FT* mRNA throughout the day (Fig3F and G; Supplementary Fig S7; data not used for parameter optimisation). In both cases, the simulated high level of *CDF1* mRNA and low level of *FKF1* mRNA (e.g. Fig2E–H for the case of the *prr9;prr7* mutant) result in low levels of *CO* and *FT* mRNA throughout the day, matching experimental data (Fig3F and G; Supplementary Fig S7). In the case of the *elf3* mutant, model simulations matched the increase in *FT* expression in both SDs and LDs despite overestimating the increase in *CO* mRNA during the day (Lu *et al*, 2012; Supplementary Fig S7; data not used for parameter optimisation). In this case, this is explained by the simulated low level of *CDF1* mRNA and high level of *FKF1* mRNA in this mutant.

In contrast to the above cases, the model is unable to fully describe the dynamics of *CO* and *FT* mRNA in the *cca1;lhy* double mutant [measured in the same experiments (Nakamichi *et al*, 2007)]. Simulations of *CDF1* and *FKF1* mRNA match the data for the double mutant, as described above. However, the predicted transcript profiles of *CO* and *FT* depart qualitatively from the data at ZT12-16 (Fig3H and I; Supplementary Figs S1 andS2). The simulations correctly show a 3- to 6-h advanced phase of *CO* and *FT* expression, and the increase in *FT* levels with respect to the WT is sufficient to explain the early-flowering phenotype of the *cca1;lhy* mutant in SDs. However, the model underestimates the peak levels of *CO* and *FT* mRNA observed at ZT12-16, especially in LDs (Fig3H and I, Supplementary Figs S1 and S2). Thus, CCA1 and LHY may also regulate *CO* and *FT* transcription by another mechanism in parallel to or downstream of *CDF1* mRNA (see Model Behaviour in Supplementary Information). This regulation might be direct or might result from a phase shift in the expression of other clock-regulated components.

### Description of flowering mutants is maintained with new connections to the circadian clock

Our previous flowering time model was able to qualitatively describe several mutants specific to the flowering pathway. With the new circadian regulation of *CDF1* and *FKF1* mRNA and CDF1 protein, our extended model also matches *FT* mRNA in *fkf1* mutants as well as in *CO-ox;fkf1* and *CO-ox;CDF1-ox* lines (Supplementary Fig S8A). Furthermore, the model retains the important feature of the previous model showing that the FKF1 protein has a larger effect on *FT* mRNA through its regulation of CO protein than through degradation of CDF1 protein (Supplementary Fig S8B; Song *et al*, 2012). Thus, the present model is consistent with past results as well as additional molecular and genetic data, providing a suitable basis for further extension. In particular, we extended the model to consider the combined circadian and light regulation of PIF4 and PIF5 activity, allowing us to investigate the regulation of rhythmic growth by PIF4 and PIF5 and to understand crosstalk between PIF4, PIF5 and CO in the regulation of *FT*.

### Modelling the circadian regulation of *PIF4* and *PIF5* mRNA

**Figure 4:**
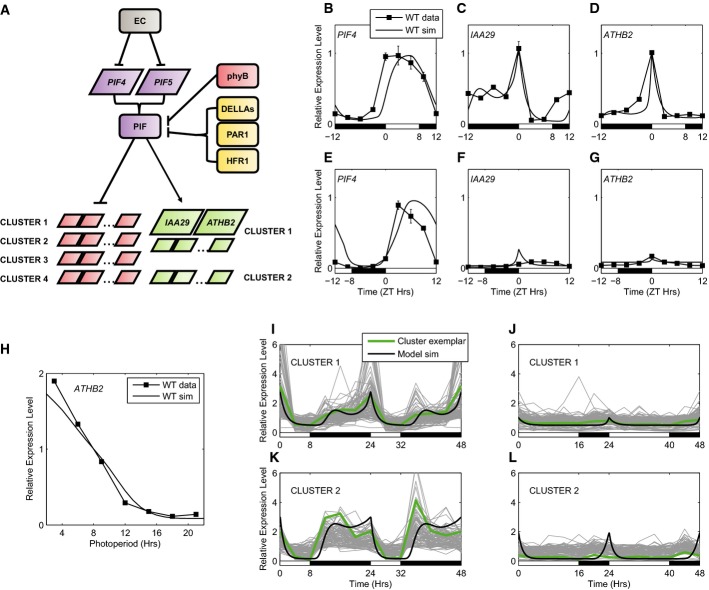
Photoperiodic regulation of PIF activity

Hypocotyl elongation, like flowering time, is subject to photoperiodic regulation. In contrast to the FKF1-CO-FT pathway, which is active in LDs, PIF4-induced and PIF5-induced hypocotyl extension is observed in SDs (Niwa *et al*, 2009; Kunihiro *et al*, 2011). Here, we describe the development of a model describing the photoperiodic induction of hypocotyl elongation through PIF4 and PIF5. As a first step, we constructed a model of *PIF4* and *PIF5* transcription, which is known to be controlled by the circadian clock (Yamashino *et al*, 2003). This regulation has been shown to involve direct inhibition of transcription by the EC (Fig4A; Nozue *et al*, 2007; Nusinow *et al*, 2011). In order to test whether this regulation is sufficient to explain observed patterns of *PIF4* and *PIF5* expression, we started by constructing a model in which the EC is the sole regulator of *PIF4* and *PIF5* transcription.

Schematic of transcriptional and post-transcriptional regulation of PIF activity.

*PIF4*, *IAA29* and *ATHB2* mRNA levels in SDs in WT (data: black lines, filled squares; simulation: black lines).

As in (B–D), for LDs. Data from Nomoto *et al* (2012b).

*ATHB2* mRNA at dawn (ZT0) across a range of photoperiods. Data from Kunihiro *et al* (2011).

Comparison of model simulations with microarray time course data from the two largest clusters of PIF-induced targets (Cluster 1: 107 genes, Cluster 2: 84 genes), in SDs (I, K) and LDs (J, L) [data from Michael *et al* (2008b)]. Data information: Error bars represent standard deviation.

This model of *PIF4* and *PIF5* regulation captures important properties of mRNA profiles from WT, *elf3* and *prr9;7;5* backgrounds (Fig4B and E; Supplementary Figs S9A and D and S10A and D). In an *elf3* background, the level of *PIF4* transcripts is increased throughout the night (Nomoto *et al*, 2012b); this behaviour is matched by simulations (Supplementary Fig S9A and D). Similar behaviour is seen in the case of the *prr9;7;5* mutant in data and simulations (Supplementary Fig S10A and D; Nomoto *et al*, 2012b). Additionally, it should be noted that model simulations do not reproduce the steady increase in *PIF4* expression observed during the night in both the *elf3* and *prr9;7;5* mutants (Supplementary Figs S9A and B and S10A and B). Rather, model simulations in both cases predict a constant high level of *PIF4* transcript. Residual rhythms of *PIF4* and *PIF5* in the *elf3* mutant suggested a role for additional circadian regulators of *PIF4* and *PIF5* transcription. In particular, a small daytime peak in expression of *PIF4* and *PIF5* in the *elf3* mutant has been observed in multiple experiments (Lu *et al*, 2012; Nomoto *et al*, 2012b), and EE motifs are present in the *PIF4* and *PIF5* promoters. This suggested a possible role for CCA1 and LHY in activating *PIF4* and *PIF5* expression. However, our CCA1 ChIP experiments did not detect binding of CCA1 to the *PIF4* and *PIF5* promoters (Supplementary Fig S3). We also note that the observed dynamics of *PIF4* and *PIF5* mRNA required the simulated activity of the EC to be advanced by ∽2 h, providing a closer match to previously published data sets of EC dynamics (see Computational Methods and Model Behaviour in Supplementary Information; Nusinow *et al*, 2011). The need for further information on the EC was previously highlighted during the development of the circadian clock model (Pokhilko *et al*, 2012).

### Complex dynamics of PIF proteins predict bimodal control of target genes

Our next aim was to extend the model to incorporate post-transcriptional PIF regulation and the PIF target genes *ATHB2* and *IAA29*, whose expression correlates with hypocotyl elongation (Kunihiro *et al*, 2011; Nomoto *et al*, 2012a,b). We therefore introduced the regulation of PIF protein levels by active phyB, allowing us to simulate PIF degradation during the day (Supplementary Fig S11). In addition, we modelled inhibition of PIF activity by an “Interactor” class of proteins, representing PIF-binding proteins that are stimulated by light and that have been shown to inhibit PIF activity. This large and varied group includes DELLAs, PAR1, HFR1, HY5, PIL1 and phyB (Fig4A; Osterlund *et al*, 2000; Achard *et al*, 2007; de Lucas *et al*, 2008; Hornitschek *et al*, 2009; Foreman *et al*, 2011; Hao *et al*, 2012; Bai *et al*, 2012; Oh *et al*, 2012; Chen *et al*, 2013; Luo *et al*, 2014). Active PIF proteins promote the expression of *ATHB2* and *IAA29*, while the “Interactor” proteins inhibit PIF activity for these targets. Due to increased synthesis of the “Interactor” proteins during the light period, the inhibition of PIF proteins acts in tandem with phyB-dependent degradation to suppress PIF activity during the day (Supplementary Fig S11A–C).

Combining the regulation of PIF protein activity by light with circadian regulation of *PIF4* and *PIF5* transcription was sufficient to describe the observed photoperiod response of *ATHB2* and *IAA29* mRNA accumulation (Fig4C, D and F–H). In SDs, a high peak in expression at dawn is observed in both targets in both data and simulations. Additionally, the model matched the increase in *IAA29* transcript levels throughout the night in SDs (Fig4C). In the model, this behaviour is the result of high *PIF4* and *PIF5* transcript levels coinciding with darkness at ZT8-12 in SDs, resulting in an SD-specific increase in PIF activity at this time and a peak in target gene expression at ZT14. Analysis of model dynamics under random parameter perturbations confirmed that this behaviour is observed across a broad range of parameter values (Supplementary Fig S12; see Model Behaviour in Supplementary Information). The physiological significance of this secondary peak is suggested by the observation that 8 out of 11 putative PIF target genes inspected in Nomoto *et al* (2012b) displayed this post-dusk increase in a SD-specific manner and that a similar secondary peak in the rate of hypocotyl elongation has been observed in SDs (Nozue *et al*, 2007, 2011).

Recent experiments with dark-grown seedlings have identified an expansive transcriptional network downstream of the PIFs (Zhang *et al*, 2013), with 699 genes identified by RNA-Seq as having decreased transcript levels in *pif1;4;5* mutants, while 755 genes were identified as having increased levels in *pif1;4;5* mutants. We refer to these sets as PIF-induced and PIF-repressed, respectively. In order to evaluate whether our model of PIF activity could describe the dynamics of PIF targets other than *ATHB2* and *IAA29* in light:dark cycles, we used microarray timeseries data available from the DIURNAL database (Mockler *et al*, 2007). This database includes microarray data sampled across 2 days at 4-h time resolution in diverse conditions and has previously been used to assess interactions between circadian and light signalling (Dalchau *et al*, 2010). Clustering of transcript dynamics for genes identified as PIF-induced revealed two large and coherent clusters of genes; genes within each cluster shared condition-specific transcript dynamics across 6 conditions, including SDs and LDs (see Supplementary Information for details of analysis; Frey & Dueck, 2007). These genes comprised 191 of the 699 PIF-induced genes, including the known examples of *ATHB2* and *IAA29*, and showed significant overlap with PIF4-bound and PIF5-bound genes, relative to all PIF-induced genes (*P* < 10^−8^, Supplementary Fig S13A). Two additional PIF target species were introduced into the model to represent these two clusters. Their dynamics could be matched in a straightforward way by fitting only five PIF target-specific parameters, as shown in Fig4I–L for the comparison of SDs to LDs (microarray data from Michael *et al*, 2008b). Many of these genes also showed the SD-specific bimodal profile.

The generality of this model of PIF target regulation was further tested by considering genetic perturbations. Two classes of genetic perturbation are of particular interest—mutants with a defective EC (i.e. with the clock regulator of *PIF* transcription removed) and mutants with a defective circadian clock. In all cases, the model matched available data sets. In the first class are *elf3* and *lux* mutants (Nusinow *et al*, 2011) where qPCR time course data are available for *PIF4, ATHB2* and *IAA29* transcripts in an *elf3* mutant (Nomoto *et al*, 2012b; Supplementary Fig S9A–C) and microarray time course data are available in a *lux* mutant (Michael *et al*, 2008a; Supplementary Fig S9G–J). In the second class are the *prr9;prr7;prr5* triple mutant and *LHY* overexpressor [*LHYox*, also referred to as *lhy1* (Schaffer *et al*, 1998)]. For this class, qPCR time course data are available for *PIF4, ATHB2* and *IAA29* transcripts in a *prr9;prr7;prr5* mutant (Nomoto *et al*, 2012b; Supplementary Fig S10A–C), and microarray time course data are available in the *LHYox* mutant (Michael *et al*, 2008a; Supplementary Fig S10D–G). The consistency of model simulations with experimental data for the identified PIF-induced transcripts under diverse perturbations suggests that PIF4 and PIF5 are the dominant regulators of these transcripts in light:dark cycles.

We next considered how transcripts identified as being repressed by PIFs might be regulated in light:dark cycles. Therefore, we clustered these transcripts according to the similarity of their dynamics in the microarray data, identifying four clusters with consistent dynamics across six conditions, including a total of 209 of the 755 PIF-repressed genes (Supplementary Fig S13B; see Supplementary Information for details of analysis). The dynamics of these transcripts revealed the expected photoperiod effect, with high levels during the light period and reduced levels during the dark period (Supplementary Fig S14). As with the PIF-induced genes, the model captured the change in dynamics between SDs and LDs. However, in contrast to the case for PIF-induced genes, the model was not able to match some changes in transcript dynamics under genetic perturbations, especially in the case of the *LHYox* mutant (Supplementary Fig S15). This might reflect a role for other factors in the mechanism by which PIFs repress transcription.

In summary, we have constructed a model of PIF activity that is able to describe the dynamics of PIF-induced transcripts across photoperiods and in clock mutants. Analysis of microarray data allowed identification of 191 PIF-induced and 209 PIF-repressed transcripts with dynamics that are consistent with a model of PIF regulation across multiple conditions (Supplementary Tables S1 and S2). Thus, the modelled PIF dynamics are sufficient to coordinate the photoperiod response of plants far beyond the particular target genes considered previously.

### Modelling illustrates that PIF activity is not confined to the end of night

The model outlined above was created to describe the regulation of *ATHB2* and *IAA29* mRNA by PIF proteins. *ATHB2* and *IAA29* transcript levels were previously shown to rise towards the end of night in SDs, suggesting that PIF activity is highest at this time (Nomoto *et al*, 2012b). Model simulations not only suggested that PIF4 and PIF5 protein levels rise during the night in SDs, as described above, but also predicted significant amounts (∽50% of the peak level in SDs) of PIF protein during the day in all photoperiods (Supplementary Fig S11C).

In the model, the simulated daytime PIF protein levels result from the increase in *PIF4* and *PIF5* transcript during the day (peaking at ∽ZT2-6), which counteracts phyB-mediated PIF protein degradation during the light period. This model prediction was somewhat unexpected, as constitutively expressed PIF protein is strongly depleted by phyB (Nozue *et al*, 2007; Kumar *et al*, 2012; Lee & Thomashow, 2012). However, recent analysis of PIF4 protein levels under the control of its native promoter support this possibility (Yamashino *et al*, 2013; Bernardo-García *et al*, 2014). This suggests that in light:dark cycles, the turnover of PIF proteins by active phyB is not required for the observed diurnal dynamics of PIF targets. Indeed, it is known that other light-activated pathways act redundantly to repress PIF activity during the day, as represented in the model by the “Interactor” class of proteins.

While it is clear that PIF activity is strongly repressed during the day, reductions in PIF-induced target expression have been observed in *pif* mutants during the day and in constant light (Nomoto *et al*, 2012a,b; Koini *et al*, 2009; Sun *et al*, 2012). This suggests that PIF proteins may not be completely degraded in the light and therefore may retain some residual activity. In order to test this idea further, we inspected the dynamics of the clusters of PIF targets (as identified previously, see above; Supplementary Information) in constant light (LL) conditions under clock-entraining temperature cycles. Clear rhythms of PIF targets in these conditions are observable, with PIF-induced and PIF-repressed transcripts in phase and antiphase, respectively, with the phase of the *PIF4* and *PIF5* transcript rhythms (Supplementary Fig S16).

If the degradation of PIF proteins during the day is not required for the observed dynamics of PIF activity in light:dark cycles, then removal of phyB should not affect the dynamics of PIF targets in these conditions. We assessed this possibility by inspecting the dynamics of the clusters of PIF targets (as identified previously, see above) in the *phyB* mutant (Supplementary Fig S17). The qualitative dynamics of both PIF-induced and PIF-repressed targets are unchanged in the *phyB* mutant, with a rapid increase and decrease in PIF-induced and PIF-repressed transcripts during the day, respectively. This is consistent with previous observations of several canonical PIF-induced transcripts, including *ATHB2* and *IAA29* (Nomoto *et al*, 2012a,b; Yamashino *et al*, 2013), demonstrating that *phyB* acts redundantly with other light-signalling components to repress PIF activity in these conditions. Interestingly, a significant increase in PIF-induced transcript levels is observed during the night in the *phyB* mutant (Supplementary Fig S17A). This may explain the long hypocotyl phenotype of this mutant in light:dark cycles despite phyB's apparent redundancy in repressing PIF activity during the day and suggests that phyB regulates PIF activity during the night through a separate mechanism.

In conclusion, the model reconciled apparently conflicting observations of PIF activity during the day in wild-type plants, with the rapid, phyB-dependent degradation of constitutively expressed PIF proteins. The presence of PIF proteins during the day suggests that they might play a regulatory role during this time, a possibility which is further highlighted by our observations of PIF-dependent *FT* expression (see below).

### Linking molecular regulation to flowering time and hypocotyl elongation

**Figure 5:**
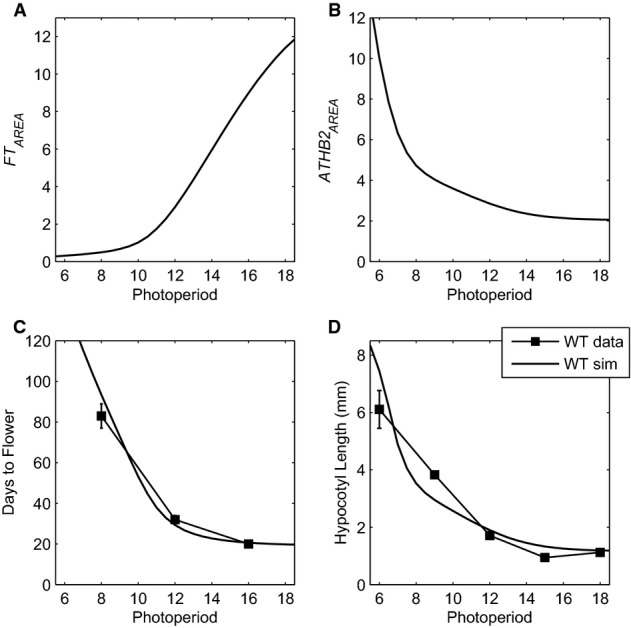
Model describing the photoperiod response of flowering and hypocotyl elongation in WT plants

*ATHB2* expression provides a molecular correlate of hypocotyl elongation across multiple conditions (Kunihiro *et al*, 2011), in a similar manner to *FT* expression in the flowering pathway (Salazar *et al*, 2009). The model simulates the nonlinear changes in average *ATHB2* mRNA and *FT* mRNA levels across photoperiods (Fig5A and B). The absolute values of hypocotyl length and flowering time vary among laboratories, so we used simple mathematical functions to relate *FT* and *ATHB2* mRNA levels to flowering and hypocotyl elongation. These represent the complex developmental mechanisms of the vegetative-to-inflorescence transition and the biophysics of elongation growth and can readily be recalibrated for the conditions of particular studies (as described in Supplementary Information). The full model can thereby simulate photoperiod responses for these two phenotypes in wild-type plants (Fig5C and D; Corbesier *et al*, 1996; Niwa *et al*, 2009; Kunihiro *et al*, 2011). As the model fully couples the circadian clock to both output pathways, it also simulates both phenotypes in clock-mutant lines. The dynamic regulation described above, for example, naturally matched the delayed flowering and long hypocotyls in *prr9;7* mutants (Supplementary Table S3). However, the changing seasons are also accompanied by changes in ambient temperature, which modifies the expression of both phenotypes.

Simulated levels of *FT* and *ATHB2* mRNA (as calculated by taking the area under the curve) across multiple photoperiods.

Using *FT*_*AREA*_ values, the number of days to flower was calculated and compared to data from Corbesier *et al* (1996) (see Supplementary Information). Error bars represent standard deviation. Simulated flowering: solid black line, filled squares; data: dashed black lines, empty squares.

Same as in (C) using *ATHB2*_*AREA*_ to calculate hypocotyl length. Data taken from Kunihiro *et al* (2011) (error bars were unavailable).

### PIF-mediated temperature control of hypocotyl elongation

**Figure 6:**
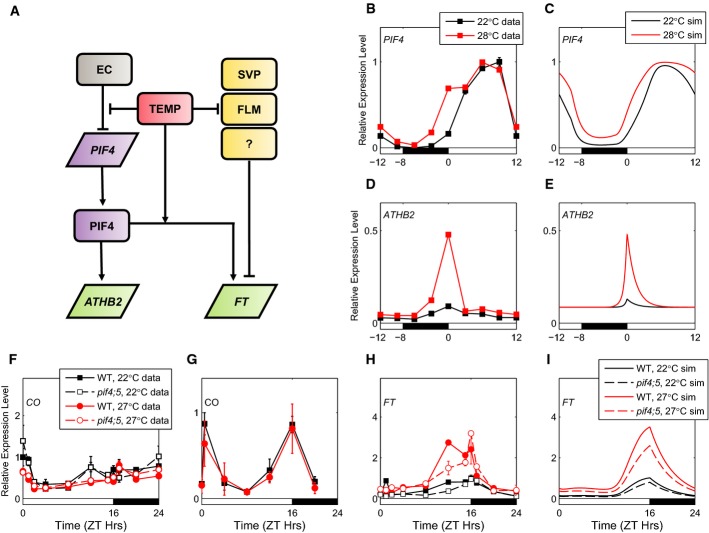
Coordinated regulation of *ATHB2* and *FT* by PIF4

Warmer temperatures result in earlier flowering and longer hypocotyls (Gray *et al*, 1998; Balasubramanian *et al*, 2006). It has been suggested that PIF4 plays a role in the temperature sensitivity of hypocotyl growth through stimulation of *ATHB2, IAA29* and other hormone-related genes (Koini *et al*, 2009; Nomoto *et al*, 2012a) and in the temperature sensitivity of flowering through stimulation of *FT* (Kumar *et al*, 2012). The present model allowed us to assess how PIF4 may achieve this combined regulation (Fig6). Recently, it has been shown that EC repression of *PIF4* expression is relieved at higher temperatures (e.g. 28°C versus 22°C), leading to higher *PIF4* levels during the night in these conditions. The model reproduced these observations through mild temperature modulation of the affinity of the EC for the *PIF4* promoter, resulting in less EC repression of *PIF4* expression at the higher temperature (Fig6B–E). Altered affinity is sufficient to prevent full repression of *PIF4* mRNA in the early night and to allow a 2–3 h earlier rise of *PIF4* before dawn at 28°C; thus, warm temperature in LDs leads to night-time levels of *PIF4* mRNA that are similar to the level in SDs at 22°C. In both model and data, this results in 3- to 4-fold higher accumulation of *ATHB2* at dawn, qualitatively consistent with the increased hypocotyl elongation observed at the higher temperature (Nomoto *et al*, 2012b).

Schematic of the regulation of *ATHB2* and *FT* expression by PIF4 and temperature.

Comparison of data and model simulations for temperature response of *PIF4* (B, C) and *ATHB2* (D, E) mRNA in LDs (16L:8D) in the WT. Data from Nomoto *et al* (2012a) were normalised such that data and simulation have equal peaks at 28°C (22°C data: black lines, filled squares; 22°C simulation: black lines; 28°C data: red lines, filled squares; 28°C simulation: red lines).

*CO* mRNA data in LDs in WT and the *pif4;pif5* mutant at 22 and 27°C (WT 22°C data/simulation: as in (B–E); *pif4;pif5* 22°C data: dashed black lines, open squares; WT 27°C data: red lines, filled circles; *pif4;pif5* 27°C data: dashed red lines, open circles).

CO protein data in LDs (16L:8D) in WT at 22 and 27°C (as in F).

*FT* mRNA data in LDs in WT and the *pif4;pif5* mutant at 22 and 27°C (as in F).

*FT* mRNA simulation in LDs in WT and the *pif4;pif5* mutant at 22 and 27°C (WT 22°C simulation: as in B–E; *pif4;pif5* 22°C simulation: dashed black lines; WT 27°C simulation: red lines; *pif4;pif5* 27°C simulation: dashed red lines). Data information: Plants were grown in 16L:8D cycles for 13 days (F, H) or 10 days (G) at 22 and 27°C. Error bars represent standard error in (F–H) and standard deviation in (B, D). Note: error bars smaller than symbols in (D).

### PIF-dependent control of the flowering regulators

Extending PIF-dependent regulation to *FT* in the model highlighted two areas that contrast with the relatively simple link from EC activity to hypocotyl elongation. We first tested whether *FT* displayed a PIF-dependent change in dynamics, similar to that seen in *IAA29* and *ATHB2* at the higher temperature in LDs. Wild-type and *pif4;pif5* double mutant plants were grown for 13 days in 16L:8D cycles at 22 and 27°C and harvested at 4-h intervals (with additional time points around dawn and dusk), and *CO* and *FT* RNA levels were measured by qRT–PCR. The *CO* mRNA profile was unaffected by the *pif* mutations (Fig6F). Moreover, no PIF-dependent peak in *FT* expression at dawn was observed at either 22 or 27°C in LDs (Fig6H). Instead, *PIF4* and *PIF5* stimulated *FT* expression in the wild-type plants at ZT8-12. Interestingly, the double mutants showed lower expression at both temperatures. The absence of *FT* induction at dawn is consistent with results from the *elf3* mutant, in which *PIF4* and *PIF5* transcript levels during the night are also increased (Nusinow *et al*, 2011; Lu *et al*, 2012; Nomoto *et al*, 2012b) without a corresponding increase in *FT* expression at this time (Kim *et al*, 2005; Lu *et al*, 2012). This contrasts with the dynamics of the canonical PIF targets such as *ATHB2* and suggests that PIF activity at *FT* is temporally modulated by other factors. Since changes in PIF activity during the night do not affect *FT* expression at either temperature (Fig6H), co-activation of *FT* by PIFs and a light-dependent factor is one possible mechanism for this modulation. A candidate for such a light-dependent factor is CO, as ChIP analysis has shown that PIF4 and CO bind to the *FT* promoter in overlapping regions (compare locations of FT-c1/c/15 from Kumar *et al*, 2012 with amplicons 12 to 14 from Song *et al*, 2012). If PIF4 and CO assemble at the *FT* promoter, then they are likely to interact when both are present. Alternatively, other light-dependent activators of *FT* have been also identified [e.g. the CRY2-interacting bHLH (CIB) family of transcription factors (Liu *et al*, 2013)]. Light-dependent regulation of PIF activity at the *FT* promoter was sufficient for the model to recapitulate the observed, phase-dependent effect of the PIFs on *FT* at the reference temperature, 22°C (Fig6I).

### The contribution of PIFs to temperature-induced FT expression

Having accounted for the temporal distinction between the effects of PIFs on *FT* and on canonical targets, we next tested whether increased PIF protein levels at 27°C would explain the temperature response of *FT*, as it had for the canonical target genes. Our transcript data showed a 2- to 3-fold increase in *FT* expression levels at 27°C (Fig6H), consistent with the early flowering of wild-type plants at this temperature. The strongest effects were at ZT8-16. The *pif4;pif5* mutant clearly retained temperature sensitivity of *FT* expression in LDs (Fig6H; Supplementary Dataset S5). Our data collected in SDs also showed temperature sensitivity of *FT* expression in the *pif4* mutant (Supplementary Fig S18; Supplementary Dataset S6). This is consistent with previous reports (Kumar *et al*, 2012; Thines *et al*, 2014), though we did not detect a reduction in peak *FT* levels in the *pif4* mutant. This suggests that additional, temperature-sensitive regulators of *FT* play a role in these conditions.

As the time of the greatest temperature response in *FT* (ZT8-16) coincides with when CO protein is active, we reasoned that temperature regulation of CO activity might explain these effects. Measured *CO* transcript levels did not change with temperature (Fig6F; Supplementary Dataset S3) [as observed previously, (Kumar *et al*, 2012; Thines *et al*, 2014)], so we measured HA-tagged CO protein in transgenic lines that expressed this transgene from the *CO* promoter. No difference in CO protein levels was observed between the two temperatures (Fig6G; Supplementary Dataset S4).

Recently, it has been shown that *FLOWERING LOCUS M* (*FLM*) and *SHORT VEGETATIVE PHASE* (*SVP*) are involved in mediating the temperature sensitivity of *FT* expression in the temperature range 5–27°C, and in both SDs and LDs (Lee *et al*, 2013; Posé *et al*, 2013). However, no single, dominant component was identified, as mutants in each of these factors retain some temperature sensitivity (Lee *et al*, 2013). The action of these regulators can be modelled by introducing a uniform activation of *FT* expression at 27°C, leaving the model behaviour at 22°C unchanged. With this simple assumption, the model is able to reproduce the observed change in *FT* dynamics at 27°C (Fig6I).

Thus, PIF4 plays qualitatively distinct roles in the transcriptional regulation of *ATHB2* and *FT*. An external coincidence model successfully describes the response of *ATHB2* to photoperiods and accommodates known transcriptional regulation of *PIF4* to describe its response to temperature. In contrast, the effects of PIF4 on *FT* are limited to the daytime and are mediated by a mechanism that is apparently independent of transcriptional regulation of *PIF4*.

## Discussion

Linking the circadian clock model to two well-characterised output pathways has accomplished three goals. First, in the photoperiodic CO-*FT* pathway, we proposed circadian mechanisms to regulate central components *CDF1* and *FKF1* (Figs1 and 2A), with experimental validation (Fig2B). This model refinement removed the need for data inputs present in previous models of flowering time that limited their utility (Salazar *et al*, 2009; Song *et al*, 2012). Second, to create the primary model for photoperiodic control of hypocotyl elongation, we linked light-dependent regulation of PIF proteins to circadian regulation of *PIF* transcription (Figs1 and 4A). Third, to examine crosstalk between these two pathways, we tested PIF-dependent regulation of *FT* expression (Figs1 and 6A). Consolidating these diverse experimental data within a mathematical model extended our understanding of this system in several ways.

### Refinement of the photoperiodic CO-FT pathway

**Figure 7:**
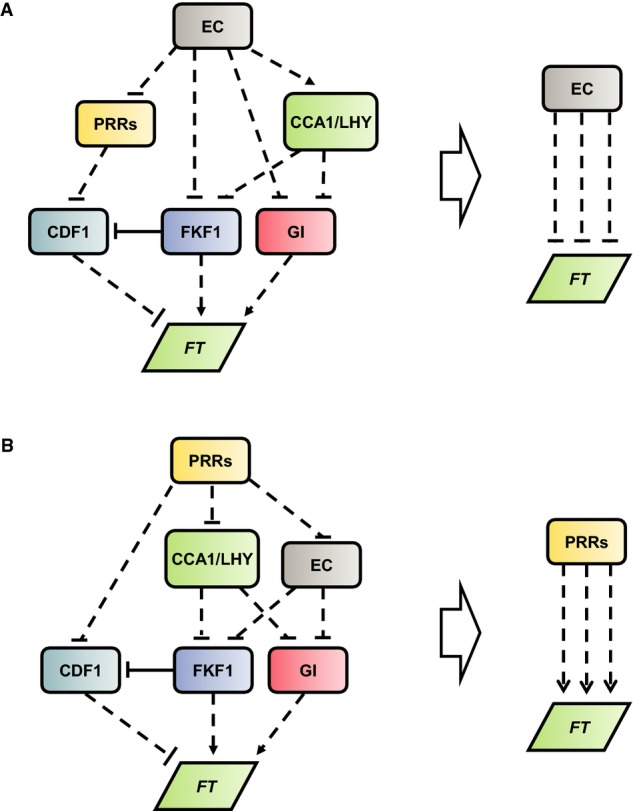
Coherent feed-forward networks coordinate the regulation of FT by the circadian clock As highlighted in the discussion, the flowering system is based on a combination of coherent feed-forward pathways, allowing a single component to play multiple reinforcing roles in the system (Mangan & Alon, 2003). This is highlighted here in the case of the EC and the PRRs.

The new model links *CDF1* and *FKF1* mRNA to the clock, allowing multiple photoperiods and genetic perturbations to be simulated. *CDF1* transcription was known to be controlled by the PRR proteins, while the regulation of *FKF1* mRNA appeared similar to *GI* (Nakamichi *et al*, 2007, 2012; Niwa *et al*, 2007; Ito *et al*, 2008). A good qualitative match to *CDF1* and *FKF1* mRNA data in WT, *cca1;lhy* and *prr9;7* was achieved with *CDF1* and *FKF1* under the dual regulation of CCA1/LHY and of PRRs or the EC, respectively (Fig2 and Supplementary Figs S1 and S2). Subsequent ChIP assays showed significant enrichment of CCA1 at CBS/EE motifs in the *CDF1* and *FKF1* promoters (Fig1B and Supplementary Fig S3). Thus, our data validated the model prediction that CCA1 is a regulator of *CDF1* and *FKF1* transcription. The refinement of our model to incorporate clock control of *CDF1* and *FKF1* transcription allowed us to evaluate how the clock coordinates the timing of flowering with the photo-period and demonstrated a striking coordination in the regulation of *FT* by clock components (Fig7). At each step of the flowering pathway, the EC acts to inhibit *FT* mRNA accumulation and prevent flowering (Fig7A), while the PRR proteins act to promote the floral transition by increasing the rate of *FT* transcription (Fig7B). Thus, these network motifs form part of a family of coherent feed-forward loops within the flowering system (Mangan & Alon, 2003).

The EC can repress *FT* expression through at least three partially redundant pathways, involving coordinated control of CDF1, FKF1 and GI levels.

Similarly, the PRRs can activate *FT* expression through at least three pathways. Rectangles denote protein species, and parallelograms denote transcript species. Solid and dashed lines indicate direct and indirect forms of regulation, respectively.

### Post-translational regulation of the flowering pathway

The role of GI-FKF1 in controlling CO and CDF1 stability to control *FT* expression is well established. However, two lines of evidence suggest a further role for GI in the regulation of CDF1 stability: first, our measurements of CDF1 protein in a CDF1ox/*gi* line suggest that CDF1 is more stable in the absence of *gi* than in the absence of *fkf1* and second, the decrease in *CO* expression in the *gi* mutant as compared to the *fkf1* mutant (Fig3D and E). The independent regulation of CDF1 by GI may be direct, for example with GI acting in complex with ZTL, and/or LKP2 (Ito *et al*, 2012), or indirect, as a result of GI's widespread regulation of other pathways. For example, GI is known to act antagonistically with ELF3 (Yu *et al*, 2008; Pokhilko *et al*, 2012).

### The model highlights PIF protein activity through a diurnal cycle

In line with published data, our model simulated a rise in PIF activity towards the end of the night in SDs (Fig4; Kunihiro *et al*, 2011; Nomoto *et al*, 2012b). The consistency of model predictions across a range of conditions then allowed us to use clustering analysis to identify putative targets of PIFs which display dynamics consistent with the model in light:dark cycles. Interestingly, the model was able to describe the essential differences between the clusters of PIF-induced genes, including the pattern of sensitivity to changes in conditions. In particular, transcripts with sharp peaks at dawn in SD (i.e. Cluster 1) are predicted to display enhanced sensitivity to the *lux* and *LHYox* mutations, in line with observations (Supplementary Fig S21). Together, these results demonstrate the widespread nature of transcriptional regulation undertaken by PIF4 and PIF5 in light:dark cycles.

The increase in PIF activity at the end of the night in SDs coincides with a high rate of hypocotyl growth (Nozue *et al*, 2007) and has been associated with transcriptional activation of phytohormone signalling pathways at this time (Michael *et al*, 2008a; Nomoto *et al*, 2012b). Our modelling and data analysis explained two further aspects of the regulation of PIF activity in light:dark cycles. We highlighted a SD-specific increase in PIF activity at the beginning of the night. Modelling suggested that the SD-specific increase in PIF-induced targets at the beginning of the night results from the coincidence of *PIF4* and *PIF5* transcript with darkness at this time in these conditions.

In addition, we reassessed the role of protein turnover in the diurnal regulation of PIF activity. Light-stimulated degradation of PIF protein by active phyB plays an important role in the de-etiolation response of dark-grown seedlings (Al-Sady *et al*, 2006), and measurements of PIF4 and PIF5 protein levels in constitutive overexpressors have demonstrated that this regulation also occurs in light:dark cycles (Nozue *et al*, 2007; Niwa *et al*, 2009; Kumar *et al*, 2012; Lee & Thomashow, 2012). However, our model suggested that the counteracting effect of increased levels of *PIF4* and *PIF5* transcript during the day may lead to significant levels of PIF protein at this time. Assessment of PIF target dynamics in microarray timeseries data supported this hypothesis, in line with recent data showing significant PIF4 protein levels during the daytime (Yamashino *et al*, 2013; Bernardo-García *et al*, 2014). In addition, the presence of PIF proteins during the day suggested that PIFs may play a regulatory role during this time. This was further highlighted by our analysis of PIF regulation of *FT* expression (see below).

### Temperature regulation of hypocotyl elongation and flowering time

At higher temperatures, hypocotyl growth is increased and flowering time is reduced. This response is mediated, in part, by components that are also involved in the photoperiod responses of both pathways. In the case of hypocotyl elongation, the increase in hypocotyl growth correlates with increases in the PIF targets *ATHB2* and *IAA29* (Nomoto *et al*, 2012a). This response appears to be mediated in part by an alleviation of EC repression of *PIF4* expression during the night at higher temperatures, with a resultant increase in *PIF4* transcript at this time (Mizuno *et al*, 2014). The model demonstrated that this mechanism was sufficient to understand the response of hypocotyl elongation to temperature.

In the case of flowering time, the reduction in flowering time at higher temperatures correlates with increases in *FT* expression (Kumar *et al*, 2012; Lee *et al*, 2013; Posé *et al*, 2013), and *FT* is required for the response of flowering to high temperature (Balasubramanian *et al*, 2006; Kumar *et al*, 2012). Recently, this sensitivity to temperature has been shown to be mediated in part by increased activation of *FT* by PIF4 (Kumar *et al*, 2012) and a reduction in SVP/FLM-dependent suppression of *FT* (Lee *et al*, 2013; Posé *et al*, 2013). A combination of modelling and experiments allowed us to examine how these elements are integrated with the regulation of *FT* expression by CO. This provided a mechanistic basis for the observation that the transcriptional response of *PIF4* to temperature during the night is not required for PIF4-dependent activation of *FT* (Kumar *et al*, 2012). Finally, model simulations demonstrated that changes in the activity of constitutive repressors of *FT* such as SVP and FLM with temperature are sufficient to explain the observed changes in *FT* expression in the range 22–27°C.

While we have focussed here on the effects of temperature on *FT,* we note that in some cases, changes in *FT* expression are much more subtle than the accompanying changes in flowering time. This is especially the case in the *pif* mutants, where large changes in flowering time can be accompanied by small changes in *FT* expression across a light:dark cycle (Thines *et al*, 2014). While the sensitivity of flowering time to changes in temperature requires *FT* (Balasubramanian *et al*, 2006; Kumar *et al*, 2012), it seems likely that additional temperature-sensitive stimuli are required downstream of *FT* in the floral induction pathway.

### Interactions between circadian- and light-regulated components provide a generalised mechanism for external coincidence

Taken together, our results demonstrate the importance of external coincidence as a mechanism for photoperiod sensing in plants. This mechanism requires the combined regulation of pathways by both the circadian clock and light. In several cases, a single component plays multiple roles at different points of the pathway. The extensive interconnectivity of these pathways requires that quantitative approaches be taken to disentangle the various regulations and identify gaps in our existing knowledge. This is especially the case in scenarios in which the complex dynamics of the circadian clock are altered.

The coordination of plant development and physiology by the circadian clock is not limited to flowering and hypocotyl elongation, but extends to processes as diverse as metabolism (Graf *et al*, 2010), cold tolerance (Fowler *et al*, 2005) and stomatal opening (Dodd *et al*, 2005). As our understanding of the circadian clock and its role in these pathways is further refined, it may be possible to develop an understanding of the role of the clock through multiple, interacting output pathways across the whole plant life cycle. Such a holistic approach may provide fresh insights into the contributions of the clock to plant fitness (Dodd *et al*, 2005) and suggest approaches to engineer aspects of plant physiology for improved growth in both existing and new environments.

## Materials and Methods

### Experimental methods

#### Growth conditions for RNA analysis

For the measurements of *CO* and *FT* transcript in LDs and SDs (Fig5F and H and Supplementary Fig S18), seeds of WT (Col-4, Columbia accession) and *pif4;5* plants were surface-sterilised, then 30–40 seedlings were sown on 55-mm-diameter plates containing half-strength MS media (Melford, Ipswich, UK), pH 5.8 and 1.2% agar without added sucrose. For the LD experiments (Fig5F and H), the seeds were stratified at 4°C for 3 days and then grown for 13 days in 16-h light:8-h dark cycles (100 μmol m^−2^ s^−1^ from cool white fluorescent tubes) at 22 and 27°C. Seedlings were harvested from triplicate samples at ZT0, 1, 2, 4, 8, 12, 15, 16, 17, 20 and 24 (ZT = zeitgeber time, ZT0 = lights on) into RNA*later* solution (Sigma-Aldrich, Gillingham, UK). For the SD experiments (Supplementary Fig S18), the seeds were stratified at 4°C for 3 days and then grown for 7 days in 8-h light:16-h dark cycles (100 μmol m^−2^ s^−1^ from cool white fluorescent tubes) at 22°C. Seedlings were then transferred to soil and grown at either 22 or 27°C for a further 21 days. At 27°C, nine whole rosettes were singularly harvested at ZT16 and twelve whole rosettes were singularly harvested at ZT8 (ZT = zeitgeber time, ZT0 = lights on) into RNA*later* solution. Triplicate rosettes were harvested at 22°C. In both experiments, the plants were left overnight at 4°C in the RNA*later* solution to allow full penetration into the tissue (Locke *et al*, 2005). The generation and growth of *CO:HA-CO* constructs have been previously described (Song *et al*, 2012).

#### RNA extraction

For the LD experiment (Fig5F and H), RNA was extracted from the plant tissue using the Illustra RNAspin 96 RNA isolation kit (GE Healthcare, Chalfont St. Giles, UK) manually, as described (Salvo-Chirnside *et al*, 2011). For the SD experiment (Supplementary Fig S18), RNA was extracted from the plant tissue using the RNeasy Plant Mini kit (Qiagen, Hilden, Germany) following the manufacturer's instructions. In both cases, purified total RNA (1 μg) was reverse-transcribed into cDNA using SuperScript VILO cDNA synthesis kit with oligo dT primers (Invitrogen/Life Technologies, Paisley, UK) according to the manufacturer's instructions. cDNA was diluted 1/10 and 1 μl used for subsequent qRT–PCR.

#### Gene expression analysis

qPCRs were set up using a liquid-handling robot (freedom Evo, TECAN, Reading, UK) and run in a Lightcycler 480 system (Roche, Burgess Hill, UK) using LightCycler 480 SYBR green master mix (Roche, Burgess Hill, UK) (for the *CO* measurements, Fig5F) or KAPA SYBR FAST qPCR kit (Kapa Biosystems, Massachusetts, USA) (for the *FT* measurements). Data were analysed with Roche Lightcycler 480SW 1.5 using relative quantification based on the 2^nd^ derivative maximum method. Each cDNA sample was assayed in triplicate. The primers used for *ACT7* were 5′-CAGTGTCTGGATCGGAGGAT-3′ and 5′-TGAACAATCGATGGACCTGA-3′; for *CO* were 5′-TAACAGTAACACAACTCAGTCC-3′ and 5′-CCTCGAAGCATACCTTATTGTC-3′; and for *FT* were 5′-CATTTTATGATACGAGTAACGAACGGTG-3′ and 5′-CACTCTCATTTTCCTCCCCCTCTC-3′. Transcript levels were normalised to *ACT7* expression (Hong *et al*, 2010). Expression analysis of *CO* and *FT* transcription in *35S:3HA-CO* constructs has been previously described (Song *et al*, 2012).

#### Chromatin immunoprecipitation (ChIP) assays

Chromatin immunoprecipitation was performed following the protocol in Nelson *et al* (2006) with modifications. Wild-type seedlings from the Col-0 (Columbia) accession were grown on ½ MS agar plates at 22°C for 14 days with 12-h white light:12-h dark cycles and harvested at ZT2. The chromatin was sheared to between 100 and 1,000 bp in a Bioruptor UCD 200 (Diagenode, Liege, Belgium) at high intensity for 10 min (cycles of 30 s on/30 s off) at 4°C after Lau *et al* (2011). An aliquot of the chromatin was reserved at this point as the input chromatin. Immunoprecipation used equilibrated Dynabeads® Protein A (Invitrogen/Life Technologies, Paisley, UK). The pre-cleared chromatin was transferred away from the beads and incubated with rotation over night at 4°C with a 1:1,000 dilution of anti-GFP (Abcam ab290; Abcam, Cambridge, UK). A new aliquot of equilibrated beads was then added and incubated with the chromatin solution for 2 h at 4°C with rotation and then washed with low salt, high salt and lithium chloride washes. The immunocomplexes were recovered from the beads by boiling for 10 min in the presence of 10% Chelex resin (Bio-Rad, Hemel Hempstead, UK) and the proteins removed using Proteinase K Solution (Invitrogen/Life Technologies, Paisley, UK) at 50°C. The reserved input chromatin was also processed in parallel with Chelex and Proteinase K and then purified using QIAquick PCR purification kit (Qiagen, Manchester, UK). qPCR on the ChIP and input DNA was performed in triplicate using Brilliant III Ultra-Fast SYBR® Green QPCR Master Mix (Agilent, Wokingham, UK) on a Mx3005P machine. The results were calculated so that percent input was equal to 100 × (*primer*_*efficiency*^*dcT*^) where dcT is the difference between the adjusted input cT and the ChIP sample cT. The input cT was adjusted to account for the dilution factor of the input chromatin. The primer efficiency was unique to each primer pair and is equal to 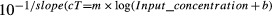. The primers used are listed in Supplementary Table S5.

#### Immunoblot analysis and protein quantification

To detect CDF1 protein in *35S:HA-CDF1* (Imaizumi *et al*, 2005) and *35S:HA-CDF1*/*gi-2* (Sawa *et al*, 2007) and CO protein in *CO:HA-CO* transgenic lines, plants from Col-0 (Columbia) accession were grown on Linsmaier and Skoog (LS) media (Caisson, Rexburg, Idaho, USA) containing 3% sucrose at 22 or 27°C with a fluence rate of 60 μmol m^−2^ s^−1^ in long-day (16-h light/8-h dark) and short-day (8-h light/16-h dark) conditions for 10 days. Seedlings were harvested at each time point on day 10 and were ground in liquid nitrogen for protein extraction. Whole proteins including the nuclear fraction were extracted with buffer containing 50 mM Tris, pH 7.4, 100 mM KCl, 10% glycerol, 5 mM EDTA, 1.0% NP-40, 0.5% deoxycholate, 0.1% SDS, 50 μM MG-132 and Complete protease inhibitor cocktail tablets (Roche, Indianapolis, Indiana, USA). Approximately 50 μg of extracted proteins was resolved in 12% SDS–PAGE gels and transferred to nitrocellulose membranes (Whatman, GE Healthcare, USA). HA-CDF1 and HA-CO protein was detected using anti-HA HRP conjugates (3F10, Roche, Indianapolis, Indiana, USA) and visualised with SuperSignal West Femto Maximum Sensitivity Substrate (Thermo Scientific, USA). For quantification of HA-CDF1 and HA-CO protein, non-specific binding of anti-HA around 25 kDa was used as a loading control. The method for protein quantification was described previously (Song *et al*, 2012).

### Data analysis

Data used in this study came from new experiments (see Materials and Methods, above) or from published sources (see Supplementary Table S4). While transcript data characterising the dynamics of the flowering and hypocotyl pathways are remarkably consistent across experiments and laboratories, care must be taken in interpreting results. In particular, since measurements were taken relative to different internal standards and with different normalisation across experiments in the literature, quantitative comparisons were only made within a particular experiment. For each transcript in each experiment, the unknown absolute scale of the measurements means that these data needed to be rescaled to be compared to model simulations, as detailed below.

As with previous models of flowering time, data for *CO* and *FT* mRNA taken from Imaizumi *et al* (2003) were normalised such that in LD conditions, *FT* peaked with relative WT expression level equal to 1 (at ZT16) and in 8L:16D *CO,* mRNA was normalised similarly (Salazar *et al*, 2009; Song *et al*, 2012). New data for *FT* mRNA in the WT and *pif4;5* backgrounds were similarly normalised. In order to obtain comparable relative expression levels of *CDF1* mRNA, raw data taken from Nakamichi *et al* (2007) and Niwa *et al* (2007) were normalised to the maximum expression of *FT* mRNA in LD. Due to the lack of experimental data in 8L:16D or 16L:8D cycles, *FKF1* mRNA data taken from SD = 10L:14D cycles were normalised such that relative WT expression levels peaked at 1 (Niwa *et al*, 2007). Since there is a lack of raw *FKF1* mRNA data across multiple photoperiods, we have refrained from making any direct comparisons of expression levels between *FKF1* mRNA and other components of the model. Relative protein levels for CDF1 and FKF1 were normalised such that the WT FKF1 protein peaked with relative expression level equal to 1 in LD, as in Song *et al* (2012). Supplementary Fig S4 shows the model simulations matched to the data sets described here that were used for optimisation for the components of the flowering pathway.

*PIF4* mRNA rhythmic data in WT, *elf3* and *prr9;prr7;prr5* were taken from Nomoto *et al* (2012b) (Fig4 and Supplementary Figs S9 and S10). *PIF4* and *PIF5* data in WT were taken from Nusinow *et al* (2011) in 8L:16D, 12L:12D and 16L:8D diurnal cycles and used for parameter optimisation (data not shown). Data were normalised, so the peak of relative WT expression was 1 in all photoperiods since the peak expression level of *PIF4* mRNA has been observed not to change greatly with photoperiod (Nomoto *et al*, 2012a). Data for the PIF transcriptional targets, *IAA29* and *ATHB2*, were taken from Nomoto *et al* (2012b) in 8L:16D and 16L:8D conditions and normalised to peak with a relative expression level of 1 in 8L:16D cycles (Fig4 and Supplementary Figs S9 and S10).

### Resources

Computational methods are described in detail in the Supplementary Information. The model is provided as Supplementary File S1 and will be available from the PlaSMo repository upon publication (www.plasmo.ed.ac.uk; identifier PLM_1010). Literature data used to parameterise and test the model are provided as Supplementary File S2. Data generated in this study are provided as Supplementary Datasets S1, S2, S3, S4, S5 and S6. In addition to the Supplementary data files, numerical data are available from the BioDare repository (www.biodare.ed.ac.uk; identifiers are given in Supplementary Table S7).

## Supporting Information

Supplementary Information

Supplementary References

Review Process File
