# Supplementary figures and images for: Linked circadian outputs control elongation growth and flowering in response to photoperiod and temperature
Source: Mol Syst Biol. 2015 Jan 19;11(1):776. doi: 10.15252/msb.20145766 (PMC4332151; doi:10.15252/msb.20145766)

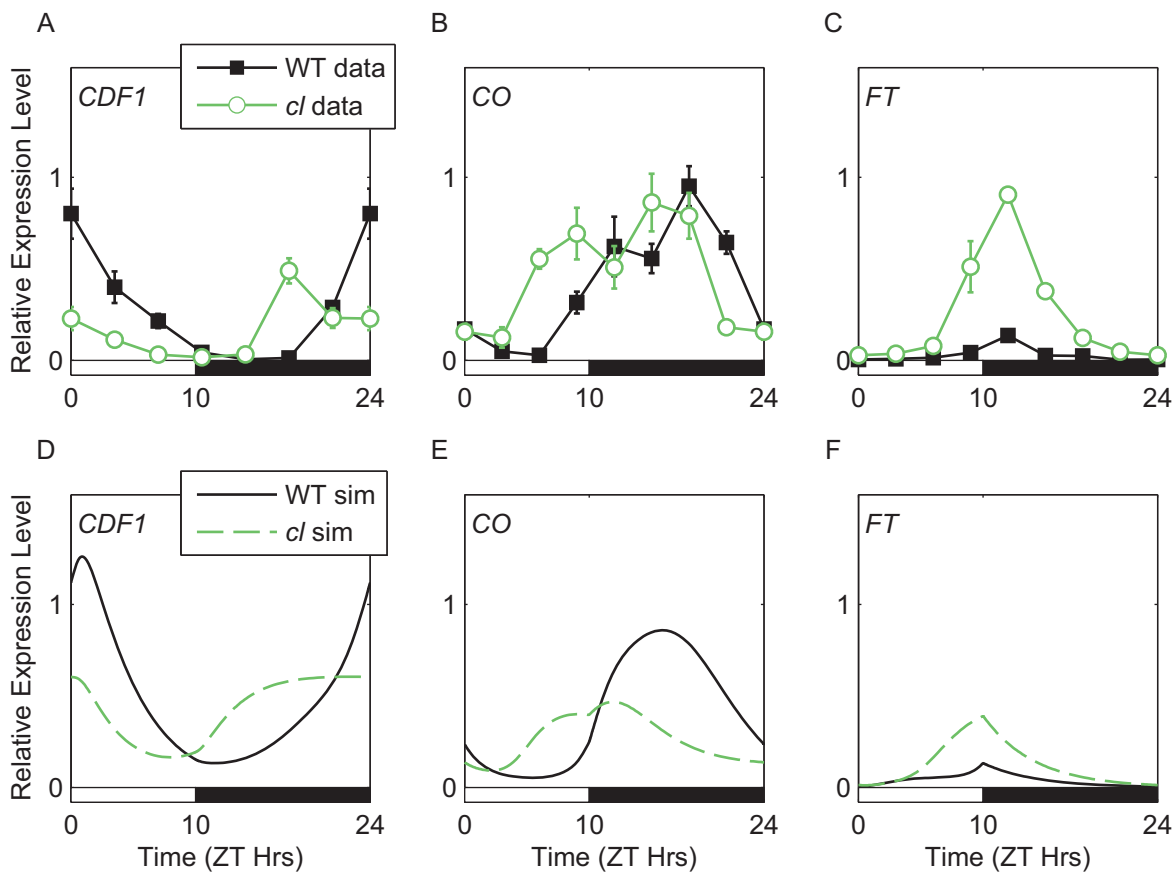

Supplement: Supplementary file 1 [file msb0011-0776-sd1.pdf]

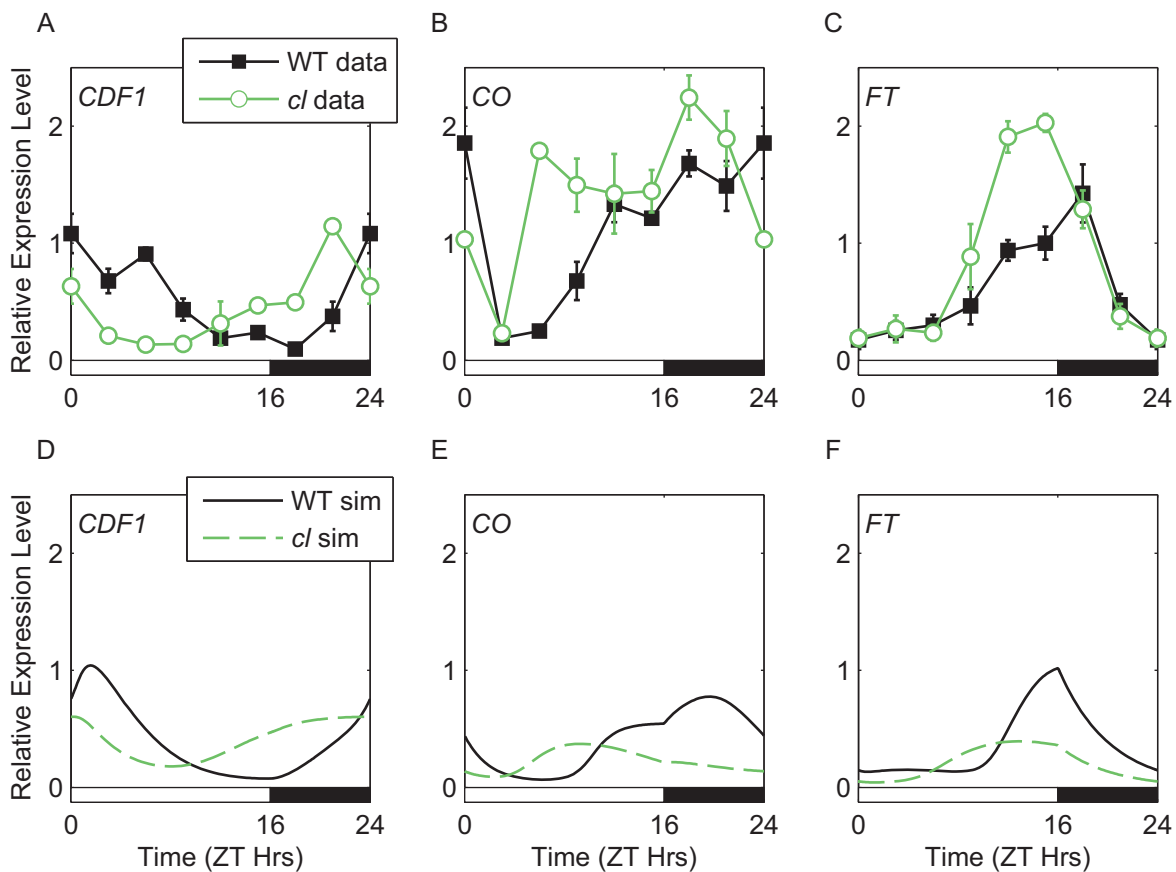

Supplement: Supplementary file 2 [file msb0011-0776-sd2.pdf]

A

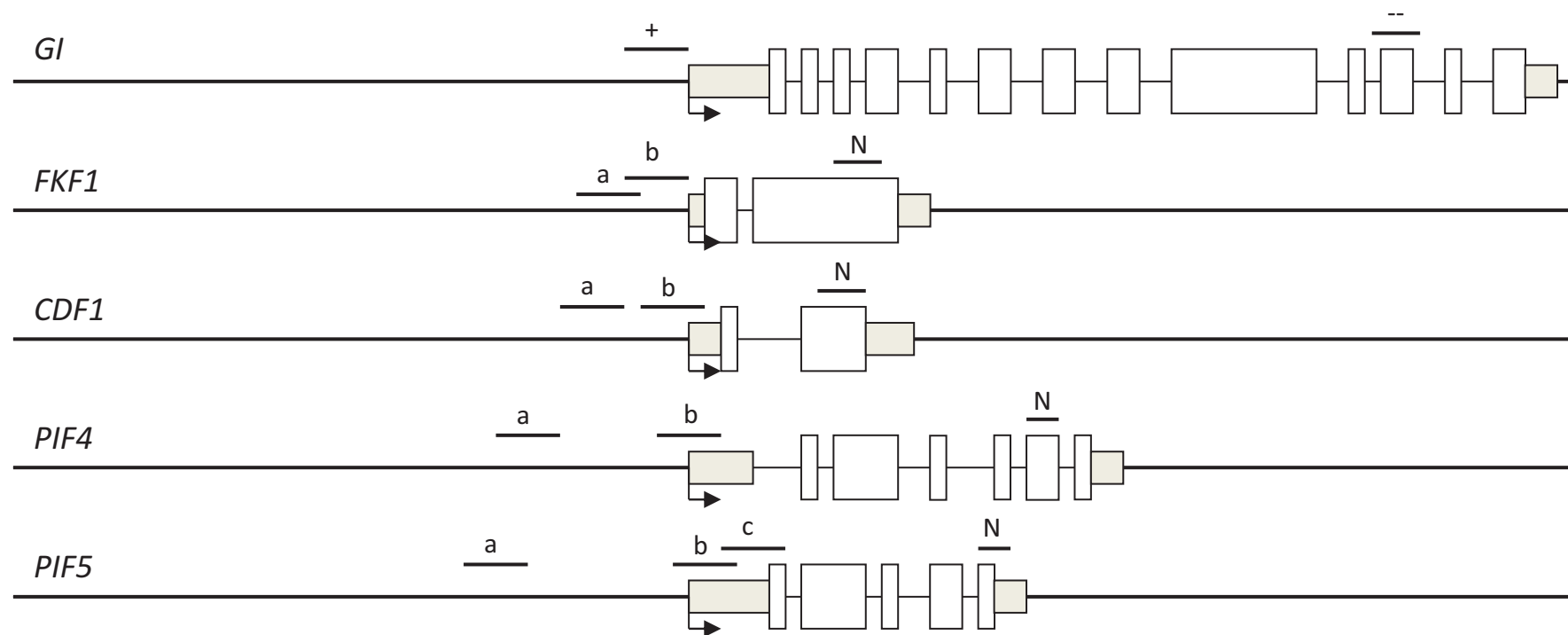

B

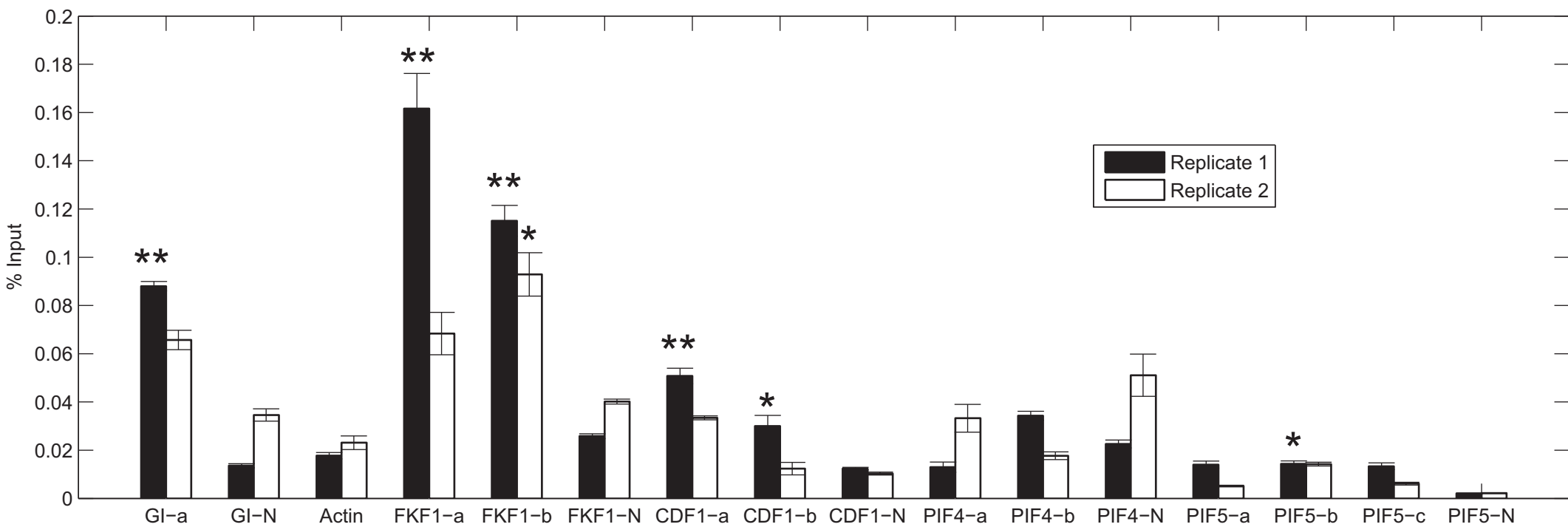

Supplement: Supplementary file 3 [file msb0011-0776-sd3.pdf]

Relative Expression Level

A

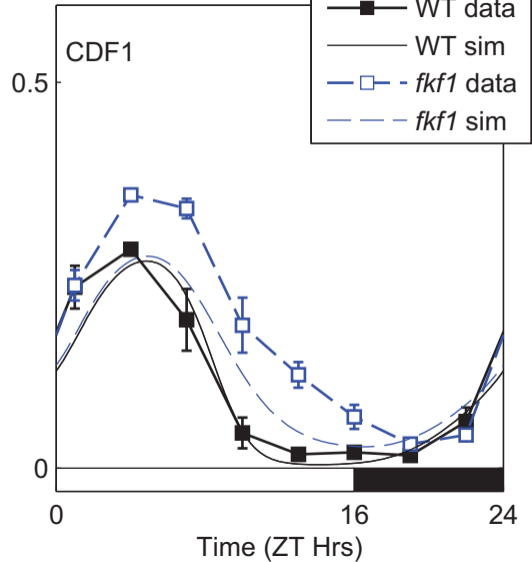

B

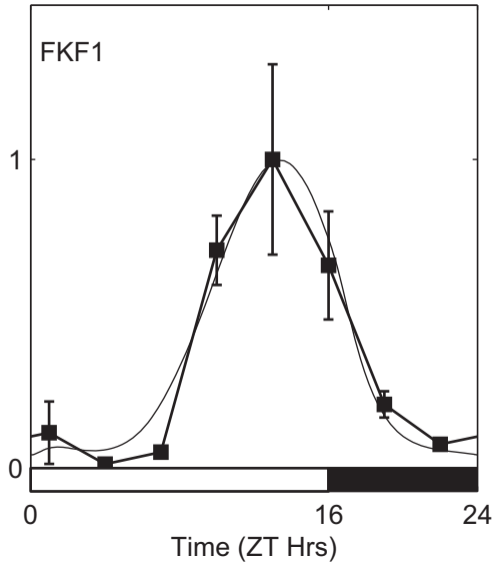

Supplement: Supplementary file 4 [file msb0011-0776-sd4.pdf]

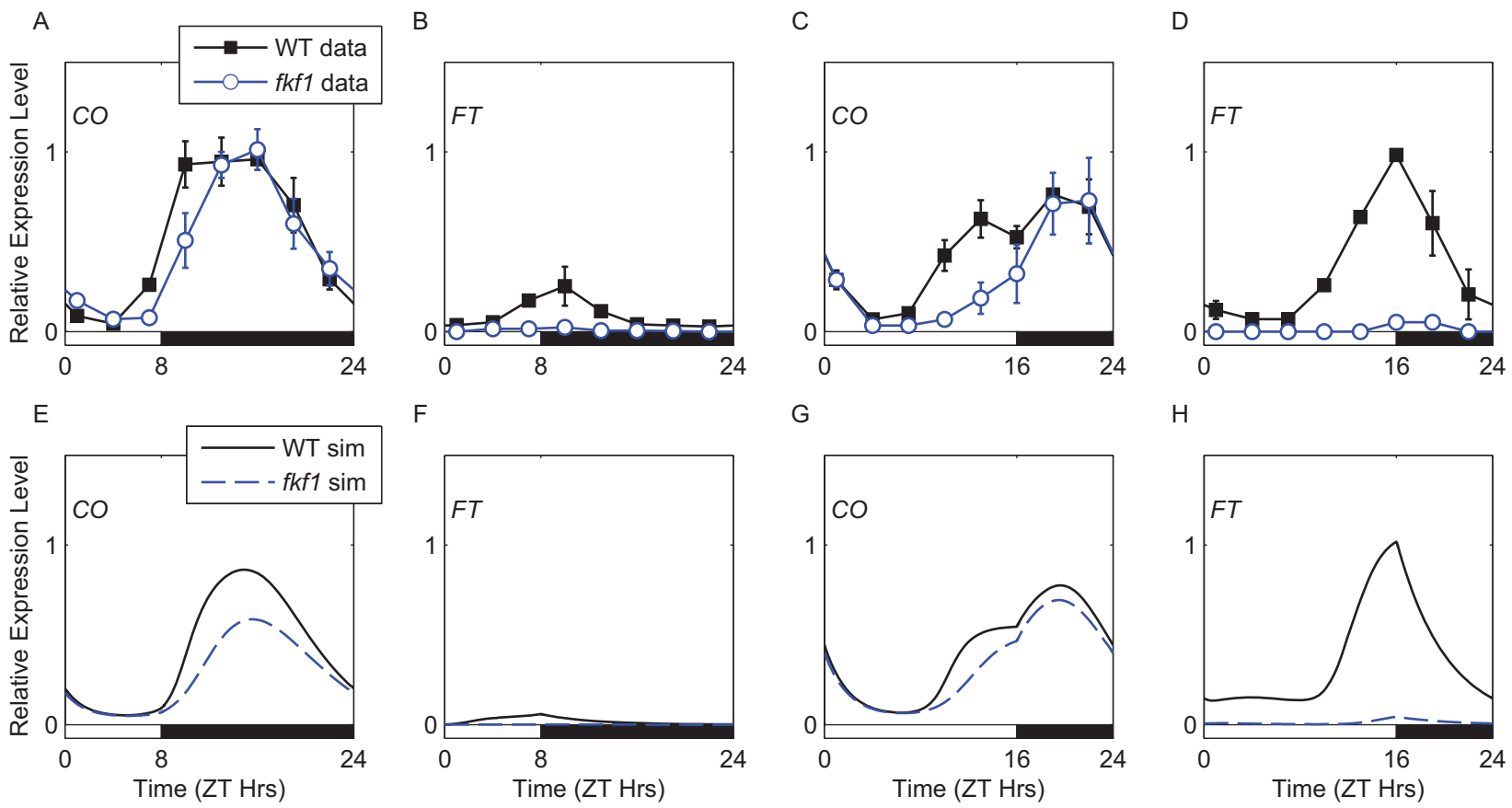

Supplement: Supplementary file 5 [file msb0011-0776-sd5.pdf]

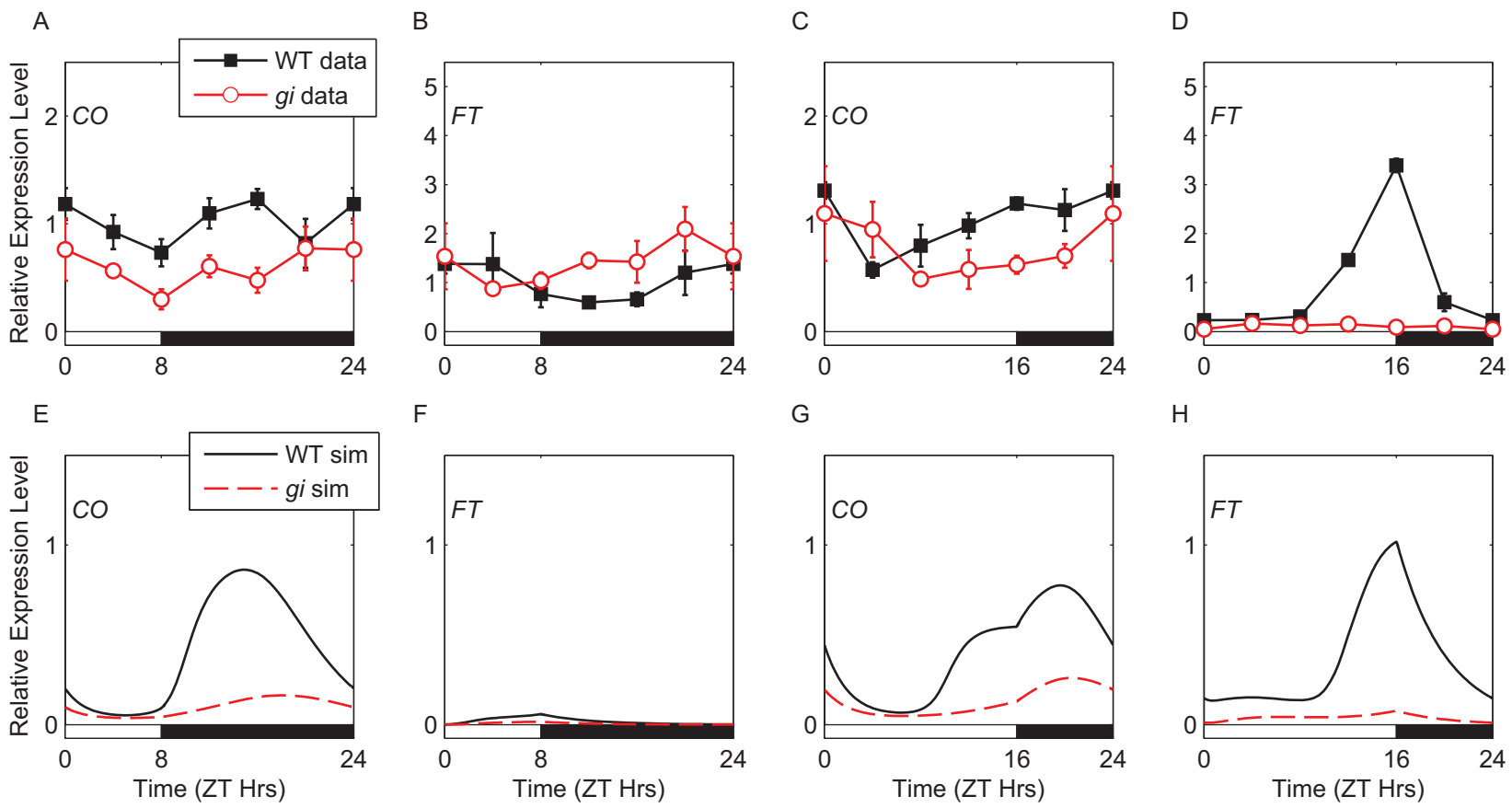

Supplement: Supplementary file 6 [file msb0011-0776-sd6.pdf]

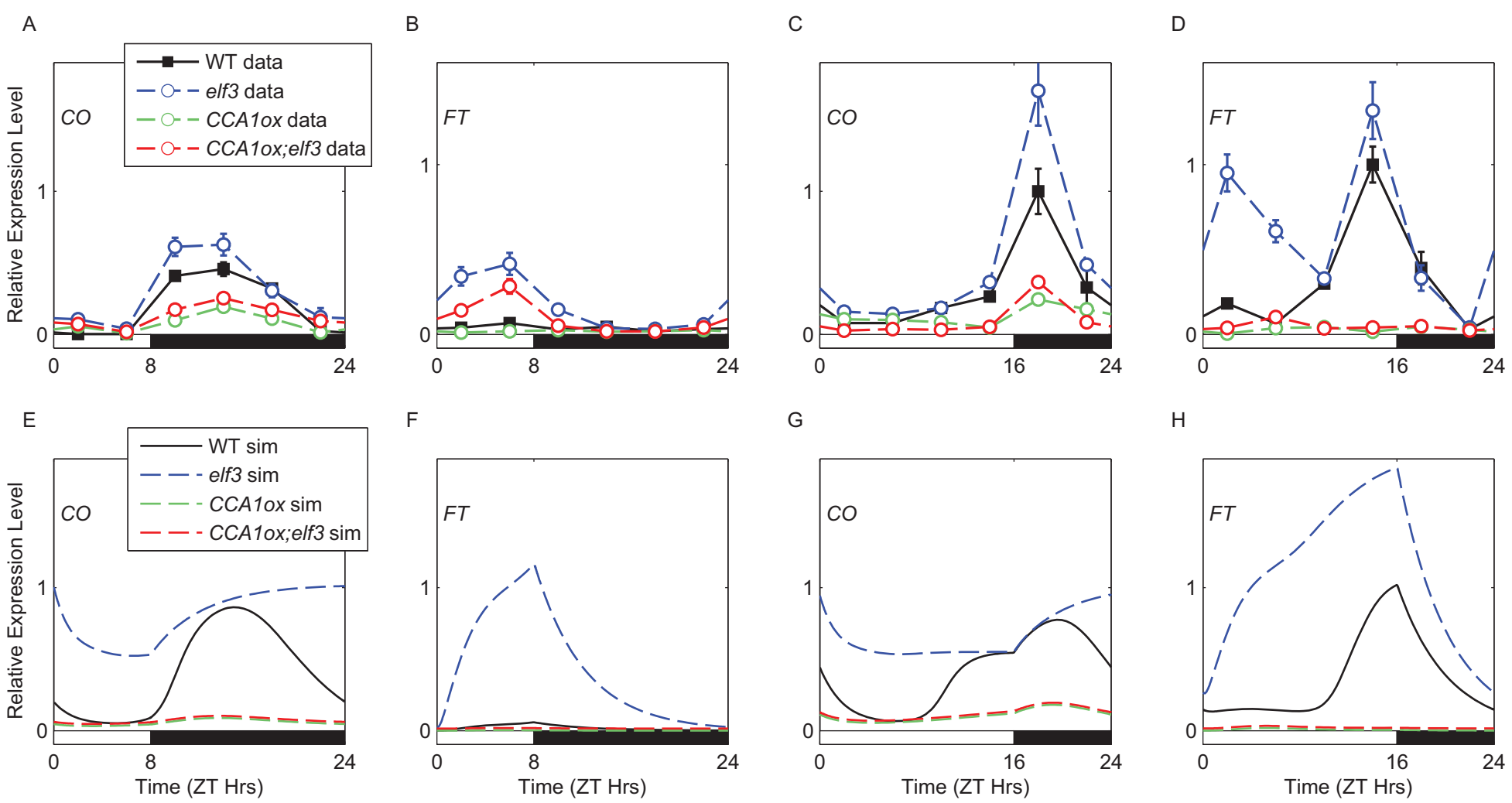

Supplement: Supplementary file 7 [file msb0011-0776-sd7.pdf]

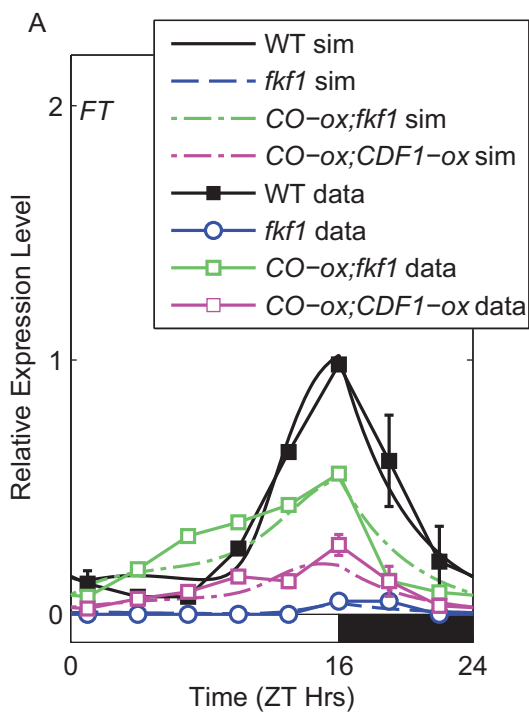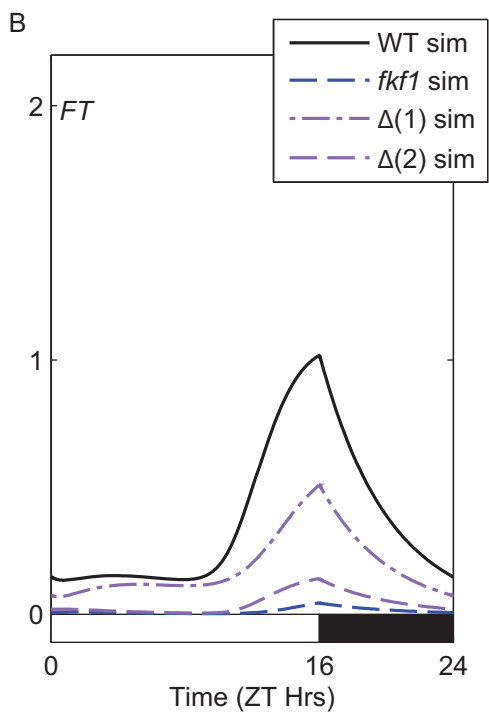

Supplement: Supplementary file 8 [file msb0011-0776-sd8.pdf]

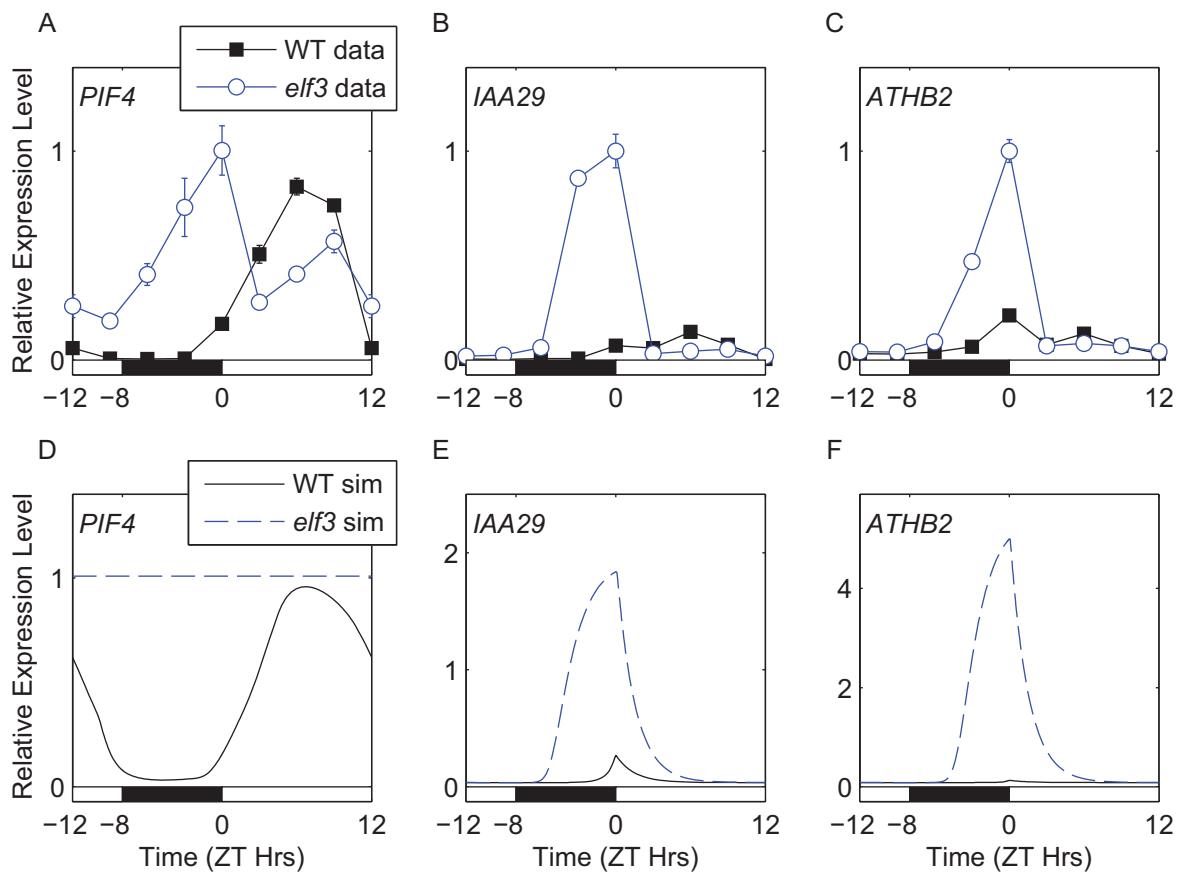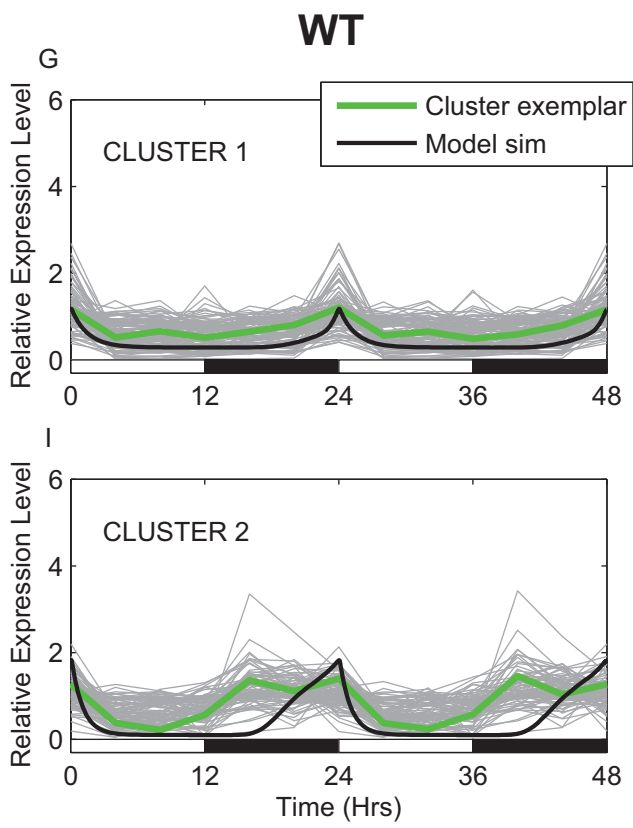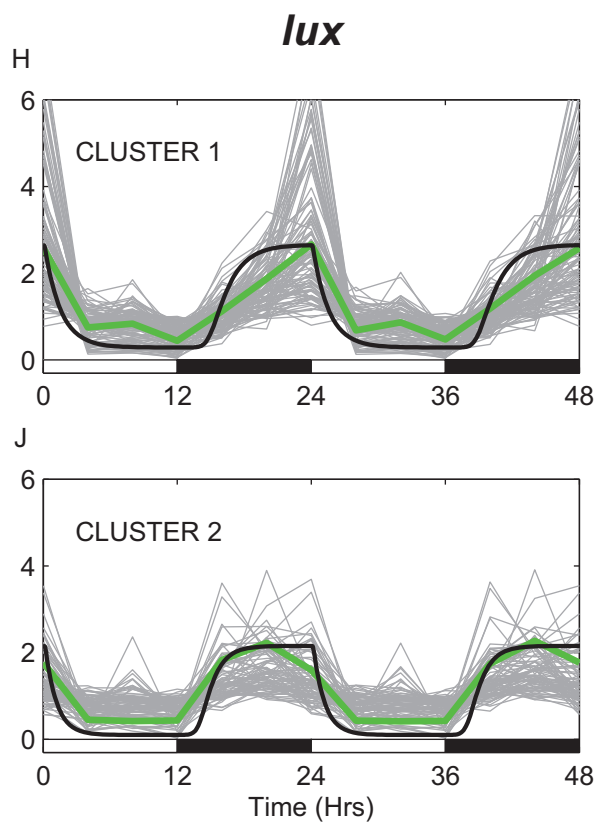

Supplement: Supplementary file 9 [file msb0011-0776-sd9.pdf]

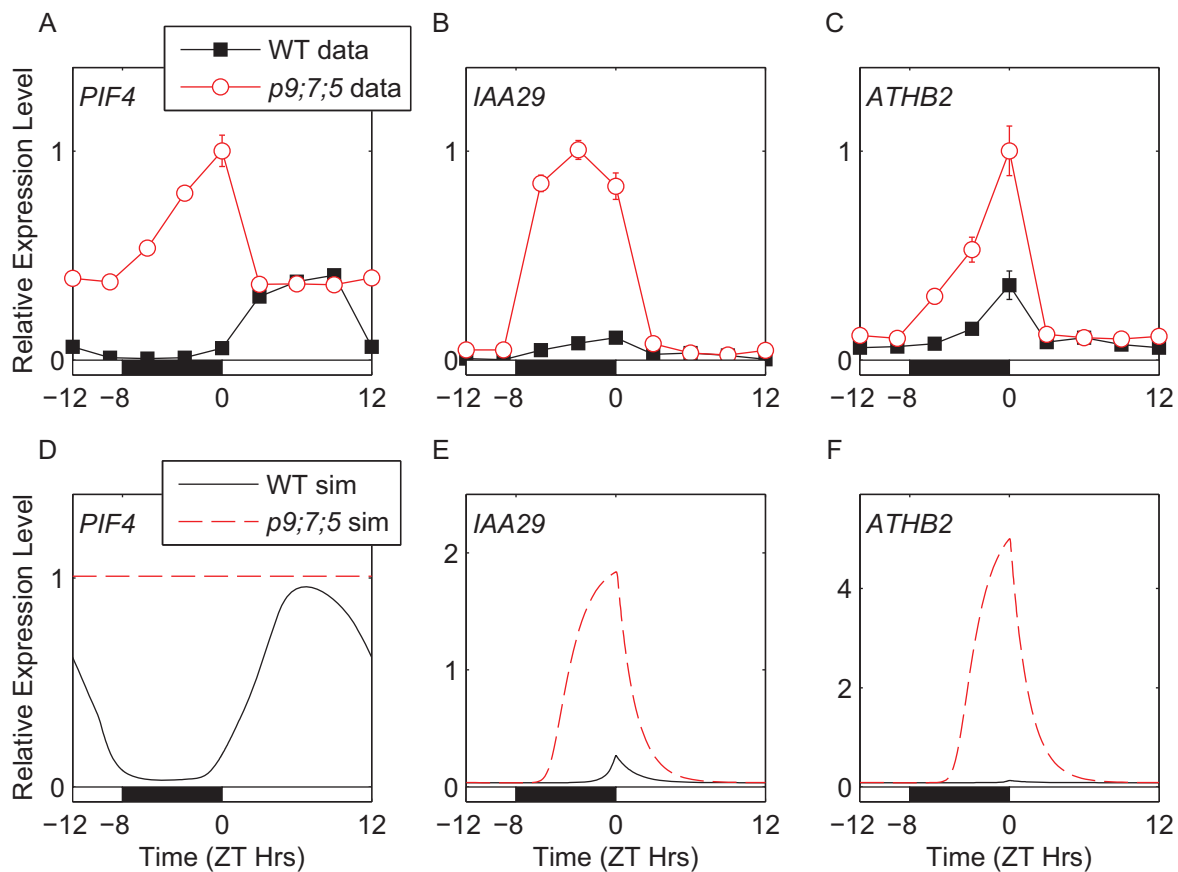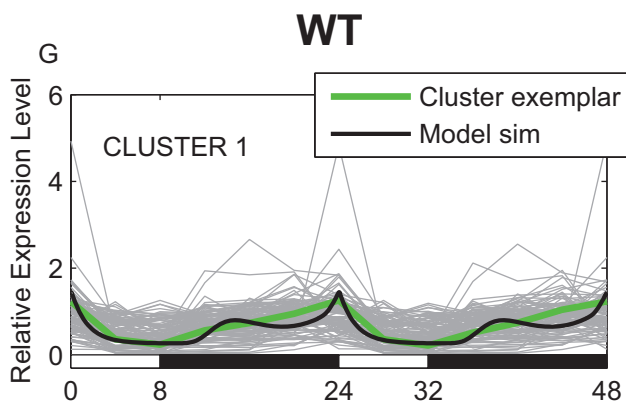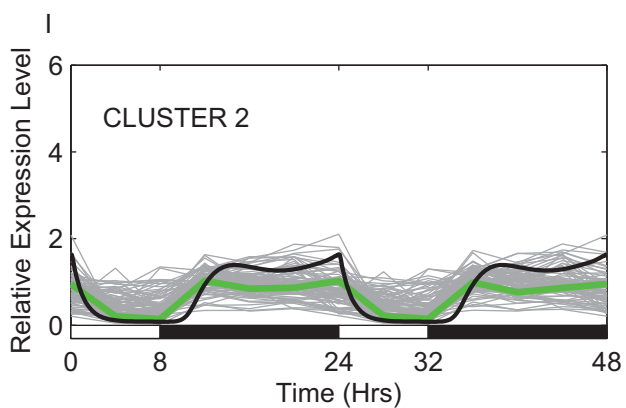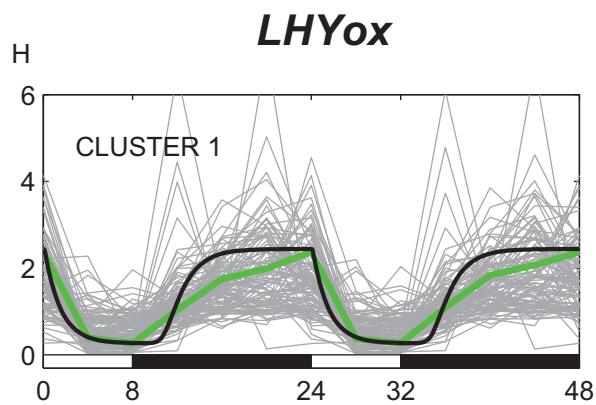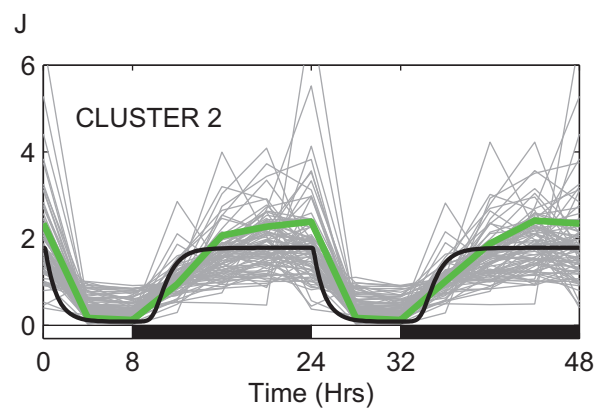

Supplement: Supplementary file 10 [file msb0011-0776-sd10.pdf]

A

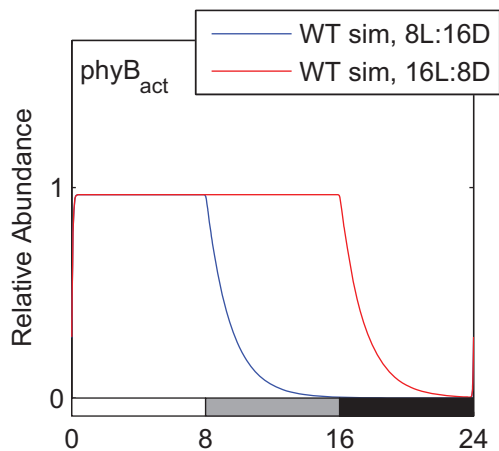

B

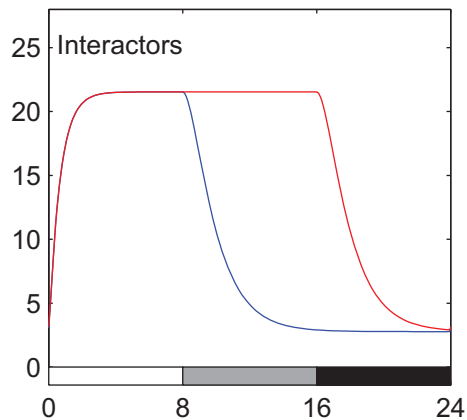

C

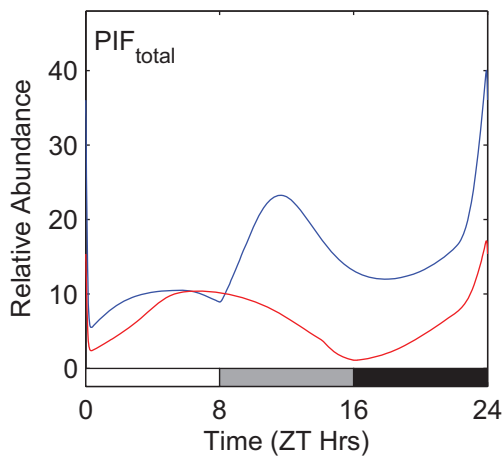

D

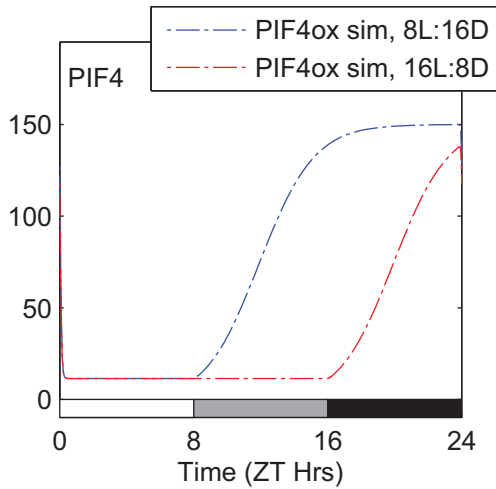

Supplement: Supplementary file 11 [file msb0011-0776-sd11.pdf]

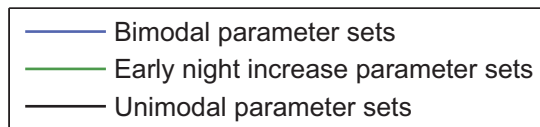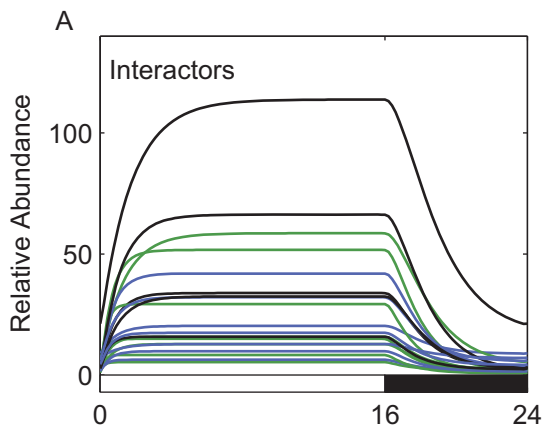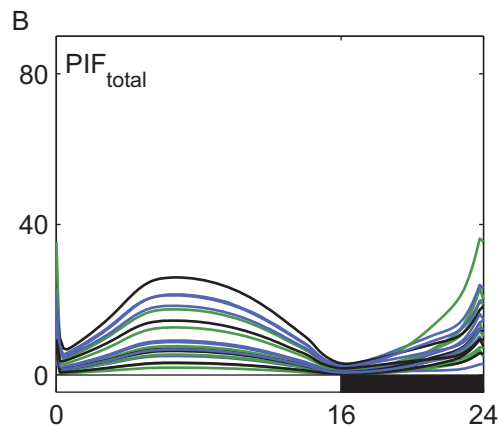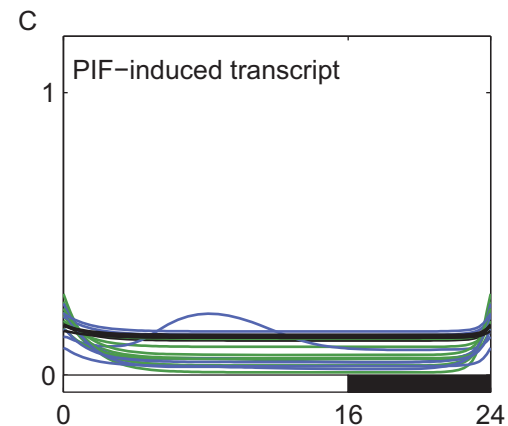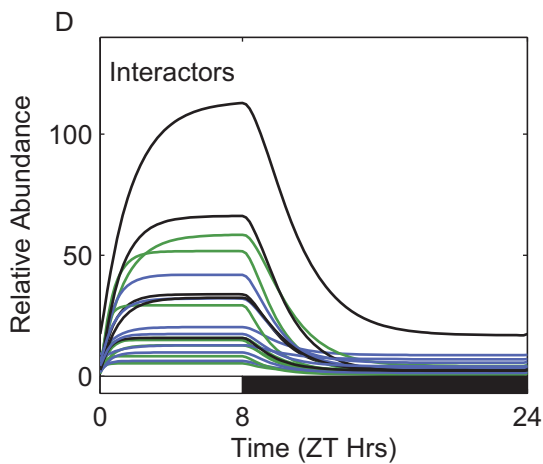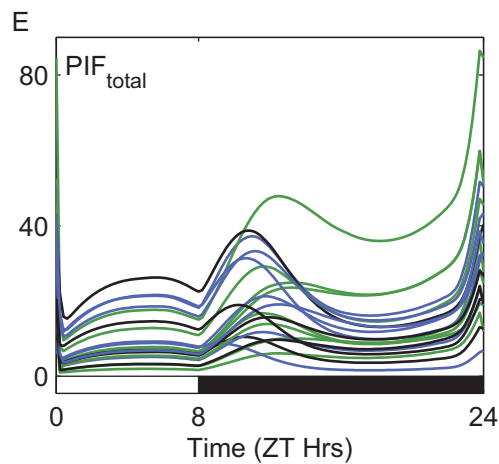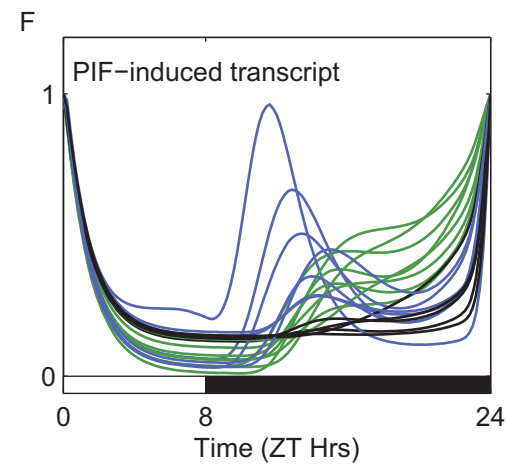

Supplement: Supplementary file 12 [file msb0011-0776-sd12.pdf]

A

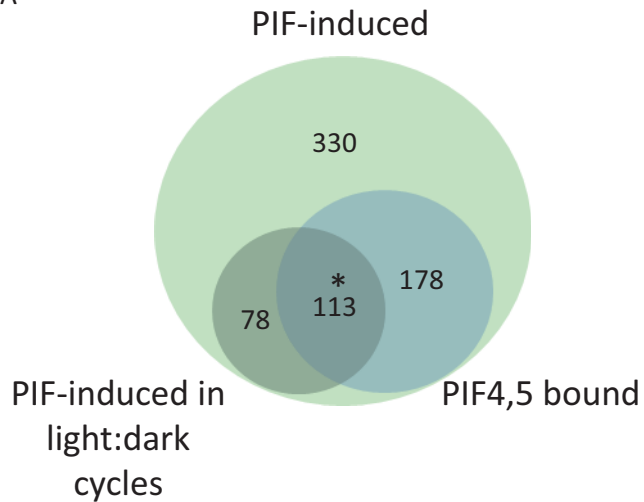

B

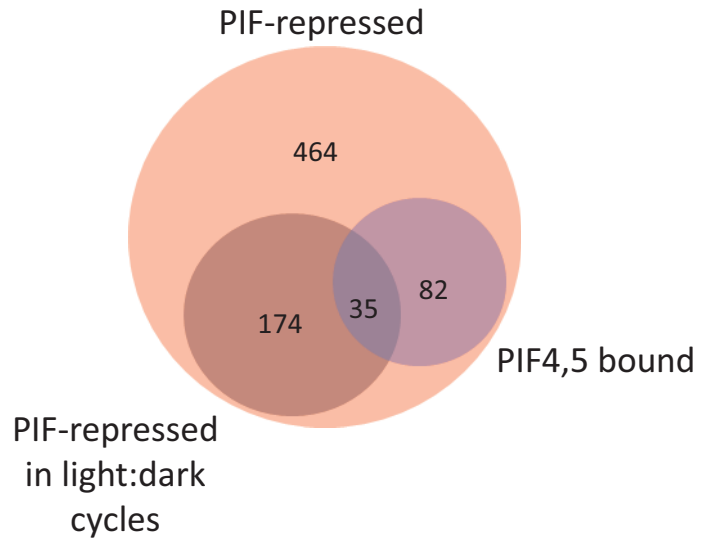

Supplement: Supplementary file 13 [file msb0011-0776-sd13.pdf]

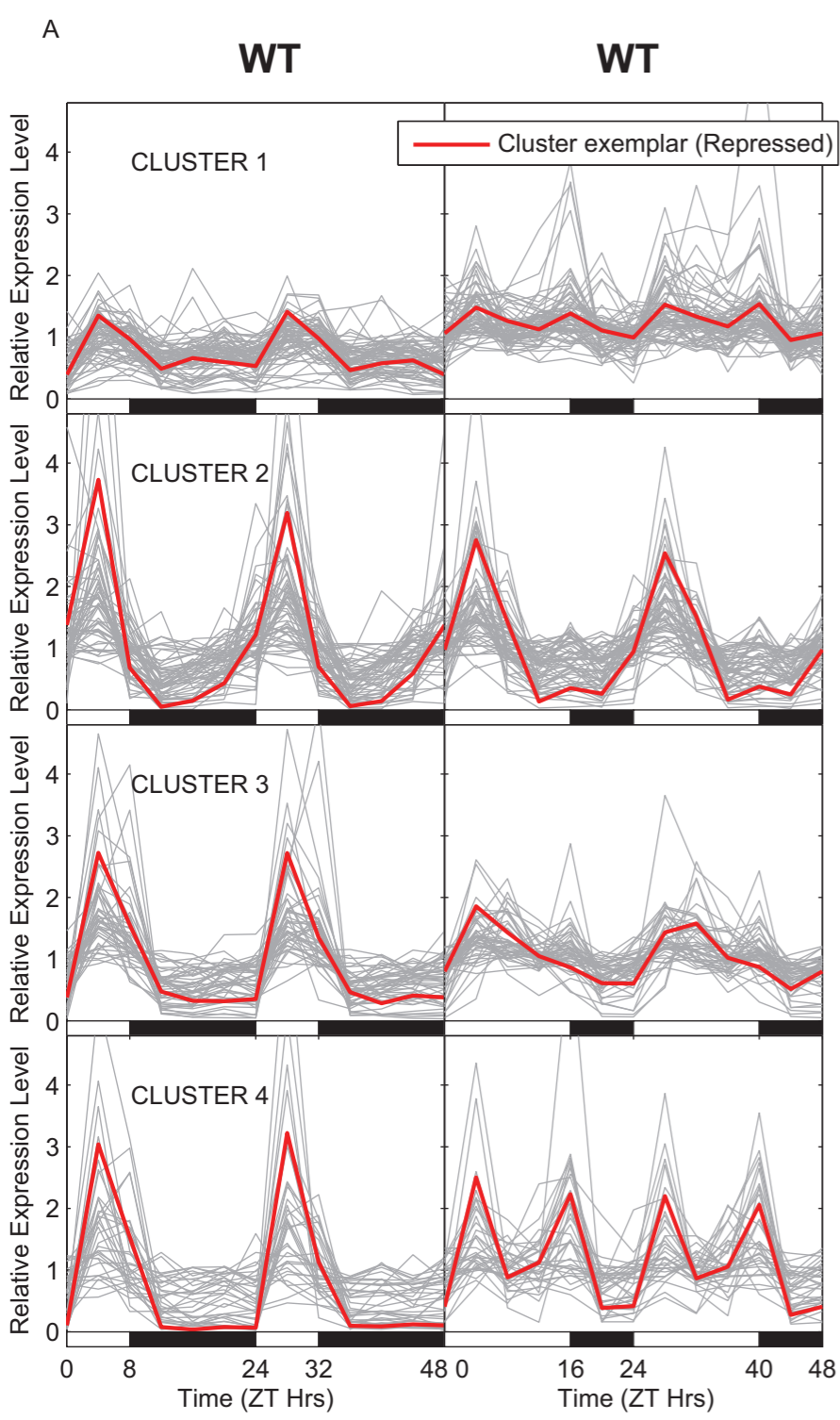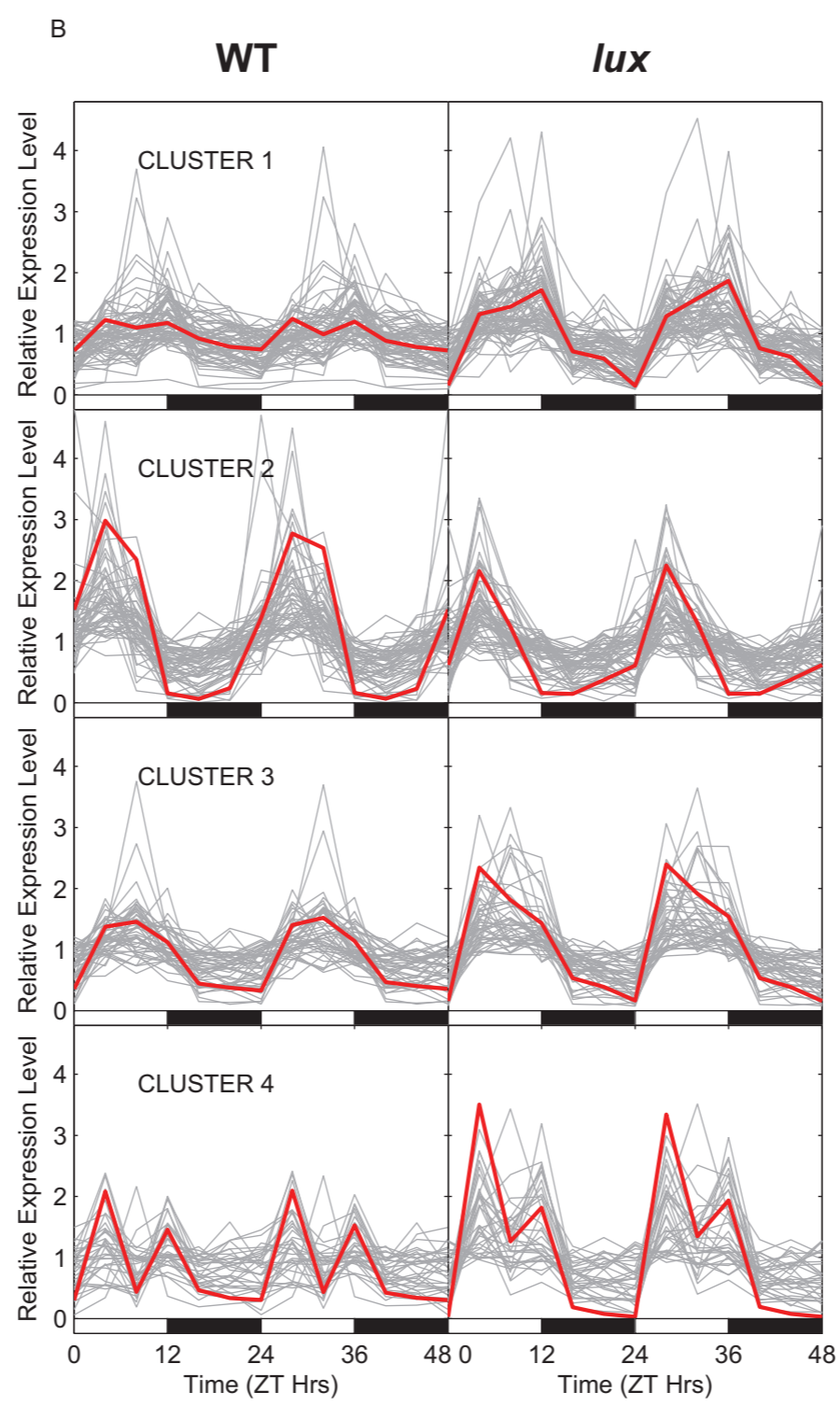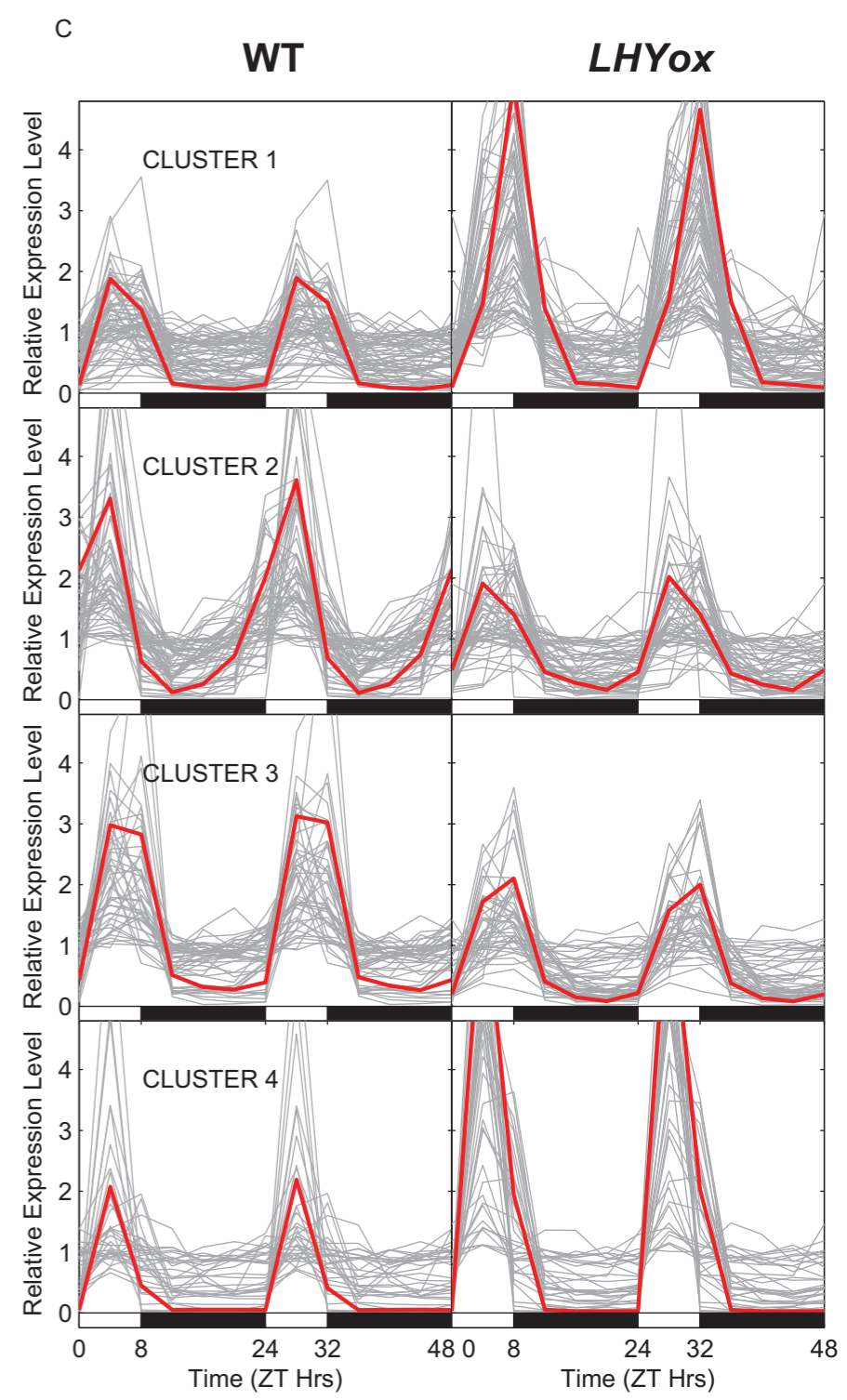

Supplement: Supplementary file 14 [file msb0011-0776-sd14.pdf]

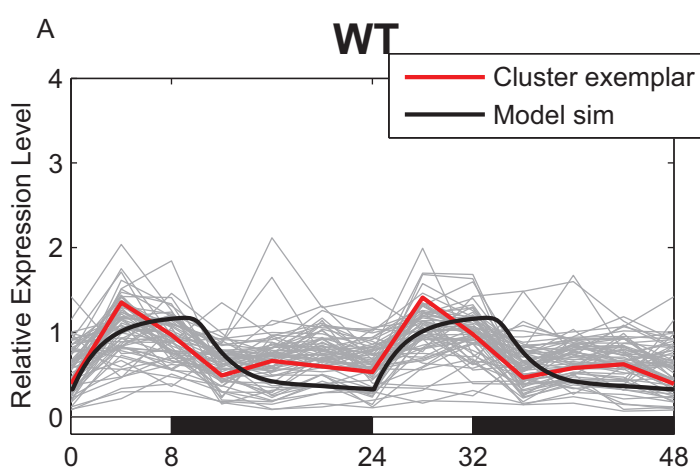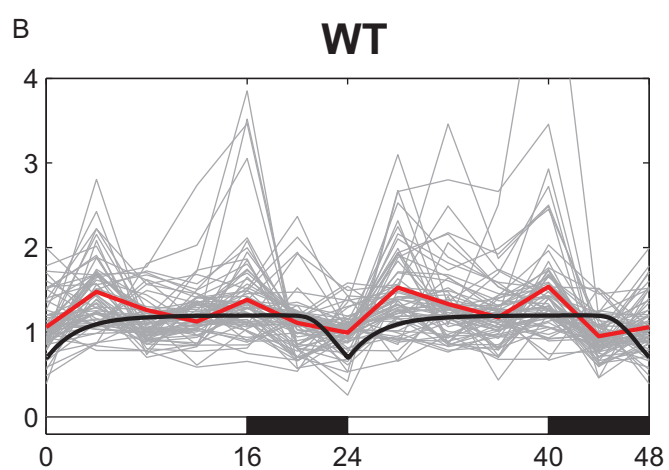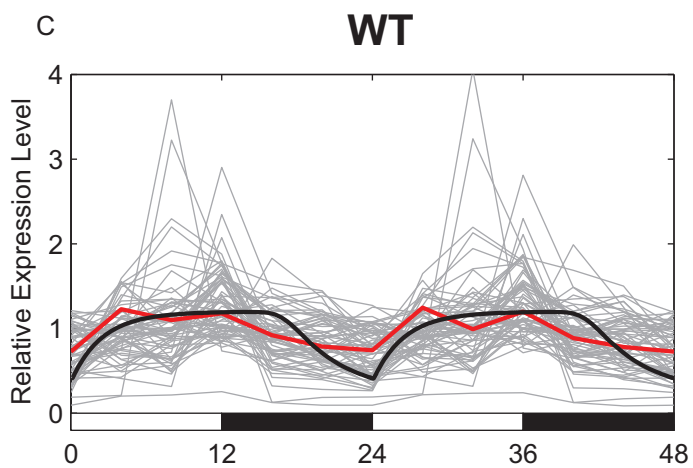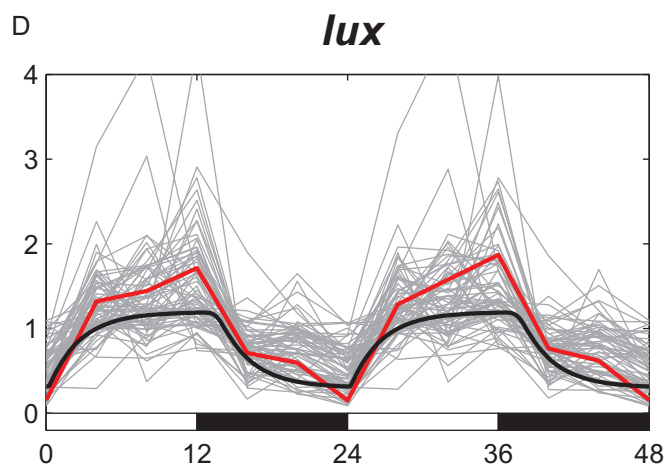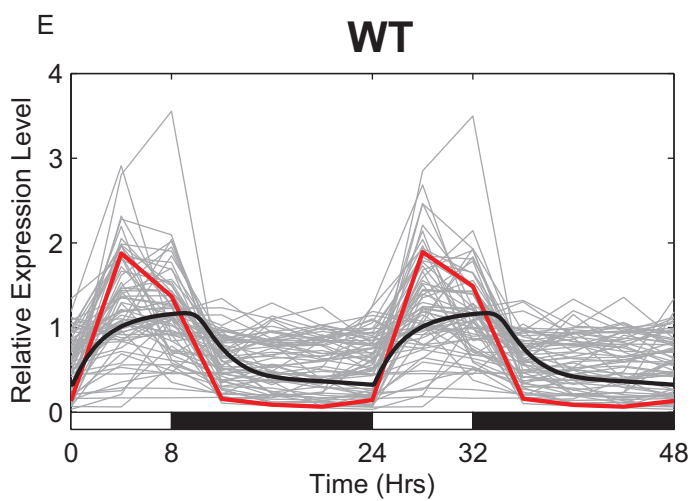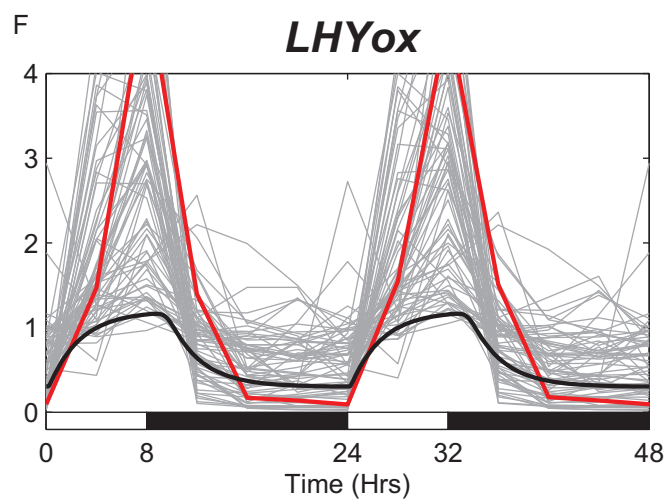

Supplement: Supplementary file 15 [file msb0011-0776-sd15.pdf]

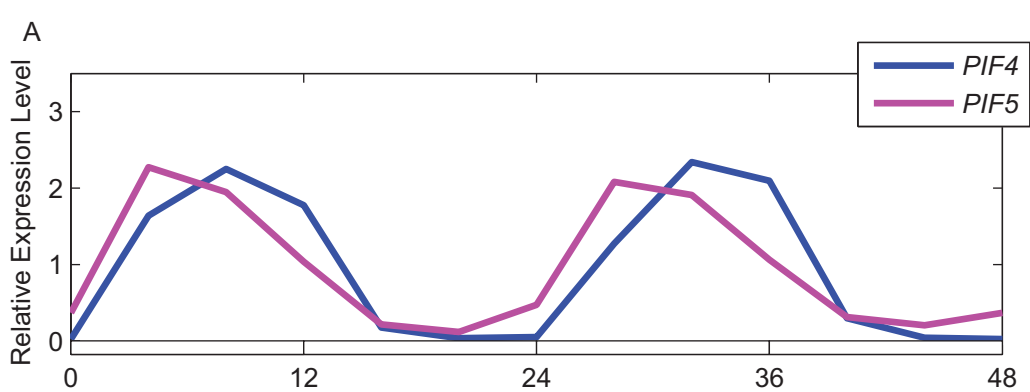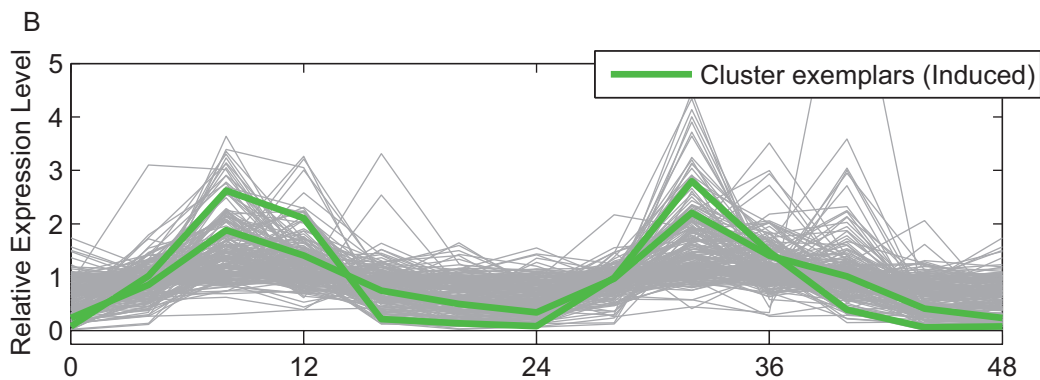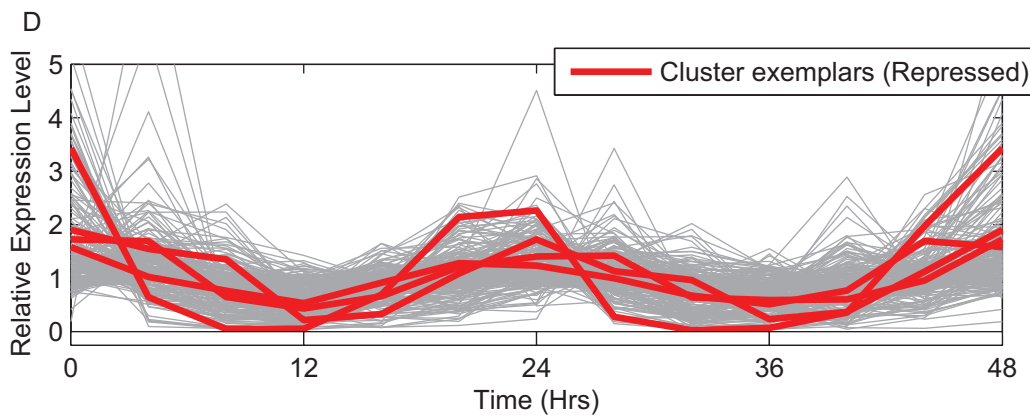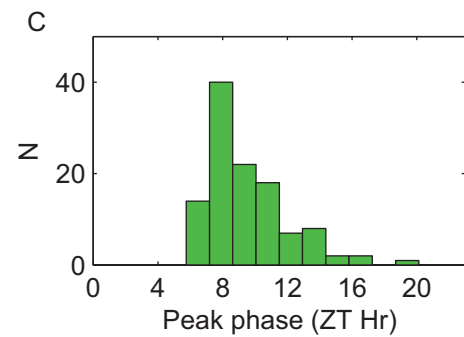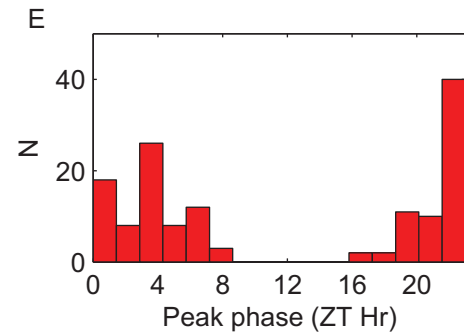

Supplement: Supplementary file 16 [file msb0011-0776-sd16.pdf]

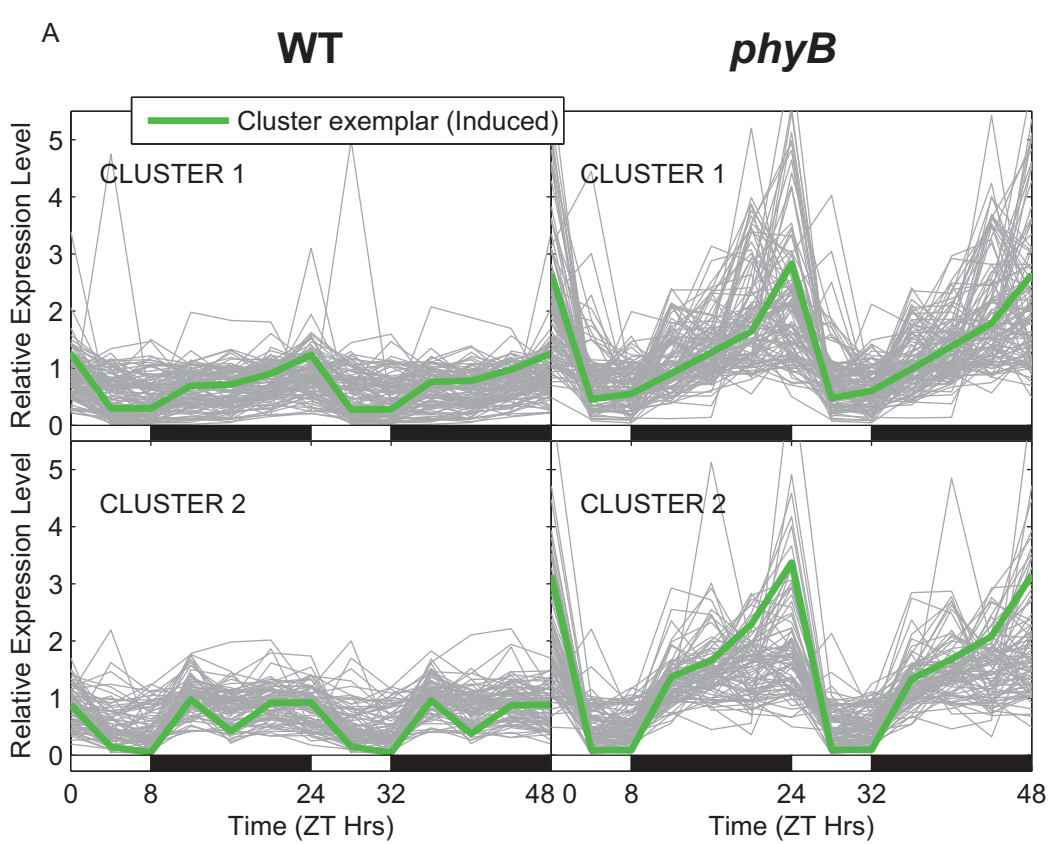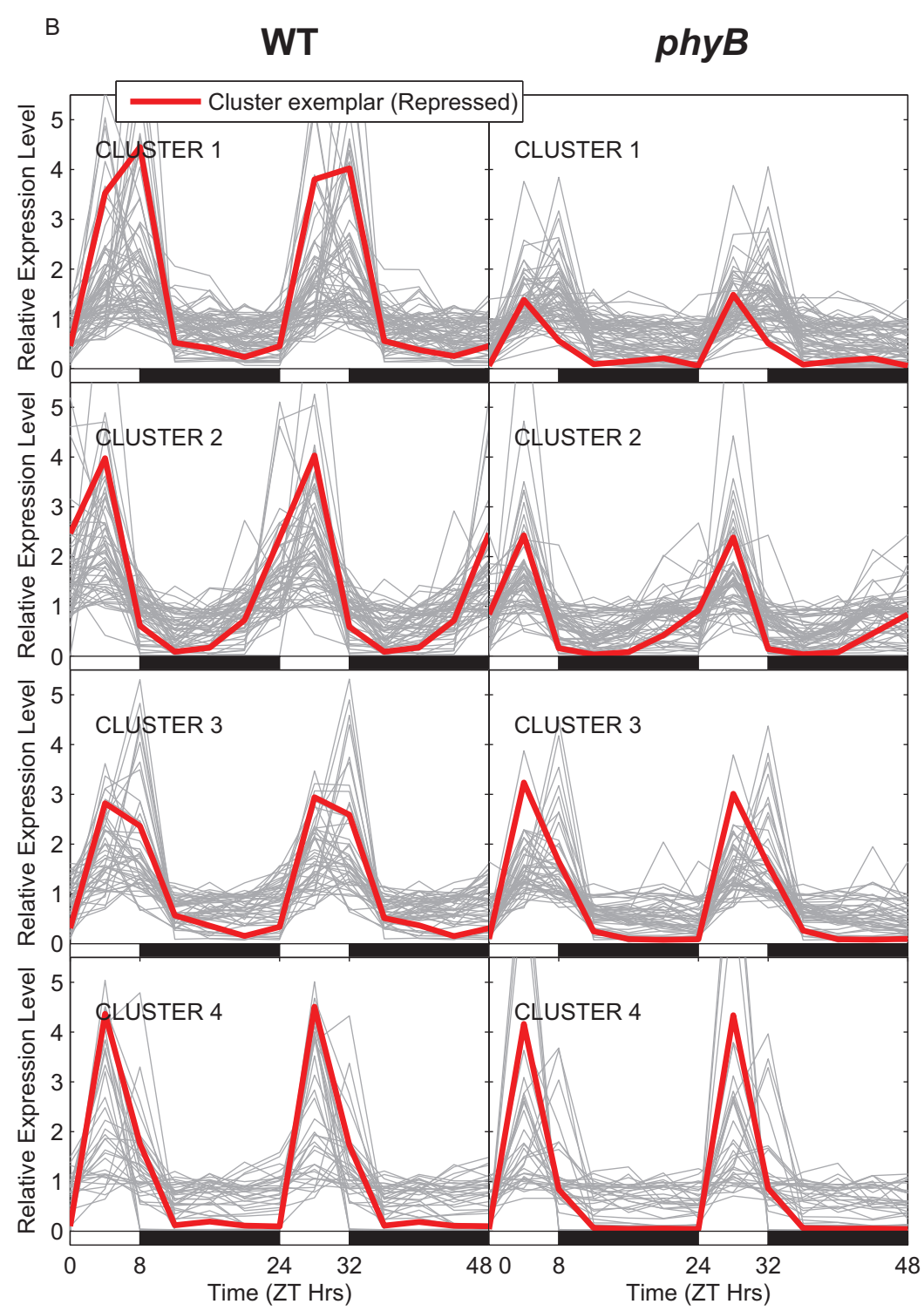

Supplement: Supplementary file 17 [file msb0011-0776-sd17.pdf]

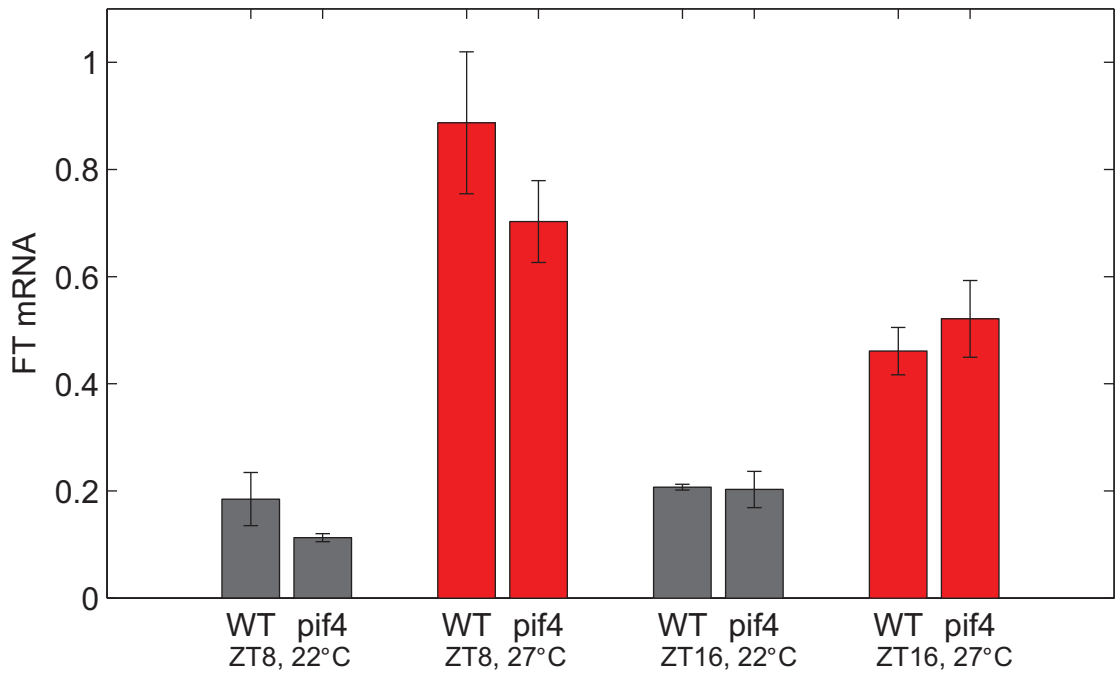

Supplement: Supplementary file 18 [file msb0011-0776-sd18.pdf]

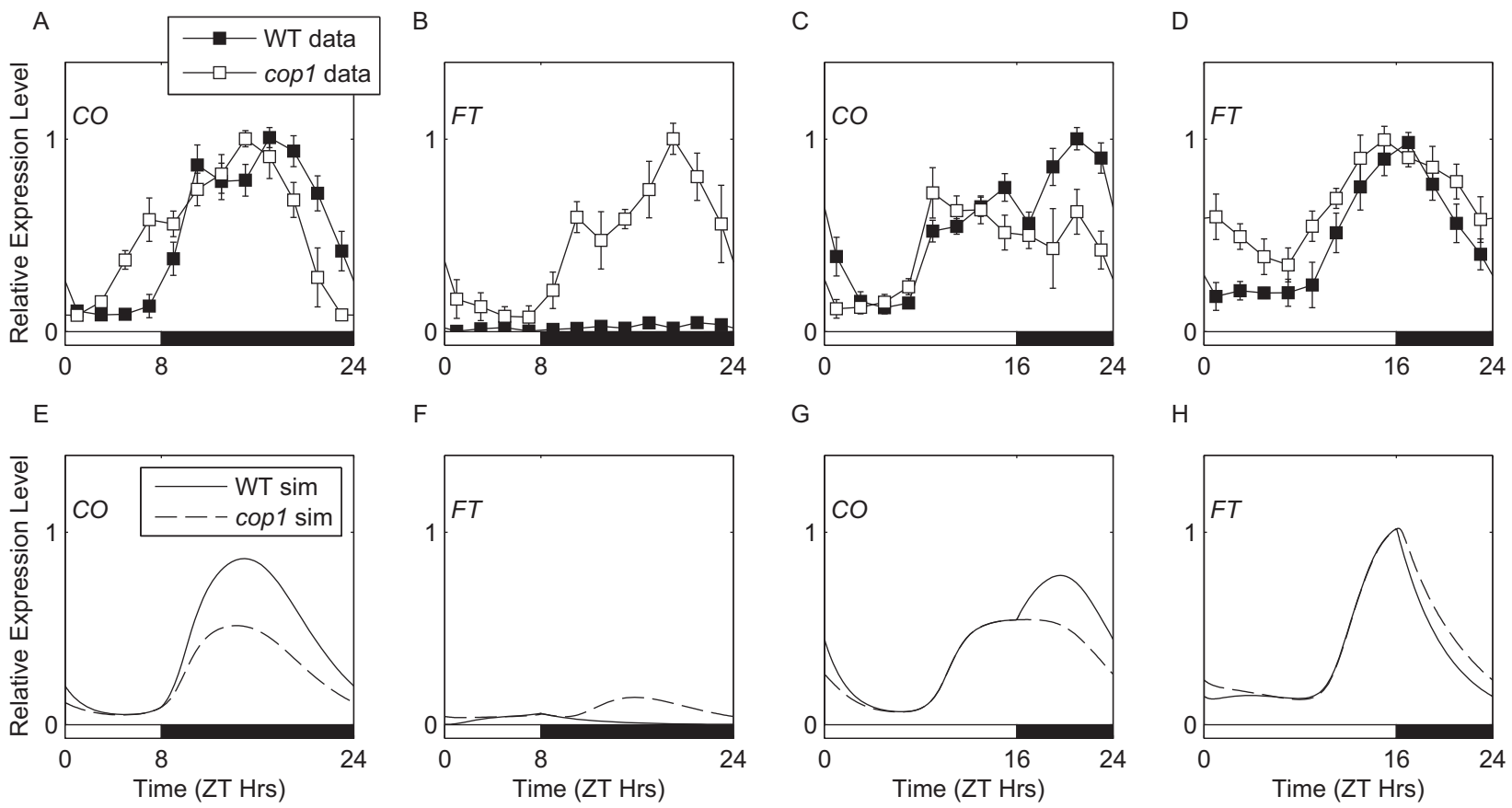

Supplement: Supplementary file 19 [file msb0011-0776-sd19.pdf]

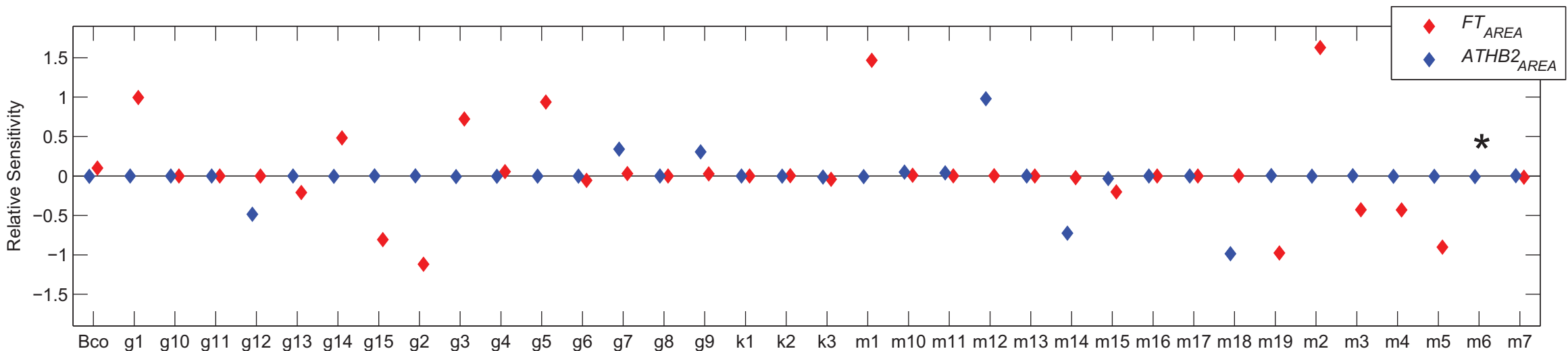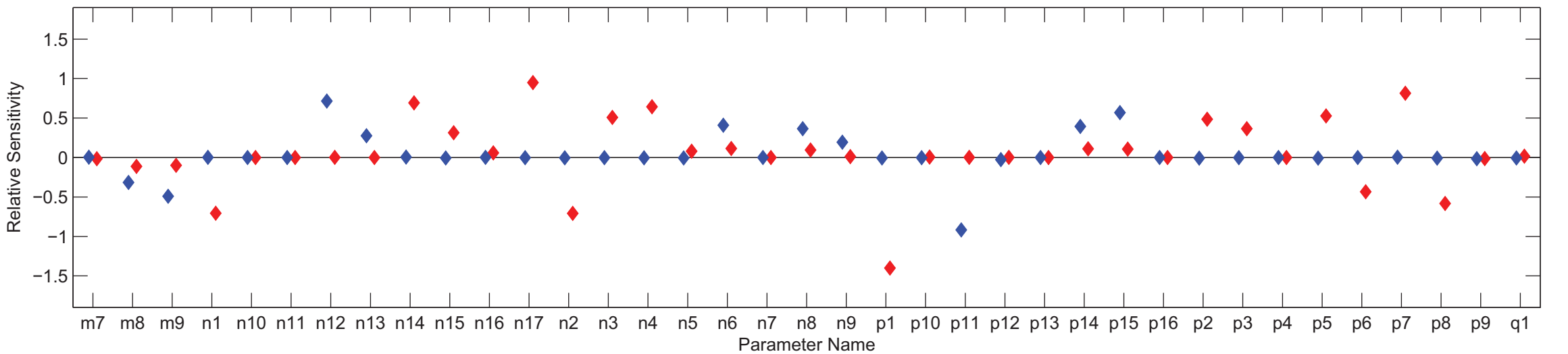

Supplement: Supplementary file 20 [file msb0011-0776-sd20.pdf]

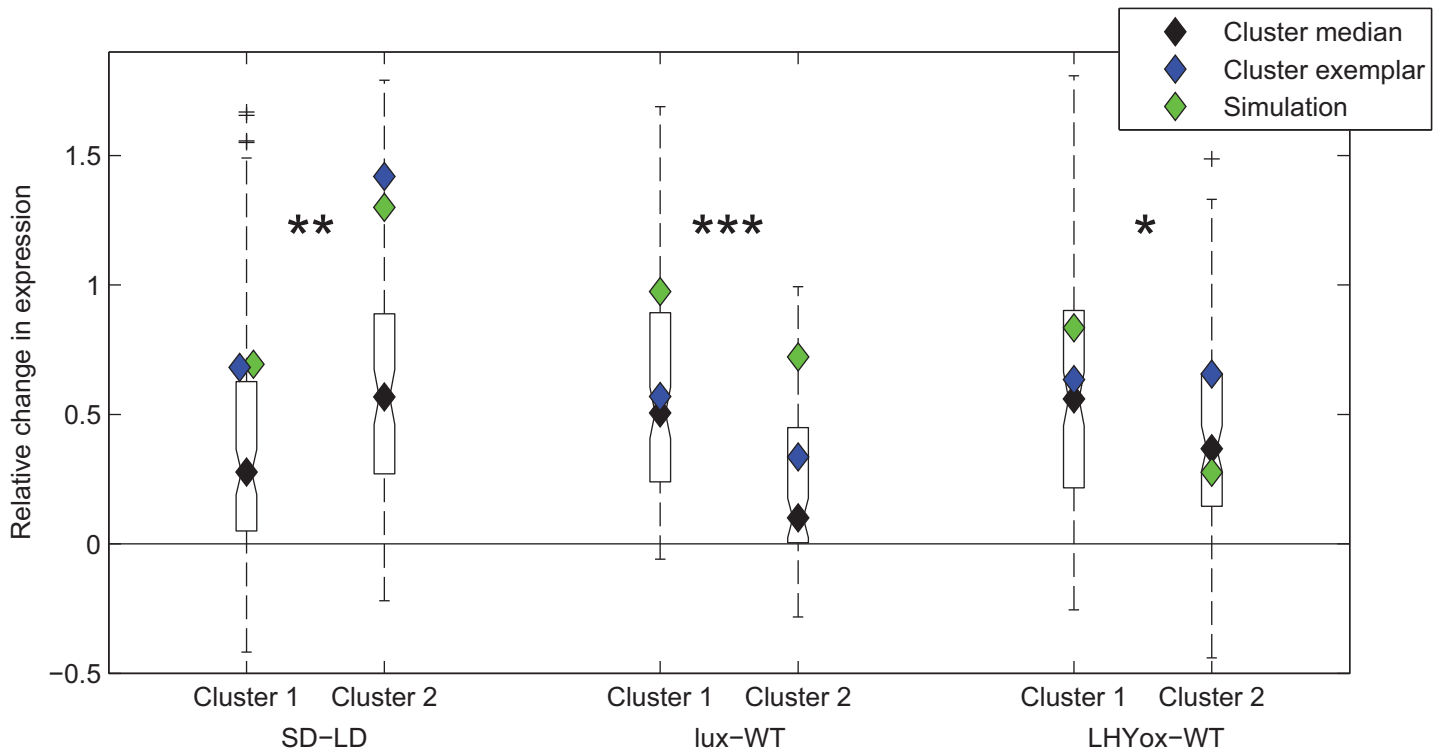

Supplement: Supplementary file 21 [file msb0011-0776-sd21.pdf]
