# Supplementary material for: Linked circadian outputs control elongation growth and flowering in response to photoperiod and temperature
Source: Mol Syst Biol. 2015 Jan 19;11(1):776. doi: 10.15252/msb.20145766 (PMC4332151; doi:10.15252/msb.20145766)
Supplement: Supplementary file 34 [file msb0011-0776-sd34.docx]

**Supplementary Information**

Table of Contents

[Computational Methods 2](#_Toc404338367)

[Parameter optimization and simulation tools 2](#_Toc404338368)

[Circadian Clock Module 2](#_Toc404338369)

[Flowering Time Module 2](#_Toc404338370)

[Hypocotyl Elongation Module 5](#_Toc404338371)

[Thermo-Photoperiodic FT Module 8](#_Toc404338372)

[Inferred hypocotyl elongation and flowering time phenotypes 9](#_Toc404338373)

[Clustering analysis of microarray data 10](#_Toc404338374)

[Model behaviour 13](#_Toc404338375)

[Circadian regulation of CO mRNA 14](#_Toc404338376)

[The model of PIF4 and PIF5 describes circadian clock mutants and multiple WT datasets 16](#_Toc404338377)

[Photoperiodic physiology 17](#_Toc404338378)

[Sensitivity analysis 18](#_Toc404338379)

[Random perturbation of parameters 19](#_Toc404338380)

[Supplementary Tables 21](#_Toc404338381)

[Supplementary Files 27](#_Toc404338382)

[Supplementary Datasets 27](#_Toc404338383)

[Supplementary Figure Legends 27](#_Toc404338384)

# Computational Methods

## Parameter optimization and simulation tools

Free parameters from the model were optimized using the simulated annealing algorithm “simulannealbnd” that is available as part of the Global Optimization Toolbox with Matlab R2013b (Mathworks, Cambridge, UK). The model was simulated using the ode15s solver and a cost function was created to minimize the Euclidean difference between the training datasets and the model simulations. All simulations were run in simulated light:dark cycles until successive days displayed identical dynamics, as done previously for the circadian clock model (Pokhilko *et al*., 2012). As discussed below, some of the parameters are fixed by the data underlying previous models. MATLAB code allowing simulation of the complete model is provided as a supplementary file. Parameter values are given in Supplementary Table 6.

## Circadian Clock Module

To model both the flowering time and hypocotyl elongation pathways, we have taken advantage of a published circadian clock model (Pokhilko *et al.*, 2012). Downstream components were added in such a manner that the clock model was unchanged from the published equations and parameter sets, similar to our earlier flowering time models (Salazar *et al.*, 2009; Song *et al.*, 2012). This modular design is intended to facilitate subsequent updating of the evolving clock model.

## Flowering Time Module

The set of equations that describe the flowering time pathway in this study are:

$\frac{dc_{CDF1}^{(m)}}{dt}=\left(n_{1}+n_{2}\frac{c_{L}^{a}}{g_{1}^{a}+c_{L}^{a}}\right)\frac{g_{2}^{b}}{g_{2}^{b}+(c_{P9}+c_{P7}+c_{NI}+c_{T})^{b}}-m_{1}c_{CDF1}^{(m)}$ (1)

$\frac{dc_{CDF1}}{dt}=p_{1}c_{CDF1}^{(m)}-m_{2}\left(1+p_{2}c_{FKF1}c_{Gn}+p_{3}c_{Gn}\right)c_{CDF1}$ (2)

$\frac{dc_{FKF1}}{dt}=p_{4}c_{FKF1}^{(m)}-p_{5}\left(m_{4}-L\frac{c_{Gn}}{k_{1}+c_{Gn}}\right)c_{FKF1}$ (3)

$\frac{dc_{FKF1}^{(m)}}{dt}=q_{1}Lc_{P}+n_{3}\frac{g_{3}^{c}}{g_{3}^{c}+c_{L}^{c}}\frac{g_{4}}{g_{4}+c_{EC}}-m_{3}c_{FKF1}^{(m)}$ (4)

$\frac{dc_{CO}^{(m)}}{dt}=B_{CO}+\frac{g_{5}^{d}}{g_{5}^{d}+c_{CDF1}^{d}}\left(n_{4}+n_{5}D\frac{c_{COP1n}}{g_{6}+c_{COP1n}}\right)-m_{5}c_{CO}^{(m)}$ (5)

$\frac{dc_{CO}}{dt}=p_{6}c_{CO}^{(m)}-p_{7}\left(m_{6}+m_{7}Dc_{COP1n}-L\frac{c_{FKF1}}{k_{2}+c_{FKF1}}\right)c_{CO}$ (6)

where components of the circadian clock have maintained the same notation as in (Pokhilko *et al.*, 2012). Here, *n*’s are transcription rates, *p*’s are translation rates, *m*’s are degradation rates, *q* describes the strength of acute light activation, *g*’s are Michaelis-Menten binding coefficients, *k*’s represents strength of protein stabilisation and *a*, *b*, *c*, and *d* are Hill coefficients. As in models of the circadian clock, *L* represents a light function and *D=1-L* represents darkness. Similar to previous flowering models, the parameter *B_CO_* is used to help simulate *CO-ox* lines (Salazar *et al.*, 2009; Song *et al.*, 2012).

Using the training datasets (see the references marked (*) Supplementary Table 4), Eqs (1-3) and (5-6) were fitted to data from WT and various mutant backgrounds. The WT and *cca1;lhy* backgrounds were used to optimise $c_{CDF1}^{(m)}$ and $c_{FKF1}^{(m)}$ in LD and SD, respectively. As in Salazar *et al.*, 2009 and Song *et al.*, 2012, $c_{CO}^{(m)}$ was fitted to WT and *fkf1* mutants in LD conditions (Supplementary Fig 5C, G). $c_{FKF1}$was fitted to WT data, but with the parameters for the FKF1-GI interaction constrained by the observation that there is FKF1 protein present in *gi-100* mutants (Fornara *et al.*, 2009). Without this constraint, the effect of GI stabilisation on FKF1 can be lost in the optimisation. As in Song *et al.*, 2012, $c_{CDF1}$ was fitted to WT and *fkf1* backgrounds (Supplementary Fig 4A). The parameters for $c_{CO}$were fitted indirectly, using the effects of genetic mutations on $c_{FT}^{(m)}$to determine CO activity, as in Song *et al.*, 2012. The parameter *q_1_* of $c_{FKF1}^{(m)}$was constrained by the observation that acute light activation of *FKF1* mRNA is weaker than that of *GI* mRNA (Tepperman *et al.*, 2004). As in the recent circadian clock model, all Hill coefficients were set to 2, representing the formation of protein dimers that regulate transcription (Pokhilko *et al.*, 2012). Thus, 25 parameters were fitted directly or indirectly to data and 5 were constrained by published observations.

The regulatory mechanisms of *CDF1* and *FKF1* mRNA modelled here are discussed in detail in the main text. Our previous model described *CO* mRNA in the *gi* mutant through GI-mediated CDF1 regulation of the *CO* expression rhythm. However, studies have observed that GI protein also plays a role in regulating CDF1 protein turnover to ensure the correct accumulation of *CO* mRNA (Imaizumi *et al.*, 2003; Imaizumi *et al.*, 2005; Sawa *et al.*, 2007; Fornara *et al.*, 2009). In order to model correctly the effect of GI on CDF1 protein, therefore, we required quantified protein profiles in WT and *gi* backgrounds, which were not previously published (although protein blot images from the *gi-2* mutant were presented in Sawa *et al.*, 2007; Figure 3B). Further, our previous flowering time models used a hypothetical component to describe the LD-specific night-time peak of *CO* mRNA (Salazar *et al.*, 2009; Song *et al.*, 2012). Here, we have also updated the flowering time module to include the COP1 component of the current circadian clock model to describe this mechanism (Eq 5; Pokhilko *et al.*, 2012). This rationale is based on evidence showing that *CO* mRNA does not obtain the LD-specific night-time peak in *cop1* mutants (Supplementary Fig 19; Yu *et al.*, 2008). COP1 also plays a second role in the system through the strong degradation of CO protein levels during the dark periods of diurnal cycles, through a direct protein-protein interaction (Fig 1, bottom inset; Jang *et al.*, 2008). Our updated model includes this interaction to describe the dark-dependent CO degradation (Eq 6). The result of this interaction is that in *cop1* mutants, CO protein is present during the dark and, as a consequence, *FT* mRNA is able to accumulate, particularly in SD (Supplementary Fig 19).

## Hypocotyl Elongation Module

The set of model equations that describe the photoperiod-dependent pathway of hypocotyl elongation are (8 variables, 4 fixed parameters (PhyB and PR), 22 optimised parameters):

$\frac{dc_{PIF4}^{(m)}}{dt}=n_{6}\frac{g_{7}}{g_{7}+c_{EC}(\tau)}+n_{7}-m_{8}c_{PIF4}^{(m)}$ (7)

$\frac{dc_{PIF4}^{(m)}}{dt}=n_{8}\frac{g_{8}}{g_{8}+c_{EC}(\tau)}+n_{9}-m_{9}c_{PIF5}^{(m)}$ (8)

$\frac{dc_{phyB}}{dt}=p_{8}L(1-c_{phyB})-m_{10}c_{phyB}$ (9)

$\frac{dc_{PR}}{dt}=p_{9}L(1-c_{PR})-m_{11}c_{PR}$ (10)

$\frac{dc_{Int}}{dt}=p_{10}+p_{11}c_{PR}-m_{12}c_{Int}$ (11)

$\frac{dc_{PIF}}{dt}=\left( p_{12}c_{PIF4}^{\left( m \right)}+p_{13}c_{PIF5}^{\left( m \right)} \right)-(m_{13}+m_{14}c_{phyB})c_{PIF}$ (12)

$\frac{dc_{IAA29}^{(m)}}{dt}=n_{10}+n_{11}\frac{{c_{{PIF}_{act,IAA29}}}^{2}}{{g_{9}}^{2}+{c_{{PIF}_{act,IAA29}}}^{2}}-m_{15}c_{IAA29}^{(m)}$ (13)

$\frac{dc_{ATHB2}^{(m)}}{dt}=n_{12}+n_{13}\frac{{c_{{PIF}_{act,ATHB2}}}^{2}}{{g_{10}}^{2}+{c_{{PIF}_{act,ATHB2}}}^{2}}-m_{16}c_{ATHB2}^{(m)}$ (14)

$c_{{PIF}_{act,IAA29}}=\frac{c_{PIF}^{2}}{c_{PIF}^{2}+{(g_{14}c_{Int})}^{2}}$ (15)

$c_{{PIF}_{act,ATHB2}}=\frac{c_{PIF}^{2}}{c_{PIF}^{2}+{(g_{15}c_{Int})}^{2}}$ (16)

In addition, the additional model outputs were introduced and fitted to microarray timeseries data for cluster exemplars (where $c_{Induced, C1}^{(m)}$, $c_{Induced, C2}^{(m)}$, and $c_{Repressed, C1}^{(m)}$ represent the exemplars of Induced Cluster 1, Induced Cluster 2, and Repressed Cluster 1, respectively):

$\frac{dc_{Induced, C1}^{(m)}}{dt}=n_{18}+n_{19}\frac{{c_{{PIF}_{act,Induced,C1}}}^{2}}{{g_{19}}^{2}+{c_{{PIF}_{act,Induced,C1}}}^{2}}-m_{18}c_{Induced,C1}^{(m)}$ (17)

$\frac{dc_{Induced, C2}^{\left( m \right)}}{dt}=n_{20}+n_{21}\frac{{c_{{PIF}_{act,Induced,C2}}}^{2}}{{g_{20}}^{2}+{c_{{PIF}_{act,Induced,C2}}}^{2}}-m_{19}c_{Induced, C2}^{\left( m \right)}$ (18)

$\frac{dc_{Repressed, C1}^{(m)}}{dt}=n_{22}+n_{23}\frac{{g_{21}}^{2}}{{g_{21}}^{2}+{c_{{PIF}_{act,Repressed,C1}}}^{2}}-m_{20}c_{Repressed,C1}^{(m)}$ (19)

$c_{{PIF}_{act,Induced,C1}}=\frac{c_{PIF}^{2}}{c_{PIF}^{2}+{(g_{16}c_{Int})}^{2}}$ (20)

$c_{{PIF}_{act,Induced,C2}}=\frac{c_{PIF}^{2}}{c_{PIF}^{2}+{(g_{17}c_{Int})}^{2}}$ (21)

$c_{{PIF}_{act,Repressed,C1}}=\frac{c_{PIF}^{2}}{c_{PIF}^{2}+{(g_{18}c_{Int})}^{2}}$ (22)

Parameter names maintain the same notation as in the ‘Circadian Clock Module’ and ‘Flowering Module’ (see above), and circadian clock components take on the same notation as the clock model. In this system, the component ‘Int’ represents the ‘Interactor’ proteins shown in Fig 1 (bottom inset), and phyB & PR represent the *Arabidopsis thaliana* photoreceptors. In particular, PR represents a ‘generic photoreceptor’, as the ‘Interactor’ proteins constitute a class of proteins that are stimulated through multiple different light-sensitive pathways.

The parameter τ represents the time-advance of the EC profile from the ‘Circadian Clock Module’ required for an accurate description of *PIF4* and *PIF5* mRNA profiles. By advancing the EC dynamics to provide an accurate description of *PIF4* and *PIF5* mRNA, the simulated EC profile provided a closer match to the data than was required for an accurate description of core clock components (see the main text; Nusinow *et al.*, 2011). The parameters for *PIF4* and *PIF5* mRNA were each optimised to three datasets: WT data from three photoperiods (8L:16D, 12L:12D and 16L:8D diurnal cycles) taken from Nusinow *et al.*, 2011. Since there is little data on dynamic changes in PIF protein levels across diurnal cycles, the PIF protein parameters were optimised indirectly through the optimisation of *ATHB2* and *IAA29* mRNA profiles in 8L:16D and 16L:8D cycles (Fig 4C,D,F,G; Nomoto *et al*., 2012b). These equations were further constrained by the non-linear relationship observed between photoperiod and *ATHB2* expression at dawn (see Fig 4H; Kunihiro *et al.*, 2011). Finally, the effects of temperature on *PIF4* transcription are represented by a reduction in the strength of *PIF4* repression by the EC by multiplying the parameter *g7* by a factor of 4 at the higher temperature (i.e. 27°C), on the basis of comparing simulations and *PIF4* and *ATHB2* data from the two temperatures (see Fig 6B-E).

Since little is known about differences in the regulation of PIF4 and PIF5, we treated both identically at the post-translational level, with PIF4 and PIF5 represented together as ‘PIF’ protein. No attempt is made to distinguish between the relative contributions of the various types of PIF homo- and hetero-dimers, as they are highly redundant and no suitable data are available. It has been well established that degradation of PIF4 and PIF5 is stimulated by their interaction with the light-activated form of phyB (Al-Sady *et al.*, 2008). In order to model this in a simple way, it is assumed that there is a constant level of phyB that is activated in a reversible manner by light (Eq 9). This is represented by the multiplication of *p_9_* by L. The rate of dark reversion (that provides a time-scale of inactivation) of phyB is represented by the parameter *m_10_*. This model is a simplified representation of the model published in Rausenberger *et al.*, 2010. The additional complexity present in the model of Rausenberger *et al.*, 2010 describes the dependence of phyB activation on light intensity and wavelength, which is not of immediate interest here. The degradation of PIF proteins depends on phyB-independent and phyB-dependent rates (*m_14_* and *m_15_*, respectively). As well as light-modulated PIF protein degradation, PIF proteins have been shown to have numerous interacting partners that can sequester PIF activity (termed ‘Interactors’ in Fig 1A, bottom inset; de Lucas *et al.*, 2008; Foreman *et al.*, 2011; Hao *et al.*, 2012; Hornitschek *et al.*, 2009; Bai *et al.*, 2012; Oh *et al.*, 2012). This is represented by a pool of ‘Interactor’ proteins (see below for a discussion on the possible identity of these). The synthesis of the ‘Interactor’ proteins has a basal level (given by *p_11_*) and is stimulated by a generic photoreceptor PR (with the magnitude of stimulation given by the parameter *p_12_*). Since PR is proposed to act in a similar manner to phyB, albeit independently (possibly through phyB-independent complexes featuring the other red-light activated phytochromes phyC/D/E; Clack *et al.*, 2009), the parameters *p_9_* = *p_10_* and *m_10_* = *m_11_*. The effect of ‘Interactor’ repression of PIF activity are assumed to depend on the PIF target, with the parameters *g_13_* and *g_14_* representing the strength of ‘Interactor’ repression of PIF activity towards *ATHB2* and *IAA29*, respectively. The nonlinear dependence of PIF activity on PIF protein levels is based on the observed nonlinear switching behaviour arising from networks of competing protein-protein interactions (Buchler and Cross, 2009). In total, 26 parameters were optimised to match available data, and 4 parameters (describing the dynamics of photoreceptor activation and dark reversion) were fixed on the basis of prior knowledge. In addition, each cluster exemplar was modelled by setting 5 parameters to fit measured dynamics (other parameters remained the same).

## Thermo-Photoperiodic FT Module

Recent experimental studies have shown that active PIF protein complexes are key regulators of the accelerated flowering phenotypes seen in warmer ambient temperatures (27°C) compared to cooler temperatures (22°C, see main text; Kumar *et al.*, 2012). The current hypothesis proposed by these studies is that PIFs (and therefore temperature) accelerate flowering independently of CO, but in a manner dependent on *FT*. Our data show that PIF proteins regulate *FT* mRNA at least partly through a light-dependent process, which we hypothesise may involve a CO-PIF interaction. This led to our model equation for *FT* mRNA taking the form:

$\frac{dc_{FT}^{(m)}}{dt}=\left(n_{14}+n_{15}\frac{c_{PIF}}{g_{11}+c_{PIF}}\right)\left(n_{16}+n_{17}\frac{g_{12}}{g_{12}+c_{CDF1}}\right)\frac{c_{CO}^{h}}{g_{13}^{h}+c_{CO}^{h}}-m_{19}c_{FT}^{(m)}$ (23)

where parameters are labelled as in the other modules described above. As in Song *et al.*, 2012, Eq (23) was fitted primarily to data from WT and *fkf1* backgrounds in LD (Supplementary Fig 5). Since the activity of PIF towards *FT* appears to be regulated in a distinct way to PIF activity towards *ATHB2* and *IAA29* (see Main Text), the role of ‘Interactors’ in the regulation of PIF activity towards *FT* is left out. To optimise the contribution of PIF to the regulation of *FT* mRNA, we additionally fitted the parameters *n_14_*, *n_15_* and *g_11_* to *pif4;5* data in 22°C LD conditions (Fig 6H,I). To simulate 27°C conditions, *FT* transcription by PIF-dependent and PIF-independent regulation (i.e. the parameters *n_14_* and *n_15_*) were both increased by a factor of 3.24.

## Inferred hypocotyl elongation and flowering time phenotypes

As with previous flowering time models, and due to the lack of knowledge detailing mechanistic processes further downstream in the pathways modelled here, we have used *ATHB2* and *FT* mRNA accumulation as molecular readouts for the overt hypocotyl length and flowering time phenotypes, respectively (Salazar *et al.*, 2009; Kunihiro *et al.*, 2011). By calculating the area under the curve of the respective expression levels (denoted *ATHB2_AREA_* and *FT_AREA_*) we are able to compare the effects of different photoperiods and mutations on the hypocotyl and flowering pathways.

In Fig 5C,D, we used two approximate functions to relate *ATHB2* and *FT* expression to the hypocotyl length and flowering time of WT plants across different photoperiods. The function used to calculate hypocotyl elongation is:

| $Hyp\_Length=a_{1}\int_{0}^{24} (z(t)-a_{2})$ | (24) |
| --- | --- |

where *z(t)* = $c_{ATHB2}^{(m)}$, if $c_{ATHB2}^{(m)}<a_{3}$, and *z(t)* = *a_3_*, if $c_{ATHB2}^{(m)}\geq a_{3}$, for *t* = [0,24]. Thus *a_3_* represents a saturation term whereby increased *ATHB2* mRNA expression does not lead to further regulation of downstream processes. The function used to calculate flowering time is:

| $Days\_to\_Flower=d0+\frac{a_{4}}{1-FT_{AREA}/a_{5}}$ | (25) |
| --- | --- |

where *FT_AREA_* is the area under the curve of *FT* mRNA expression in one diurnal cycle (as calculated in Salazar *et al.*, 2009). The parameter *d0* is the same value as in Salazar *et al.*, 2009, whilst parameters *a_1_*, *a_2_*, *a_3_, a_4_* and *a_5_* were optimised by comparing the functions to hypocotyl length and flowering time data from across different photoperiods (Corbesier *et al.*, 1996; Kunihiro *et al.*, 2011).

## Clustering analysis of microarray data

Lists of transcripts identified as PIF-induced (i.e. having reduced levels in the *pif1/4/5* mutant) and PIF-repressed (i.e. those having increased levels in the *pif1/4/5* mutant) were taken from (Zhang *et al.*, 2013). Microarray timeseries data for three comparable pairs of conditions (i.e. six datasets in total) were downloaded from the DIURNAL database (Mockler *et al*., 2007). The three comparable sets were SD/LD (from Michael *et al*., 2012b), WT/*lux*, and WT/*LHYox* (both from Michael *et al*., 2012a). Clustering was performed by affinity propagation, which takes as input a similarity metric between datapoints (in this case, genes) and identifies subsets of representative examples of typical behaviour (i.e. cluster exemplars) (Frey and Dueck, 2007). The ‘preference’ parameter (a weighting parameter determining how likely a given gene is to be assigned as a cluster exemplar) was set to -7, and the ‘damping’ parameter was set to 0.5.

An appropriate similarity metric for clustering timeseries data is the Spearmans’s correlation coefficient. This is suitable for capturing correlations between microarray timeseries data within a particular experiment, where data are quantitatively comparable. We therefore separated the microarray timeseries into comparable sets, as in (Michael *et al*., 2012a; Michael *et al*., 2012b), and took the sum of Spearman’s correlation coefficients as the similarity metric. In particular, the similarity metric between two transcripts $i$ and $j$ in the experiment is given by:

$s\left( i,j \right)=\sum_{c\epsilon C} \rho(x_{i}^{c},x_{j}^{c})$ (26)

Where $s(i,j)$ denotes the similarity of transcripts $i$ and $j$, $\rho$ denotes the Spearman’s correlation coefficient function, and $x_{i}^{c}$ denotes the dynamics of transcript $i$ in the pair of conditions $c$, with $c$ taken from the set C = {‘SD/LD’,’WT/*lux*’,’WT/*LHYox*’}. Timeseries data from comparable pairs of experiments were concatenated before calculating the similarity metric. For example:

$x_{i}^{SD/LD}=(x_{i}^{SD},x_{i}^{LD})$ (27)

This allowed the clustering to take into account quantitative changes in dynamics across conditions (e.g. the relative magnitude of peak expression).

The clusters of genes that were identified as displaying dynamics consistent with regulation (either induction or repression) by PIFs in light:dark cycles were also selected based on their high intracluster coherence, as measured by the mean similarity of a cluster member to its cluster exemplar (and therefore spanning a range between -3 and 3). In particular, for PIF-induced genes, the intracluster coherence of Clusters 1 and 2 were both >2.17, higher than any other clusters in this set (11 clusters total, median intracluster coherence = 1.58). For PIF-repressed genes, the intracluster coherence of Clusters 1, 2, 3, and 4 were all >2.05, and were all higher than all but one cluster in this set (13 clusters total, median intracluster coherence = 1.90).

Microarray data for the comparison of WT with the *phyB* mutant in 8L:16D light:dark cycles (Michael *et al*., 2008a; Supplementary Fig 17) was not incorporated into the similarity metric, meaning that the cluster exemplars in this case are not necessarily representative of the behaviour of transcripts within their clusters. Nevertheless, the median Spearman’s correlation coefficient between transcripts and their cluster exemplar was >0.71 for all 6 clusters (i.e. 2 PIF-induced and 4 PIF-repressed) identified as displaying dynamics consistent with PIF regulation in light:dark cycles (shown in Supplementary Fig 17). This demonstrates that the clustered genes are also similarly regulated in these conditions. Similarly, microarray data from plants grown in continuous light under temperature cycles (Michael *et al*., 2008b; Supplementary Fig 16) was not used for to cluster genes. The coherent behaviour of clusters in this condition is demonstrated by the similar peak phase of genes within the PIF-induced and PIF-repressed sets (with peak phase defined as given in the DIURNAL database (Mockler *et al*., 2007), with a self-correlation cut-off of 0.8 applied to filter out arrhythmic transcripts).

In order to plot transcript dynamics (Fig 4, Supplementary Figs 9, 10, 14, 15, 16 & 17), the expression of each transcript was normalised by dividing by its average level across both conditions in each pair of conditions.

# Model behaviour

The model described above simulates the photoperiodic regulation of the flowering-time pathway based upon rhythmic regulation from the circadian clock model presented in (Pokhilko *et al.*, 2012), without the input timeseries of *FKF1* and *CDF1* data that our previous flowering model required (compare the top and bottom insets of Fig 1; Song  *et al.*, 2012). The present model can therefore be simulated in conditions where input data are not yet available but also, importantly, the present model preserves the behaviours previously matched to experimental data (Supplementary Fig 8; Song *et al.*, 2012). Simulated *fkf1* mutants under LD conditions lose the shoulder of *CO* mRNA expression during the afternoon (~ZT10-16, Fig 3E and Supplementary Fig 5G). Simulated *gi* mutants have a significantly dampened amplitude and mean level of rhythmic *CO* mRNA expression (Fig 3E and Supplementary Fig 6E,G). There was very little simulated *FT* mRNA expression in either of these mutant backgrounds (Supplementary Figs 5F,H & 6F,H). As with our previously published model, the new model matched the effects on *FT* transcription rhythms in *CO-ox;fkf1* and *CO-ox;CDF1-ox* backgrounds. Here, overexpression of *CDF1* had a stronger suppressive effect on the *FT* mRNA rhythm in *CO-ox* than the loss of *FKF1* mRNA expression (Supplementary Fig 8A; Song *et al.*, 2012). The relative effects of the two roles of FKF1 protein in the flowering system, namely the stabilisation of CO protein and the regulation of *CO* mRNA through CDF1, were also quantified. As with our previous model, the present model predicts that removal of the FKF1-CO interaction has a larger effect on *FT* expression than the removal of the FKF1-CDF1 interaction (Supplementary Fig 8B; Song *et al.*, 2012). Thus the new connections that confer rhythmic regulation to *FKF1* and *CDF1* mRNA from the clock model match the observed dynamics of these mRNAs (Fig 2C,D) and also maintain the key model behaviours, as expected.

## Circadian regulation of CO mRNA

In the main text we discuss regulation of *CO* mRNA in the *prr9;7* double mutant. To show that the flowering time module is also able to simulate further mutants we have tested the *cca1;lhy, cop1* and *elf3* mutants (Fig 3G and Supplementary Figs 1E, 2E and 5E & G). Below, we discuss in more detail the regulation of flowering by CCA1/LHY and COP1.

In previous mathematical models of the flowering network, dark-dependent increases in *CO* mRNA levels were simulated using a hypothetical night-time activator acting independently of the photoperiodic pathway (Salazar *et al.*, 2009; Song *et al.*, 2012). However, published evidence supports a mechanism whereby the accumulation of *CO* mRNA in the night of LDs is, in part, controlled by COP1 (Yu *et al.*, 2008). In LDs, the night-time peak of *CO* mRNA rhythms is lost in the *cop1* mutant (Supplementary Fig 19C). Furthermore, now that COP1 activity in the circadian clock has been added to the clock model, we are able to use COP1 as a night-time activator of *CO* mRNA. Comparisons of the resulting simulations of the *cop1* mutant show qualitative similarities to published transcript profiles (Supplementary Fig 19). Notably, *FT* mRNA accumulates to a higher level during the night of SDs in *cop1* mutants compared to the WT (Supplementary Fig 19F) and the LD-specific night-time peak of *CO* mRNA is lost in the *cop1* mutant (Supplementary Fig 19G).

Since *CDF1* mRNA and *FKF1* mRNA were fitted to datasets from WT and *cca1;lhy* backgrounds, we are able to obtain good quantitative matches to these datasets (Fig 2A & B). The mutation of CCA1/LHY leads to an advanced phase of *CDF1* and *FKF1* mRNA expression, decreasing the amplitude of *CDF1* and marginally increasing the amplitude of *FKF1* rhythms (Fig 2A & B and Supplementary Figs 1A & 2A). This supports our hypothesis that *CDF1* mRNA is activated by CCA1/LHY whereas *FKF1* transcription is inhibited (Eqs 1 & 4). These connections have been validated experimentally using chromatin immunoprecipitation (ChIP) (Fig 2B and Supplementary Fig 3). However, our model is currently unable to fully describe the rhythms of *CO* and *FT* mRNA in *cca1;lhy* mutants (Supplementary Fig 1E & F and 2E & F). The model is able to correctly produce an earlier phase of expression of *CO* and *FT* mRNA in both SD and LD, but the model fails to maintain the increased expression of *CO* and *FT* rhythms observed in the double mutants throughout LD cycles. As such, we can propose that CCA1/LHY must play a role in the system either downstream of or in parallel to *CDF1* mRNA to regulate *CO* (and maybe *FT*) mRNA. This is similar to the case shown in Salazar *et al.*, 2009 where *FT* mRNA could not be described in an *fkf1* mutant despite an accurate description of *CO* mRNA. FKF1 was hypothesised to play a role with CO protein and this was validated experimentally (Song *et al.*, 2012). Interestingly, the *cca1;lhy* mutant’s effect on the amplitude of *CO* mRNA expression is relatively minor, whereas the effects on *FT* expression amplitude are considerably larger, particularly in SD (Supplementary Fig 1B & C). Based on this observation, we can postulate two potential mechanisms by which CCA1/LHY regulate *CO* and *FT* mRNA: first, downstream of *CDF1* mRNA, by helping CDF1 protein inhibit the transcription of *CO* and *FT* mRNA; and, second, in parallel with CDF1 protein by playing a role in the COP1-dependent activation of the night-time peak in LD *CO* expression. Interestingly, a circadian regulated NAC protein, LONG VEGETATIVE PHASE 1 (LOV1) has recently been found to suppress the night-time expression of *CO* mRNA in a pathway that may be independent to the classic photoperiodic flowering pathway (Yoo *et al*., 2007). Thus, CCA1/LHY may regulate *CO* mRNA through the LOV1 pathway independently of CDF1.

## The model of PIF4 and PIF5 describes circadian clock mutants and multiple WT datasets

The photoperiod-dependent pathway that regulates rhythmic hypocotyl elongation has been quantitatively characterised over a wide range of conditions, such that the data available are now comparable to those used in modelling the flowering time pathway. The summary of data used (Supplementary Table 4) illustrates that expression data for central components of both pathways (e.g. *CO* and *PIF4* mRNA) is available across a range of photoperiods and in many clock mutants. However, there is still a shortfall in quantified protein level data for components in the hypocotyl module. Quantified protein expression rhythms have been obtained for flowering components, such as FKF1 and CDF1, in different photoperiods and genetic backgrounds, but not for components of the hypocotyl elongation pathway (although blots of PIF4 protein levels have recently been compared in two photoperiods; Yamashino *et al.*, 2013). The acquisition of such data will be important in the development of more detailed models of hypocotyl elongation.

A key feature of the model is the regulation of PIF activity by two distinct mechanisms – PIF proteolysis by phyB and PIF sequestration by a set of light-stimulated ‘Interactors’. While the stimulation of PIF proteolysis by phyB is considered to be the major regulatory pathway responsible for suppression of PIF activity, it is clear from observed PIF target dynamics in *phyB* mutant lines that a second light-responsive pathway acts to significantly repress PIF activity in the light (Nomoto *et al.*, 2012a). This is further suggested by the observation that the hypocotyl response to blue light photoperiods is lost in the *pif4;5* mutant (Kunihiro *et al.*, 2010). PIF protein activity is also regulated by phyB (Park *et al.*, 2012) and a variety of other proteins through direct interaction and sequestration (e.g. BZR1 (Bai *et al.*, 2012; Oh *et al.*, 2012), DELLAs (de Lucas *et al.*, 2008; Feng  *et al.*, 2008), HFR1 (Foreman *et al.*, 2011; Hornitschek *et al.*, 2009) and PAR1 (Hao *et al.*, 2012)). By including these forms of regulation, the model is able to describe the differing dynamics and behaviour of PIFs under SD and LD conditions.

In SD conditions, *PIF* mRNA expression levels are higher both at dawn and at dusk when compared to LDs (Fig 4B). The higher expression levels at dawn, together with the fall off in PIF protein turnover in the night due to gradual inactivation (dark reversion) of phyB, mean that PIF protein levels are high at dawn in SDs (Supplementary Fig 11C). Immediately following dawn, light-activation of phyB-mediated PIF turnover results in a rapid decline in PIF protein levels. These dynamics at the PIF protein level result in the dawn peak of *ATHB2* and *IAA29* expression in SDs (Fig 4C,D).

The peak in PIF activity at the end of night in short days was a key model behaviour which the model was constrained to fit. However, interestingly, the model simulations were also able to suggest other conditions and times of day at which PIF protein levels may be sufficient to allow PIFs to perform additional regulatory functions. For example, the high *PIF* mRNA expression at dusk observed specifically at SDs is predicted to give rise to substantial PIF protein levels at this time (Supplementary Fig 11C). As discussed in the Main Text this behaviour in PIF protein levels results in a PIF-dependent peak in expression of *IAA29* in the early night, specifically in SDs (Fig 4D).

## Photoperiodic physiology

As shown in Fig 5C & D, the model is able to match accurately the published hypocotyl and flowering responses observed in WT plants across a range of photoperiods. However, the functions used above (Eqs 24 & 25) are not constrained for similar analysis of mutant plants. Thus, to approximate the phenotypes of mutants we compared the values of *ATHB2_AREA_* and *FT_AREA_* between WT and mutant simulations. From these we can approximate whether a mutant plant has a relatively longer or shorter hypocotyl with delayed or earlier flowering than the WT plant (Supplementary Table 3). As seen in Supplementary Table 3, a number of the key mutants simulated match the observed phenotypes.

## Sensitivity analysis

To determine whether the model system was particularly sensitive to perturbations in individual parameters a local sensitivity analysis was carried out (Supplementary Fig 20). Each parameter value was increased by 1% in turn and the % change in *FT_AREA_* and *ATHB2_AREA_* recorded (or, equivalently, the % change in average *FT* and *ATHB2* expression). This allows us to determine the relative sensitivity of these quantities to changes in parameters. For a quantity *Q*, its relative sensitivity to changes in parameter *k* is given by:

$S_{k}^{Q}=\frac{d(\ln\left( Q \right))}{d(\ln\left( k \right))}=\frac{k}{Q}\cdot\frac{dQ}{dk}$ (28)

This can be approximated from the simulation data described above by:

$S_{k}^{Q}\approx\frac{k_{0}}{Q_{0}}\cdot\frac{{(Q-Q}_{0})}{(k-k_{0})}$ (29)

Where ${(k-k_{0})}/{k_{0}}=0.01$ in this case. Relative sensitivities with magnitude greater than 1 indicate greater-than-proportional relative changes in output to changes in the parameter. This analysis highlighted a small subset of parameters that, when changed, impacted the model dynamics, thus making them sensitive parameters. A number of these were related to areas of the model that are not well constrained. Notably, altering the COP1- and dark-dependent degradation of CO protein (parameter *m_6_*) had a large effect on *FT_AREA_* (Supplementary Fig 20). This can be explained by a combination of two effects. First, it is clear that reducing the degradation rate of CO protein will lead to an increase in CO protein levels, and therefore an increase in *FT* levels. Second, this reduction in degradation rate also extends the time window during which CO protein is present, coinciding with the later ZTs during which more *CO* transcript is present.

## Random perturbation of parameters

One of the interesting observations from model simulations was the SD-specific increase in PIF-induced transcript expression in the early night. This is seen in *IAA29* (Fig 4C), and in several other PIF-induced transcripts (Nomoto *et al*., 2012b). A natural interpretation of this behaviour is that the presence of *PIF4* and *PIF5* transcript in the early night in SDs can lead to a secondary peak in PIF activity at this time. In order to assess how robust this feature of the model is to changes in parameters, we performed a random parameter perturbation experiment. Specifically, we perturbed the parameters *g_14_*, *g_9_*, *n_11_*, *p_12_*, *p_13_*, *m_13_*, *m_14_*, *p_10_*, *p_11_*, and *m_12_*. Changing these parameters changes the dynamics of PIF proteins (Eq 12), Interactor proteins (Eq 11), and a PIF-induced transcript (e.g. as in Eq 13) while leaving the dynamics of photoreceptors (Eqs 9 & 10) and *PIF* transcripts (Eqs 7 & 8) unchanged. Each parameter was perturbed according to:

$p_{new}=2^{X}p_{old}$ (30)

The random variable *X* was drawn from a standard normal distribution ($X \sim N(0,1)$), with the result that approximately 30% of parameters are expected to be perturbed by more than 2-fold overall. After perturbation of parameters, the resulting model was simulated in SD and LD conditions (Supplementary Fig 12). The diversity of PIF and Interactor dynamics observed in simulations demonstrates that these parameter perturbations were sufficient to strongly affect the model dynamics. Parameter sets in which the PIF-induced transcript dynamics departed qualitatively from known dynamics of PIF activity were then filtered out. This includes, for example, cases where the PIF-induced transcript was not expressed in either condition (such cases are expected given the magnitude of the parameter perturbations).

Parameter sets were classified in two stages according to the simulated dynamics of PIF-induced transcript. First, classification of dynamics as significantly bimodal in SDs was determined according to whether a secondary peak and subsequent trough was observed during the time window ZT12-18, and whether its peak-to-trough ratio was greater than 1.2. Of the remaining parameter sets, dynamics were classified as having a SD-specific increase in the early night if there was a greater than 3-fold induction (measured at maximal expression) in SDs than LDs during the time window ZT8-20. The remaining parameter sets displayed neither behaviour, and by process of elimination only displayed a SD-specific peak at ZT0. The ability of this procedure to distinguish between qualitatively distinct dynamics is apparent from the classification of 20 example parameter sets presented in Supplementary Fig 12.

# Supplementary Tables

**Supplementary Table 1:** PIF-induced transcripts displaying dynamics consistent with induction by PIFs in light:dark cycles.

**Supplementary Table 2:** PIF-repressed transcripts displaying dynamics consistent with induction by PIFs in light:dark cycles.

**Supplementary Table 3**: Predicted hypocotyl and flowering phenotypes of WT and mutant plants in SDs (8L:16D) and LDs (16L:8D). Phenotypes given in brackets are the phenotypes observed in the reference publications. E = Early flowering; D = Delayed flowering; S = Short hypocotyl; L = Long hypocotyl.

|  | Short Days | | Long Days | |  |
| --- | --- | --- | --- | --- | --- |
| Line | Hypocotyl Length | Flowering Time | Hypocotyl Length | Flowering Time | Reference Publication(s) |
| *cca1;lhy* | L (S) | E (E) | L (~WT) | D (~WT) | Mizoguchi *et al.*, 2002  Niwa *et al.*, 2009 |
| *CCA1ox* | L (L) | D (D) | L (L) | D (D) | Niwa *et al*., 2009  Lu *et al*., 2012 |
| *prr9;7* | L (L) | D (D) | L (L) | D (D) | Nakamichi *et al.,* 2007  Niwa *et al.*, 2009 |
| *elf3* | L (L) | E (E) | L (L) | E (E) | Yu *et al.*, 2008  Lu *et al.*, 2012 |
| *ztl* | L (S) | E (E) | L (ND) | E (~WT) | Somers *et al.*, 2004  Kim *et al.*, 2005  Takase *et al.*, 2011 |
| *gi* | L (L) | D (D) | L (L) | D (D) | Yu *et al.*, 2008  Fornara *et al.*, 2009 |
| *fkf1* | - | D (D) | - | D (D) | Imaizumi *et al.*, 2005 |
| *CO-ox* | - | E (E) | - | E (E) | Song *et al.*, 2012 |
| *CO-ox;*  *CDF1-ox* | - | E (E) | - | D (~WT) | Song *et al.*, 2012 |
| *cdf* | - | E (E) | - | E (E) | Fornara *et al.*, 2009 |
| *pif4;5* | S (S) | D (D) | S (S) | D (ND) | Niwa *et al*., 2009  Kumar *et al.*, 2012 |

*ND = not determined or was unable to be found in the literature.

**Supplementary Table 4**: List of datasets used in this study. Those with a (*) were used as training datasets to optimise the model.

| Genotype | Model components | Conditions | Study |
| --- | --- | --- | --- |
| WT | *CO, FT* | 8L:16D | Imaizumi *et al*., 2003 |
|  | *CO, FT* |  | Yu *et al*., 2008 |
|  | *CO, FT* |  | Sawa and Kay, 2011 |
|  | *CO, FT* |  | Lu *et al*., 2012 |
|  | *FT* | (22°C & 27°C) | This study (Supplementary Dataset 6) |
|  | *PIF4(*), PIF5(*)* |  | Nusinow *et al*., 2011 |
|  | *PIF4, ATHB2*(*)*, IAA29*(*) |  | Nomoto *et al.*, 2012b |
|  | CDF1 |  | Imaizumi *et al*., 2005 |
|  | *CO*, *FT* | 10L:14D | Nakamichi *et al.*, 2007 |
|  | *CDF1, FKF1*(*) |  | Niwa *et al.*, 2007 |
|  | *PIF4*(*), *PIF5*(*) | 12L:12D | Nusinow *et al.*, 2011 |
|  | *CDF1*(*), *FKF1, CO*, *FT* | 16L:8D | Nakamichi *et al.*, 2007 |
|  | *CDF1, CO, FT* |  | Nakamichi *et al.*, 2007 |
|  | *CO, FT* |  | Imaizumi *et al*., 2003 |
|  | *CO, FT* |  | Yu *et al*., 2008 |
|  | *CO, FT* |  | Sawa and Kay, 2011 |
|  | *CO, FT* |  | Lu *et al*., 2012 |
|  | *CO* |  | Sawa *et al*., 2007 |
|  | *CO, FT* | (22°C & 27°C) | This study  (Supplementary Datasets 3 & 5) |
|  | *PIF4(*), PIF5(*)* |  | Nusinow *et al*., 2011 |
|  | *PIF4, ATHB2*(*)*, IAA29*(*) |  | Nomoto *et al.*, 2012b |
|  | *PIF4(*),ATHB2(*),IAA29(*)* | (22°C & 28°C) | Nomoto *et al*., 2012a |
|  | CDF1(*), FKF1(*) |  | Song *et al*., 2012 |
|  | CDF1 |  | This study (Supplementary Dataset 2) |
|  | CO | (22°C & 27°C) | This study (Supplementary Dataset 4) |
| *prr9;7* | *CDF1, FKF1, CO, FT* | 16L:8D | Nakamichi *et al.*, 2007 |
| *prr9;7;5* | *PIF4, ATHB2, IAA29* | 8L:16D | Nomoto *et al.*, 2012b |
| *cca1;lhy* | *CO, FT* | 10L:14D | Nakamichi *et al.*, 2007 |
|  | *CDF1, FKF1*(*) |  | Niwa *et al.*, 2007 |
|  | *CDF1*(*)*, CO, FT* | 16L:8D | Nakamichi *et al.*, 2007 |
| *CCA1ox* | *CO, FT* | 8L:16D | Lu *et al*., 2012 |
|  | *CO, FT* | 16L:8D | Lu *et al*., 2012 |
| *gi* | *CO, FT* | 8L:16D | Sawa and Kay, 2011 |
|  | *CO, FT* | 16L:8D | Sawa and Kay, 2011 |
|  | *CO* |  | Sawa *et al.*, 2007 |
|  | CDF1 |  | This study (Supplementary Dataset 2) |
| *elf3* | *CO, FT* | 8L:16D | Lu *et al*., 2012 |
|  | *CO, FT* | 16L:8D | Lu *et al.*, 2012 |
|  | *PIF4, ATHB2, IAA29* |  | Nomoto *et al*., 2012b |
| *CCA1ox;elf3* | *CO, FT* | 8L:16D | Lu *et al*., 2012 |
|  | *CO, FT* | 16L:8D | Lu *et al*., 2012 |
| *cop1* | *CO, FT* | 8L:16D | Yu *et al.*, 2008 |
|  | *CO, FT* | 16L:8D | Yu *et al.*, 2008 |
| *fkf1* | *CO, FT* | 8L:16D | Imaizumi *et al*., 2003 |
|  | *CO*(*), *FT*(*) | 16L:8D | Imaizumi *et al.*, 2003 |
|  | *CO* |  | Sawa *et al*., 2007 |
|  | CDF1(*) |  | Song *et al.*, 2012 |
| *CO-ox;fkf1* | *FT* | 16L:8D | Song *et al.*, 2012 |
| *CO-ox;CDF1-ox* | *FT* | 16L:8D | Song *et al.*, 2012 |
| *pif4;5* | *CO, FT*(*) | 16L:8D (22°C & 27°C) | This study (Supplementary Datasets 3 & 5) |
| *pif4* | *FT* | 8L:16D (22°C & 27°C) | This study (Supplementary Dataset 6) |

**Supplementary Table 5**: Primers used in chromatin immunoprecipitation assay (see Supplementary Fig 3).

| Gene | Left Primer (5’-…-3’) | Right Primer (3’-…-5’) |
| --- | --- | --- |
| *Actin (ACT7)* | GTATCGGGTGACAATGCAGCTATTA | TGCTGGAGTAAAACATAAGCCACTC |
| *GI-a* | gtggcaaaggcaaggaaata | tctctctcctaaggccacca |
| *GI-N* | gacaggcctgagtcactggt | TGGAATGCTTGTTGATGGAG |
| *FKF1-a* | cgagaatcgcgtttcacaaa | aatatcccctggtgacgtgt |
| *FKF1-b* | acgaaaattgccaccaactc | aaaATGGCGAGAGAACATGC |
| *FKF1-N* | GATTGCAGGGCTTCACTCTC | CGTCATGGAGGATCCTGAAT |
| *CDF1-a* | cgcgatgctgacatttacct | attgcatcctcgtaggagca |
| *CDF1-b* | ttggttaacggaaagtttagtga | cacgatttccaaaccaaaa |
| *CDF1-N* | TGGACAACACTTGGGATCAA | TGTTCTTTGTGCAAACCCTG |
| *PIF4-a* | ccaatctgccgacaagtttc | acaccgtaacaccatcacga |
| *PIF4-b* | ccacgtgtcgttcatttcaa | gatagagagttgtgttgggcg |
| *PIF4-N* | CGGAGTTCAACCTCAGCAGT | CAATTCAGAACAATCCCGGT |
| *PIF5-a* | taggcccaataacgcatctc | tatcggtttagaagatgatggaa |
| *PIF5-b* | gtccctccttgctcgatttt | tggagagggttgtttggttt |
| *PIF5-c* | tggagagggttgtttggttt | tgacATGGAACAAGTGTTTGC |
| *PIF5-N* | GGACATGTTGGGATTTGGAT | GACCACCGACAGTCTTCATATG |

**Supplementary Table 6:** Parameter values for model (separate file).

**Supplementary Table 7:** BioDare timeseries data IDs (separate file).

# Supplementary Files

**Supplementary File 1**: MATLAB code allowing simulation of the complete model.

**Supplementary File 2**: Collected literature timeseries data.

# Supplementary Datasets

**Supplementary Dataset 1**: CCA1 ChIP data

**Supplementary Dataset 2**: CDF1 protein; CDF1ox, CDF1;gi; 16L:8D; 22°C

**Supplementary Dataset 3**: CO mRNA; WT, *pif4;pif5*; 16L:8D; 22°C, 27°C

**Supplementary Dataset 4**: CO protein; WT; 16L:8D; 22°C, 27°C

**Supplementary Dataset 5**: FT mRNA; WT, *pif4;pif5*; 16L:8D; 22°C, 27°C

**Supplementary Dataset 6**: FT mRNA; WT, *pif4*; 8L:8D; 22°C, 27°C

#

# Supplementary Figure Legends

**Supplementary Figure 1: Simulations of 'Flowering Module' in *cca1;lhy* mutant in SDs.** (A-C) *CDF1, CO* and *FT* mRNA data in WT (black lines, filled squares) and the *cca1;lhy* mutant (green lines, open circles) in SDs. (A) *CDF1* mRNA data from Niwa *et al.*, 2007. (B, C) *CO* and *FT* mRNA from Nakamichi *et al.*, 2007. Error bars represent standard deviation. (D-F) Simulated *CDF1, CO* and *FT* mRNA levels in WT (black lines) and the *cca1;lhy* mutant (dashed green lines) in SDs.

**Supplementary Figure 2: Simulations of 'Flowering Module' in *cca1;lhy* mutant in LDs.** (A-C) *CDF1, CO* and *FT* mRNA data in WT (black lines, filled squares) and the *cca1;lhy* mutant (green lines, open circles) in LDs. Data from Nakamichi *et al.*, 2007. Error bars represent standard deviation. (D-F) Simulated *CDF1, CO* and *FT* mRNA levels in WT (black lines) and the *cca1;lhy* mutant (dashed green lines) in LDs.

**Supplementary Figure 3: CCA1 binding to CBS and EE motifs on target promoters.** (A) Schematic showing locations of primers across genomic regions of target genes *GI*, *FKF1*, *CDF1*, *PIF4* and *PIF5*. The labels ‘-a’, ‘-b’, and ‘-c’ denote locations with putative CCA1 binding sites (i.e. EE/CBS motifs). The label ‘-N’ denotes an exonic region. (B) ChIP assay showing CCA1 activity across two biological replicates (black and white bars). Plants were grown for 14 days 12L:12D cycles at 22°C and harvested at ZT2. 1-tailed Student’s t-test used to calculate significance of CCA1 binding at CBS and EE promoter motifs compared to the gene’s exon; (*) p < 0.05, (**) p < 0.005. Error bars represent standard error.

**Supplementary Figure 4: Quantitative match of protein models against training data.** Simulations of (A) CDF1 and (B) FKF1 protein compared to data used to optimise model. WT data = solid black lines, filled squares; WT simulation = solid black line; *fkf1* data = dashed blue lines, empty squares; *fkf1* simulation = dashed blue line. Data taken from Song *et al.*, 2012 (see Supplementary Table 3). Error bars represent standard error.

**Supplementary Figure 5. Comparison of measured and simulated CO and FT dynamics in the *fkf1* mutant in SDs and LDs.** (A-D) *CO* and *FT* mRNA data in WT (black lines, filled squares) and the *fkf1* mutant (blue lines, open circles) in SDs (A,B) and LDs (C,D). Data from (Imaizumi *et al*., 2003), normalised to the peak level in across both conditions (as in Imaizumi *et al*., 2003). Error bars represent the standard error. (E-H) Simulated *CO* and *FT* mRNA levels in WT (black lines) and the *fkf1* mutant (dashed blue lines) in SDs (E,F) and LDs (G,H).

**Supplementary Figure 6. Comparison of measured and simulated CO and FT dynamics in the *gi* mutant in SDs and LDs.** (A-D) *CO* and *FT* mRNA data in WT (black lines, filled squares) and the *gi* -2mutant (red lines, open circles) in SDs (A,B) and LDs (C,D). Data from (Sawa and Kay, 2011), normalised relative to internal reference (as in Sawa and Kay, 2011). (E-H) Simulated *CO* and *FT* mRNA levels in WT (black lines) and the *gi-2* mutant (dashed red lines) in SDs (E,F) and LDs (G,H).

**Supplementary Figure 7. Comparison of measured and simulated CO and FT dynamics in the *elf3, CCA1ox,* and *CCA1ox;elf3* mutants in SDs and LDs.** (A-D) *CO* and *FT* mRNA data in WT (black lines, filled squares), and the *elf3* (blue lines, open circles), *CCA1ox* (green lines, open circles), and *CCA1ox;elf3* (red lines, open circles) mutants in SDs (A,B) and LDs (C,D). Data from (Lu *et al*., 2012), normalised to the peak expression in WT in LDs. Error bars represent standard deviation. (E-H) Simulated *CO* and *FT* mRNA levels in WT (black lines) and the *elf3* (dashed blue lines), *CCA1ox* (dashed green lines), and *CCA1ox;elf3* (dashed red lines) mutants in SDs (E,F) and LDs (G,H).

**Supplementary Figure 8: Comparison of results from previous flowering time model with new model.** Key results of previous flowering model are recapitulated with the updated model presented here (for comparison see Song *et al.*, 2012). (A) *FT* mRNA in WT, *fkf1*, *CO-ox;fkf1* and *CO-ox;CDF1-ox* (errorbars for this line were unavailable) lines are simulated and compared to data. WT data: black lines, filled squares; simulations = black lines. *fkf1* data: blue lines, open circles; simulation: blue dashed lines; *CO-ox;fkf1* data: green lines, open squares; simulations: green dashed-dotted lines. *CO-ox;CDF1-ox* data: pink lines, open squares; simulations: pink dashed-dotted lines; (B) *FT* mRNA in WT, *fkf1*, Δ(1) (i.e. no FKF1-CDF1 interaction) and Δ(2) (i.e. no FKF1-CO interaction) *fkf1*-partial mutants (see Song *et al.*, 2012). Δ(1): purple dashed-dotted line; Δ(2): purple dashed line. WT and *fkf1* as in (A). Data from WT and *fkf1* lines taken from Imaizumi *et al.*, 2003. Data from *CO-ox;fkf1* and *CO-ox;CDF1-ox* taken from Song *et al.*, 2012. Error bars represent standard error.

**Supplementary Figure 9. Model describes PIF target dynamics in mutants of EC components.** (A-C) *PIF4*, *IAA29*, and *ATHB2* mRNA data in WT (black lines, filled squares) and the *elf3* mutant (blue lines, open circles). Data from (Nomoto *et al.,* 2012b), normalised to the peak level in *elf3*. Error bars represent standard deviation. (D-F) *PIF4*, *IAA29*, and *ATHB2* mRNA simulation results in WT (black lines) and the *elf3* mutant (dashed blue lines). (G-J) Comparison of model simulation with microarray data from the two largest clusters of PIF targets clustered in WT (G,I) and the *lux* mutant (H,J). Data from (Michael *et al*., 2008a), normalised by the mean expression level across both conditions.

**Supplementary Figure 10. Model describes PIF target dynamics in mutants of with defective circadian clocks.** (A-C) *PIF4*, *IAA29*, and *ATHB2* mRNA data in WT (black lines, filled squares) and the *prr9;7;5* mutant (red lines, open circles). Data from (Nomoto *et al.,* 2012b), normalised to the peak level in *prr9;7;5*. Error bars represent standard deviation. (D-F) *PIF4*, *IAA29*, and *ATHB2* mRNA simulation results in WT (black lines) and the *prr9;7;5* mutant (dashed red lines). (G-J) Comparison of model simulation with microarray data from the two largest clusters of PIF targets clustered in WT (G,I) and the *LHYox* mutant (H,J). Data from (Michael *et al*., 2008a), normalised by the mean expression level across both conditions.

**Supplementary Figure 11: Regulation of PIF protein levels in WT and *PIF4ox*.** (A) Simulated abundance of active phyB in SDs (blue solid line) and LDs (red solid line). (B) As in (A) for ’Interactor’ protein abundance. (C) As in (A) for total PIF4,5 protein abundance. (D) Simulated PIF4 protein abundance in *PIF4ox* in SDs (blue dashed-dotted line) and LDs (red dashed-dotted line).

**Supplementary Figure 12. Robustness of PIF model behaviour to random parameter perturbations.** Example results from 20 parameter sets are plotted in LDs (A-C) and SDs (D-F). (A,D) Simulated PIF Interactor protein levels. (B,E) Simulated PIF protein levels. (C,F) Simulated PIF-induced transcript dynamics (normalised to the peak in SDs). Parameters were randomly perturbed and classified according to whether the PIF-induced transcript displayed SD-specific bimodal dynamics (blue lines), a SD-specific increase in the early night (green lines), or only a SD-specific peak at ZT0 (black lines). Of 96 parameter sets tested, 38 displayed bimodal dynamics and a further 28 displayed a post-dusk increase, with the remaining 30 displaying only the peak at ZT0 (see Supplementary Information for details of classification procedure).

**Supplementary Figure 13. Summary of overlap between PIF-regulated and PIF-bound genes.** (A) Overlap between PIF-bound genes and those that have dynamics consistent PIF-induced behaviour in light:dark cycles. (B) As in (A), for PIF-repressed genes. (*) denotes statistically significant overlap between sets (p<10^-8^, hypergeometric test). PIF-bound genes are genes identified as bound by PIF4 (Oh *et al*., 2012) or PIF5 (Hornitschek *et al*., 2012) by ChIP-Seq, as collected in (Zhang *et al*., 2013).

**Supplementary Figure 14. Summary of clustering results for PIF-repressed genes.** Dynamics of the four clusters with behaviour consistent with PIF repression in light:dark cycles are shown in (A) SDs and LDs (data from Michael *et al*., 2008b), (B) WT and the *lux* mutant (data from Michael *et al*., 2008a), and (C) WT and the *LHYox* mutant (data from Michael *et al*., 2008a). See Supplementary Information for details of normalisation and clustering analysis.

**Supplementary Figure 15. Comparison of model simulation with microarray data for the largest cluster of PIF-repressed genes.** Results for Cluster 1, containing 68 genes, are shown in (A,B) SDs and LDs (cluster exemplar data: red lines; simulation: black lines) (data from Michael *et al*., 2008b), (C,D) WT and the *lux* mutant (data from Michael *et al*., 2008a), and (E,F) WT and the *LHYox* mutant (data from Michael *et al*., 2008a). See Supplementary Information for details of normalisation and clustering analysis.

**Supplementary Figure 16. Cluster analysis of PIF targets in constant light conditions, under 12H:12C temperature cycles.** (A) *PIF4* and *PIF5* microarray timecourses. (B) Microarray timecourse data for PIF-induced target clusters. (C) Distribution of peak phases of PIF-induced target genes. (D) Microarray timecourse data for the PIF-repressed target clusters. (E) Distribution of peak phases of PIF-repressed target genes. Data from (Michael *et al*., 2012b). Peak phases were defined as in the DIURNAL database (Mockler *et al*., 2007), with a self-correlation cut-off of 0.8 applied to filter out arrhythmic transcripts.

**Supplementary Figure 17.** Cluster analysis of PIF targets in the *phyB* mutant. (A) Microarray timecourse data for the PIF-induced clusters WT and the *phyB* mutant. (B) as in A, for PIF-repressed clusters. Data from Michael *et al*., 2008a.

**Supplementary Figure 18. Temperature response of *FT* in WT and *pif4* in 8L:16D conditions.** Plants were grown for 7 days in 8L:16D cycles on agar plates at 22°C, then transferred to soil and grown for a further 21 days at either 22°C or 27°C. Error bars represent the standard error.

**Supplementary Figure 19: COP1 as a proxy for the NIGHT-TIME PEAK component of the 'Flowering Module'.** (A) *CO* mRNA data in WT (solid line, filled squares) and *cop1* mutants (dashed line, empty squares) in SDs. (B) As in (A), data of *FT* mRNA. (C and D) As in (A and B) for LDs. (E-H) Simulated *CO* and *FT* mRNA in WT (solid line) and the *cop1* mutant (dashed line). Data taken from Yu *et al.*, 2008. Error bars represent standard deviation.

**Supplementary Figure 20. Parameter sensitivity analysis.** The sensitivity of *FT_AREA_* and *ATHB2_AREA_* to changes in parameters is shown. A relative sensitivity of 1 indicates that a relative change in the value of a parameter is matched by the same relative change in the output, see Supplementary Information for details. (*) the relative sensitivity of *FT_AREA_* for the parameter *m_6_* is = -6.0 (*m_6_* specifies the rate of CO protein degradation by COP1 in the dark).

**Supplementary Figure 21. Change in average level of PIF-induced targets with photoperiod and under genetic perturbations.** Significant differences were identified in the responses between Cluster 1 and Cluster 2 in each case (difference in medians evaluated by Mann-Whitney U-test; * p < 0.05, ** p < 10^-3^, *** p < 10^-9^). Data from (Michael *et al*., 2008a; Michael *et al*., 2008b).
