# Supplementary material for: Linked circadian outputs control elongation growth and flowering in response to photoperiod and temperature
Source: Mol Syst Biol. 2015 Jan 19;11(1):776. doi: 10.15252/msb.20145766 (PMC4332151; doi:10.15252/msb.20145766)
Supplement: Supplementary file 35 [file msb0011-0776-sd35.docx]

**Supplementary References**

Buchler NE, Cross FR (2009) Protein sequestration generates a flexible

ultrasensitive response in a genetic network. Mol Syst Biol 5: 272

Clack T, Shokry A, Moffet M, Liu P, Faul M, Sharrock RA (2009) Obligate

heterodimerization of Arabidopsis phytochromes C and E and interaction

with the PIF3 basic helix-loop-helix transcription factor. Plant Cell 21:

786 – 799

Feng S, Martinez C, Gusmaroli G, Wang Y, Zhou J, Wang F, Chen L, Yu L,

Iglesias-Pedraz JM, Kircher S, Schäfer E, Fu X, Fan LM, Deng XW (2008)

Coordinated regulation of Arabidopsis thaliana development by light and

gibberellins. Nature 451: 475 – 479

Jang S, Marchal V, Panigrahi KCS, Wenkel S, Soppe W, Deng XW, Valverde F,

Coupland G (2008) Arabidopsis COP1 shapes the temporal pattern of CO

accumulation conferring a photoperiodic flowering response. EMBO J 27:

1277 – 1288

Kunihiro A, Yamashino T, Mizuno T (2010) PHYTOCHROME-INTERACTING

FACTORs PIF4 and PIF5 are implicated in the regulation of hypocotyl

elongation in response to blue light in Arabidopsis thaliana. Biosci

Biotechnol Biochem 74: 2538 – 2541

Rausenberger J, Hussong A, Kircher S, Kirchenbauer D, Timmer J, Nagy F,

Schäfer E, Fleck C (2010) An integrative model for phytochrome B

mediated photomorphogenesis: from protein dynamics to physiology. PLoS

ONE 5: e10721

Yoo SY, Kim Y, Kim SY, Lee JS, Ahn JI (2007) Control of flowering time and

cold response by a NAC-domain protein in Arabidopsis. PLoS ONE 7: e642
