# Supplementary material for: Linked circadian outputs control elongation growth and flowering in response to photoperiod and temperature
Source: Mol Syst Biol. 2015 Jan 19;11(1):776. doi: 10.15252/msb.20145766 (PMC4332151; doi:10.15252/msb.20145766)
Supplement: Supplementary file 36 [file msb0011-0776-sd36.pdf]

molecular systems biology

Linked circadian outputs control elongation growth and development in response to photoperiod and temperature

Daniel Seaton, Robert Smith, Steven Penfield, Dana MacGregor, Takato Imaizum, Andrew Millar, Young Hun Song, Kelly Stewart, Gavin Steel, Julia Foreman and Karen J. Halliday

Corresponding author: Karen J. Halliday, University of Edinburgh

Review timeline:

Submission date:	11 October 2013
Editorial Decision:	21 November 2013
New submission:	15 September 2014
Editorial Decision:	29 October 2014
Revision received:	21 November 2014
Accepted:	5 Decemebr 2014

Editor: Thomas Lemberger/Maria Polychronidou

Transaction Report:

(Note: With the exception of the correction of typographical or spelling errors that could be a source of ambiguity, letters and reports are not edited. The original formatting of letters and referee reports may not be reflected in this compilation.)

1st Editorial Decision 21 November 2013

Thank you again for submitting your work to Molecular Systems Biology. We have now heard back from the two referees whom we asked to evaluate your manuscript. As you will see from the reports below, the referees acknowledge that you address a potentially interesting topic. However, they raise substantial concerns on your work, which, I am afraid to say, preclude its publication in Molecular Systems Biology.

While reviewer #2 is overall positive, reviewer #1 raises significant concerns regarding the conclusiveness of the main findings. In particular, reviewer #1 points out that the study remains preliminary and that additional experimental evidence is required for convincingly supporting the proposed link between the response to high temperature and the regulation of CONSTANS. Moreover, this referee refers to the need to provide additional controls and to cautiously consider potential discrepancies when incorporating previously published data.

Considering these rather substantial concerns, we feel that we have no choice but to return this manuscript with the message that we cannot offer to publish it.

Nevertheless, as the reviewers did have positive words for the goals of the study and both felt that the proposed link between the photoperiodic and high temperature responses, we would like to indicate that we would be willing to reconsider a new submission based on this work. Any new submission would need to include new experimental data rigorously addressing the concerns raised by the reviewers.

A resubmitted work would have a new number and receipt date. We recognize that this would involve substantial additional experimentation and analysis and, as you probably understand, we can

© European Molecular Biology Organization

give no guarantee about its eventual acceptability. If you do decide to follow this course then it would be helpful to enclose with your re-submission an account of how the work has been altered in response to the points raised in the present review.

I am sorry that the review of your work did not result in a more favorable outcome on this occasion, but I hope that you will not be discouraged from sending your work to Molecular Systems Biology in the future. In any case, thank you for the opportunity to examine this work.

Reviewer #1:

This paper describes an analysis of environmental control of flowering in Arabidopsis thaliana, linking photoperiodic and high temperature responses using a combination of mathematical modelling and experimental approaches. The manuscript starts by refining the authors¥ previous model of photoperiodic flowering, establishing that the clock component CCA1 binds directly to the CDF1 and FKF1 promoters. They then propose that ELF3 protein regulates CDF1 protein stability or activity, based on the observations that GI cannot fully explain the pattern of CDF1 protein observed and that GI and ELF3 were previously shown to regulate each other's activity. Analysis of CO and FT mRNAs together with further modelling provides some support for this proposal. The circadian regulation of PIF transcription factors was examined and modelled, and the authors found CCA1 binds directly to PIF5 promoter. Previously PIF4 was shown to promote flowering in short days at high temperature through activation of FT. The authors show that pif4 pif5 double mutants exhibit higher FT expression in long days at high temperature and therefore that these PIFs are not required for high temperature promotion of flowering in long days. However they find that CO protein is stabilized at high temperature in long days and propose that this causes FT expression to increase.
This paper makes some interesting suggestions about connections within the photoperiodic flowering pathway and its response to high temperature. The proposed effect of high temperature on CO protein is interesting and extends the published data but is preliminary. Overall the paper covers a wide range of topics and the experimental support for several of the proposals is weak. Specific points are:

1. Figure 5D. The increase in CO protein at high temperature in long days directly links photoperiodic signaling and high temperature response. However in this figure CO protein is increased at high temperature between dawn and 8h. This difference is found in a CO overexpressor and the increase detected only at times when CO would not be expressed in wild type. Does this have relevance for the wild type situation shown in for example Figure 5B? There the increase in FT mRNA is at 8-16 hours after dawn when no difference in CO protein is found at high temperature in 5D, so can we relate Figure 5D and 5B? Some explanation for these apparent discrepancies is required.

2. The experiments shown here were carried out in long days, whereas those of Kumar (2012) were done in short days. Here a temperature response is still detected in pif4 pif5 mutants, whereas Kumar found it was greatly reduced or absent in pif4 mutants. The authors assume this difference is because the two groups analyzed the effect of temperature in different day lengths, but would it not be useful to repeat the experiment of Kumar in short days? Perhaps the difference in results has another explanation that is not to do with differences in day length. This would seem an essential control.

3. Recently two papers appeared showing that at high temperatures the floral repressor SVP is reduced in abundance and activity (Pose et al Nature 2013; Lee et al Science 2013). These papers likely appeared when this manuscript was near submission but they do have direct relevance for the results and model shown here. SVP is a direct repressor of FT transcription. Therefore in long days as used here, FT expression would be expected to rise at high temperature due to reduction in SVP activity even if photoperiod pathway activity through CO did not increase at high temperature. Such a conclusion makes the effect of high temperature on CO activity shown in Figure 7 and the implicit assumption here that CO activity is increased at high temperature in long days not required to explain the data that FT mRNA rises in high temperature. The experiment shown in Figure 5D is therefore particularly important as it is the only evidence that photoperiod pathway activity rises at

high temperature in long days, and emphasizes the discrepancy mentioned in 1 and the need to repeat the analysis in plants expressing CO from the wild-type promoter.

4. Figure 1. The ChIP shown in Figure 1 is not clearly described in the legend and in parts did not seem very convincing. The conclusion of a positive ChIP seems to be based on comparing the CBS promoter element with the exon. However in the case of PIF5 this is a concern because the percentage of input pulled down appears lower than for actin but they conclude a positive result for PIF5. Is the standard error based on technical replicates for the PCR? If so how often were biological replicates performed? This should be stated in the legend. There is no "no antibody" negative control. What time of day was the experiment done? This should be stated in the legend. If it was done at dawn when CCA1 is expressed, then a good negative control used previously is to also to test for ChIP at the out of phase time of CCA1 expression when the signal should be low.

5. Figure 3. Why was CDF1ox rather than WT used to test and model the effect of gi on CDF1? Would data obtained in the wild-type not be more accurate for modelling?

6. Figure 3C seems to contain data from different previously published sources. Would it not be better to repeat these assays all in one condition? Similarly, although D legend says "as C", it's not clear where the data came from for D as the data in C came from different sources. The y-axis on 3c is labelled Relative Expression Level, but no value reaches 1 and it is a combination of data from different sources, so how the values were calculated was not clear. Perhaps this should be explained in the legend.

7. The increase in CO mRNA in the elf3 mutant in Figure 3C is used to support the idea that ELF3 stabilizes CDF1 protein (Figure 3A). However ELF3 could regulate CO mRNA in other ways. Would it be worth testing in a cdf1 elf3 double mutant, whether the increase in CO mRNA in the presence of elf3 is abolished?

8. In figure 1 PIF4 and PIF5 are proposed to be activated by CCA1 and LHY. Perhaps testing PIF4 and PIF5 mRNAs in the cca1 lhy double mutant in Figure 4 would be worthwhile.

Reviewer #2:

The authors extend (and clearly improve) a previous model for the circadian clock of Arabidopsis including the photoperiodic flowering pathway. They also add the hypocotyl elongation pathway and combine both pathways.

The authors test different hypotheses such as the recently proposed hypothesis of a warm T{degree sign}-induced flowering mechanism. They show that ´ wrong ª results can also lead to the discovery of some missing elements such as ELF3 as a regulator of CDF1 protein or CCA1 and LHY as regulators of CO and FT transcription. They also show that the model gives rise to some interesting counterintuitive predictions, e.g. about the PIF activity.

The manuscript represents an impressive amount of work and the authors not only refer to many previous experimental publications but also perform their own experiments. The combined experimental and theoretical approach gives to this work a clear high value. The manuscript is also very dense, contains a lot of information, and frequently refers to previous work (Pokhilko et al 2012 ; Salazar et al., 2009, ...).

Although I do not have any major problem with the manuscript, several clarifications/explanations could be useful and lead to a better understanding of the results :
- It is not always clear to understand why some elements are depicted by their mRNAs only (e.g. FT, ATHB2), other by their proteins only (e.g. NP, COP1) and some by both their mRNA and protein (e.g. CDF1, FKF1).
- It is sometimes difficult to understand what ´ hides ª behind the regulations : e.g. an inhibition of a protein could be due to a decrease of its production or to an increase of its degradation.
- When reading the main manuscript it is not clear why the authors incorporate COP1 ? It is explained in the Supplementary information but some explanation could also be given in the main text since COP1 appears in Figure 1.

- (p6) "Short days (SD) are described by 8L:16D or 10L:14D." This is confusing : what is the photoperiod used for the simulations or in the experimental data ?

The authors could also clarified the following points in the main text and in the Supplementary Information (SI) :
- (p4), ´ the circadian clock regulates ... PIF4 and PIF5 ª : The authors could be more specific about this regulation and explicitly mention which element of the circadian clock is regulating PIF4/5 ?
- (p5), ´ The resulting PIF proteins ... forming homo- and hetero-dimers ª : Since this information is missing in the scheme of Figure 1, the authors could explained why they do not take it into account.
- (p6), ´ Data analysis suggests that FKF1 transcription is regulated ... ª : some additional explanations could be interesting or useful about this ´ data analysis ª.
- (p15), ´ By using empirical equations relating FT and ATHB2 mRNA levels to flowering and hypocotyl elongation ª : The authors could clarify what they mean by ´ empirical equations ª ? Some explanations could be useful here.
- (SI, p10), ´ The parameter represents the time-advance of the EC profile ... ª : The authors introduce here a parameter ´ ª for the phase of the EC. Does it mean that the phase of the EC complex in the original model is ´ wrong ª ? Is a ´ simple ª change of parameter values (instead of introducing a time delay) not sufficient to fix the problem of the phase ?
- (SI, p11) ´ in order to simplify the model PIF4 and PIF5 ª the model is complex and includes many variables/parameters and it is not clear why the authors choose to simplify this particular pathway. Is it really an important simplification for the model ? Why not also simplifying the mRNAs ?
- (SI, p13) ´ To simulate 27{degree sign}C conditions, ... ª : The effect of T{degree sign} is not very clear and could be improved : e.g. why a 5{degree sign}C increase (from 22{degree sign} to 27 {degree sign}C) is simulated through a 10-fold increased for CO regulation of FT transcription. Why is it then written g15=g15/10 ? Why do the authors also mentioned an alternative scenario through parameter g13 ?
- (SI, p14) ´ the parameter d0 has the same value as in Salazar et al., 2009, whilst parameters a1, a2, a3, a4 and b were optimised by comparing the functions to hypocotyl length and flowering time data from across different photoperiods ª : Equation 19 is similar to Salazar et al., 2009 and it is not clear then why the parameters (besides d0) are so different : -15.29 vs -2308.141 for a4, 1.63 vs 0.02 for b.
- (SI, p22) ´ This analysis highlighted a small subset of parameters ... ª : The section ´ sensitivity analysis ª is interesting and could probably be described in more details. First, the authors mentioned a ´ small subset of parameters ª that are quite sensitive but a more detailed description of all these parameters could be useful. Second, the authors tested a 10% variation leading for some parameters to a maximum 30% increase. What happens for larger increases ? Are there still oscillations ?

Here are also some remarks about Supplementary Table 4 :
- This Table should be explicitly mentioned in the main text or in the supplementary information.
- Why are parameters linked to the translational rates in equations for the hypocotyl elongation so high compared to the flowering pathways (compare parameters p11-p16 with p1-p6). Does it mean that something is missing in the model and is ´ compensated ª by these high values ?
- The authors mentioned (SI, p11) a parameter . This parameter should also appear in this Table.
- At the end of the table, what are parameters a, b, ... h ?
- There are also two parameters ´ b ª, perhaps one of them could have another name.
- It could be useful to have not only the names of the parameters (with letters a, b, ...) but also a small description (rates of transcription of parameter a, ...).
- The units of ´ d0 ª should be ´ days ª (cfr Salazar et 2009).

Re-submission 15 September 2014

We are grateful to the reviewers for their detailed, positive, and constructive comments. We are happy that the reviewers agree that our results are “interesting”, and represent an “impressive amount of work… of clear high value”.
Here, we detail our responses to the comments of both reviewers to our previous submission, with our response in blue, and text quoted from the paper in green. We have also substantially extended the paper with new data, analysis, and modelling; we outline these separately at the end.

Response to reviewer #1

1. Figure 5D. The increase in CO protein at high temperature in long days directly links photoperiodic signaling and high temperature response. However in this figure CO protein is increased at high temperature between dawn and 8h. This difference is found in a CO overexpressor and the increase detected only at times when CO would not be expressed in wild type. Does this have relevance for the wild type situation shown in for example Figure 5B? There the increase in FT mRNA is at 8-16 hours after dawn when no difference in CO protein is found at high temperature in 5D, so can we relate Figure 5D and 5B? Some explanation for these apparent discrepancies is required.
To address this point, we performed further experiments (new Figure 5G), measuring CO protein levels when expressed from its endogenous promoter at both temperatures. From this, we conclude that any role for CO in temperature sensitivity is unlikely to arise from regulation of protein stability. Instead, it seems likely that factors other than CO and PIF4 are involved, possibly including SVP and FLM (see Point 3, below).

2. The experiments shown here were carried out in long days, whereas those of Kumar (2012) were done in short days. Here a temperature response is still detected in pif4 pif5 mutants, whereas Kumar found it was greatly reduced or absent in pif4 mutants. The authors assume this difference is because the two groups analyzed the effect of temperature in different day lengths, but would it not be useful to repeat the experiment of Kumar in short days? Perhaps the difference in results has another explanation that is not to do with differences in day length. This would seem an essential control.
We have now performed an experiment to assess the temperature sensitivity of *FT* expression in short days in the WT and the *pif4* mutant, as in Kumar *et al*., 2012. We see temperature sensitivity in *FT* levels in short days in both the WT and in the *pif4* mutant (new Supplementary Fig 17). This is discussed (page 15):
"The pif4;pif5 mutant clearly retained temperature sensitivity of FT expression in LDs (Fig 5H). Our data collected in SDs also showed temperature sensitivity of FT expression in the pif4 mutant (Supplementary Fig 17). This is consistent with previous reports (Kumar et al., 2012; Thines et al., 2014), though we did not detect a reduction in peak FT levels in the pif4 mutant. This suggests that additional, temperature-sensitive regulators of FT play a role in these conditions."

3. Recently two papers appeared showing that at high temperatures the floral repressor SVP is reduced in abundance and activity (Pose et al Nature 2013; Lee et al Science 2013). These papers likely appeared when this manuscript was near submission but they do have direct relevance for the results and model shown here. SVP is a direct repressor of FT transcription. Therefore in long days as used here, FT expression would be expected to rise at high temperature due to reduction in SVP activity even if photoperiod pathway activity through CO did not increase at high temperature. Such a conclusion makes the effect of high temperature on CO activity shown in Figure 7 and the implicit assumption here that CO activity is increased at high temperature in long days not required to explain the data that FT mRNA rises in high temperature. The experiment shown in Figure 5D is therefore particularly important as it is the only evidence that photoperiod pathway activity rises at high temperature in long days, and emphasizes the discrepancy mentioned in 1 and the need to repeat the analysis in plants expressing CO from the wild-type promoter.

The role of additional temperature regulators is now included in the model (new Figure 5). We acknowledge that additional factors are likely to play a role in the effects we see, and are now more circumspect in our discussion of temperature regulation of *FT* (page 15):
"Recently, it has been shown that FLOWERING LOCUS M (FLM) and SHORT VEGETATIVE PHASE (SVP) are involved in mediating the temperature sensitivity of FT expression in the temperature range 5°C to 27°C, and in both SDs and LDs (Pose et al., 2013; Lee et al., 2013). However, no single, dominant component was identified, as mutants in each of these factors retain some temperature sensitivity (Lee et al., 2013). The action of these regulators can be modelled by introducing a uniform activation of FT expression at 27°C. With this simple assumption, the model is able to reproduce the observed change in FT dynamics at 27°C (Fig 5I)."

4. Figure 1. The ChIP shown in Figure 1 is not clearly described in the legend and in parts did not seem very convincing. The conclusion of a positive ChIP seems to be based on comparing the CBS promoter element with the exon. However in the case of PIF5 this is a concern because the

percentage of input pulled down appears lower than for actin but they conclude a positive result for PIF5. Is the standard error based on technical replicates for the PCR? If so how often were biological replicates performed? This should be stated in the legend. There is no "no antibody" negative control. What time of day was the experiment done? This should be stated in the legend. If it was done at dawn when CCA1 is expressed, then a good negative control used previously is to also to test for ChIP at the out of phase time of CCA1 expression when the signal should be low.

We have removed the regulation of *PIF4* and *PIF5* transcription by CCA1 from the model, and acknowledge that the conclusion that CCA1 binds to the PIF5 promoter was not strongly supported by the data. The only significant consequence of this for the model is that simulated *PIF4* and *PIF5* transcription is now arrhythmic in the *elf3* mutant (new Supplementary Fig 9). These results, along with the motivation for testing the PIF4 and PIF5 promoters for CCA1 binding, are now discussed: "Residual rhythms of PIF4 and PIF5 in the elf3 mutant suggested a role for additional circadian regulators of PIF4 and PIF5 transcription. In particular, a small daytime peak in expression of PIF4 and PIF5 has been observed in multiple experiments (Lu et al., 2012; Nomoto et al., 2012b), and EE motifs are present in the PIF4 and PIF5 promoters. This suggested a possible role for CCA1 and LHY in activating PIF4 and PIF5 expression. However, CCA1 ChIP experiments did not provide strong evidence to support this hypothesis (Supplementary Fig 3). "
The presentation of the ChIP data now focusses on the binding of CCA1 to the promoters of CDF1 and FKF1, for which the data are strong and consistent across both biological replicates. We have also provided details of the experiment in the legend.
5. Figure 3. Why was CDF1ox rather than WT used to test and model the effect of gi on CDF1? Would data obtained in the wild-type not be more accurate for modelling?
Using a constitutive overexpressor allowed us to disentangle the effects of transcriptional from post-transcriptional regulation, and it's comparable to the data from CDF1ox;fkf1. The CDF1 data from CDF1ox;fkf1 and CDF1ox;gi lines are now presented alongside one another (Fig 3B,C) to highlight this comparison.

6. Figure 3C seems to contain data from different previously published sources. Would it not be better to repeat these assays all in one condition? Similarly, although D legend says "as C", it's not clear where the data came from for D as the data in C came from different sources. The y-axis on 3c is labelled Relative Expression Level, but no value reaches 1 and it is a combination of data from different sources, so how the values were calculated was not clear. Perhaps this should be explained in the legend.

In order to make it clear which experimental data are quantitatively comparable to one another, all figure panels now only contain data originating from the same experiment, which are therefore comparable.

7. The increase in CO mRNA in the elf3 mutant in Figure 3C is used to support the idea that ELF3 stabilizes CDF1 protein (Figure 3A). However ELF3 could regulate CO mRNA in other ways. Would it be worth testing in a cdf1 elf3 double mutant, whether the increase in CO mRNA in the presence of elf3 is abolished?

We now focus in our discussion of these observations on the idea that GI must act on CDF1 through an FKF1-independent mechanism. This is supported by the observed increase in CDF1 stability and decrease in *CO* transcript levels in the *gi* mutant compared to the *fkf1* mutant. How GI undertakes this regulation is an open question – involvement of ELF3 is one possibility that we put forward (page 16):
"The role of GI-FKF1 in controlling CO and CDF1 stability to control FT expression is well established. However, two lines of evidence suggest a further role for GI in the regulation of CDF1 stability. First, our measurements of CDF1 protein in a CDF1ox/gi line suggest that CDF1 is more stable in the absence of gi than in the absence of fkf1. Second, the decrease in CO expression in the gi mutant as compared to the fkf1 mutant (Fig 3D,E). The independent regulation of CDF1 by GI may be direct, for example with GI acting in complex with ZTL, and/or LKP2 (Ito et al., 2012), or indirect, as a result of GI's widespread regulation of other pathways. For example, GI is known to act antagonistically with ELF3 (Yu et al., 2008; Pokhilko et al., 2012)."
8. In figure 1 PIF4 and PIF5 are proposed to be activated by CCA1 and LHY. Perhaps testing PIF4 and PIF5 mRNAs in the cca1 lhy double mutant in Figure 4 would be worthwhile.
As discussed in Point 4 (above), we have removed the regulation of *PIF4* and *PIF5* by CCA1/LHY.

Response to reviewer #2

- It is not always clear to understand why some elements are depicted by their mRNAs only (e.g. FT, ATHB2), other by their proteins only (e.g. NP, COP1) and some by both their mRNA and protein (e.g. CDF1, FKF1).
We have clarified the motivation for modelling FT and ATHB2 mRNA as outputs of the network ("Linking molecular regulation to flowering time and hypocotyl elongation", page 13):
"ATHB2 expression provides a molecular correlate of hypocotyl elongation across multiple conditions (Kunihiro et al., 2011), in a similar manner to FT expression in the flowering pathway (Salazar et al., 2009)."

- It is sometimes difficult to understand what « hides » behind the regulations : e.g. an inhibition of a protein could be due to a decrease of its production or to an increase of its degradation.
In cases where the mechanism of regulation is understood, we have described this with reference to the literature (e.g. the phyB-PIF and FKF1-CO interactions). However, in some cases it is not clear how a regulatory effect is achieved.

- When reading the main manuscript it is not clear why the authors incorporate COP1 ? It is explained in the Supplementary information but some explanation could also be given in the main text since COP1 appears in Figure 1.
We now refer to this in the main text (page 8):
"Having connected the clock model to a model of CO/FT regulation and incorporated COP1 as a light input to this system (see Supplementary Information), we then compared model simulations and data for CO and FT mRNA from plants with mutations to clock genes."
However, we have deferred an in-depth explanation to the supplementary information due to space limitations.

- (p6) "Short days (SD) are described by 8L:16D or 10L:14D." This is confusing : what is the photoperiod used for the simulations or in the experimental data ?

The precise photoperiods in which data was obtained and the simulations were performed are specified in the figures by the light:dark bars in the standard manner. Rather than repeatedly specify the precise duration of the photoperiod in each case throughout the paper, we felt that the conceptual clarity of referring simply to short days and long days makes the paper significantly more readable overall.

The authors could also clarified the following points in the main text and in the Supplementary Information (SI) :
- (p4), « the circadian clock regulates ... PIF4 and PIF5 » : The authors could be more specific about this regulation and explicitly mention which element of the circadian clock is regulating PIF4/5 ?
This is now specified (page 10):
"The rhythmic transcription of PIF4 and PIF5 has been shown to be regulated by direct inhibition by the EC (FigureFig 4A; Nozue et al., 2007; Nusinow et al., 2011)."

- (p5), « The resulting PIF proteins ... forming homo- and hetero-dimers » : Since this information is missing in the scheme of Figure 1, the authors could explained why they do not take it into account.
The modifications to the model mean that this comment is no longer relevant.

- (p6), « Data analysis suggests that FKF1 transcription is regulated ... » : some additional explanations could be interesting or useful about this « data analysis ».
This line of reasoning is now explained in greater detail (page 6):
"We determined potential mechanisms by which the circadian clock might regulate FKF1 and CDF1 mRNA by inspection of published datasets (Mizoguchi et al., 2005; Niwa et al., 2007; Ito et al., 2008; Edwards et al., 2010). From these, we observed that FKF1 mRNA peaks at a similar phase to GI transcription across multiple photoperiods, whilst both respond in a similar manner to perturbations of the circadian clock. Under 10L:14D and 16L:8D cycles (throughout we shall refer to 8L:16D or 10L:14D as SD, and 16L:8D as LD) the peak of FKF1 expression at ZT9-10 matches that of GI (ZT = zeitgeber time, where dawn in an L:D cycle is at ZT0). FKF1 and GI expression both have an earlier peak phase in cca1;lhy mutants, whilst they exhibit only minor phase changes in

prr mutants (Figure 2D; Imaizumi et al. 2003; Niwa et al., 2007; Ito et al., 2008). Furthermore, FKF1 transcription, like that of GI, is acutely stimulated by red light (Tepperman et al., 2004; Locke et al., 2005)."

- (p15), « By using empirical equations relating FT and ATHB2 mRNA levels to flowering and hypocotyl elongation » : The authors could clarify what they mean by « empirical equations » ? Some explanations could be useful here.
We are now more explicit about the motivation for this approach:
"The model simulates the nonlinear changes in average ATHB2 mRNA and FT mRNA levels across photoperiods (FigureFig 6A & B). The absolute values of hypocotyl length and flowering time vary among laboratories, so we used simple mathematical functions to relate FT and ATHB2 mRNA levels to flowering and hypocotyl elongation. These represent the complex developmental mechanisms of the vegetative-to-inflorescence transition and the biophysics of elongation growth, and can readily be recalibrated for the conditions of particular studies (as described in Supplementary Information)."

- (SI, p10), « The parameter τ represents the time-advance of the EC profile ... » : The authors introduce here a parameter « τ » for the phase of the EC. Does it mean that the phase of the EC complex in the original model is « wrong » ? Is a « simple » change of parameter values (instead of introducing a time delay) not sufficient to fix the problem of the phase ?
We found that there was no simple change of parameters that would allow us to modify the EC phase without having knock-on effects on the behaviour of the circadian clock, which is not the focus of this paper (we note that both Reviewer 1 and 2 highlighted that the paper already covers a "wide range of topics" and "is… very dense [and] contains a lot of information"). We note this apparent discrepancy, but addressing this issue was beyond the scope of this paper.

- (SI, p11) « in order to simplify the model PIF4 and PIF5 » the model is complex and includes many variables/parameters and it is not clear why the authors choose to simplify this particular pathway. Is it really an important simplification for the model ? Why not also simplifying the mRNAs ?
We have simplified in order to guard against overfitting of the model. Additional simplification has been undertaken in this revision. The model of CO regulation also includes judicious simplifications – the additional complexity in the case of the CO model is because much more experimental evidence exists for the molecular interactions between the components in the CO pathway, and data are available for both transcript and protein levels.

- (SI, p13) « To simulate 27{degree sign}C conditions, ... » : The effect of T{degree sign} is not very clear and could be improved : e.g. why a 5{degree sign}C increase (from 22{degree sign} to 27 {degree sign}C) is simulated through a 10-fold increased for CO regulation of FT transcription. Why is it then written g15=g15/10 ? Why do the authors also mentioned an alternative scenario through parameter g13 ?
The temperature regulation has been modified in the present model. Temperature now enters the regulation of FT through modification of the transcription rates n12 and n13 (see equation 23).

- (SI, p14) « the parameter d0 has the same value as in Salazar et al., 2009, whilst parameters a1, a2, a3, a4 and b were optimised by comparing the functions to hypocotyl length and flowering time data from across different photoperiods » : Equation 19 is similar to Salazar et al., 2009 and it is not clear then why the parameters (besides d0) are so different : -15.29 vs -2308.141 for a4, 1.63 vs 0.02 for b.
The equation parameters have been reoptimised, and take very different values because of compensation between different parameters in the equation. As we explained in Salazar *et al.*, 2009, this equation takes a simple mathematical form in which parameters are not directly related to biological mechanisms. The parameters are therefore not comparable to one another in a meaningful way, and different parameter sets may in fact result in the same behaviour.

- (SI, p22) « This analysis highlighted a small subset of parameters ... » : The section « sensitivity analysis » is interesting and could probably be described in more details. First, the authors mentioned a « small subset of parameters » that are quite sensitive but a more detailed description of all these parameters could be useful. Second, the authors tested a 10% variation leading for some

parameters to a maximum 30% increase. What happens for larger increases ? Are there still oscillations ?
We have adjusted the way in which parameter sensitivity is presented, and now discuss the most sensitive parameter (the other parameters were generally not sensitive) (see Supplementary Information, page 24):
"Notably, altering the COP1- and dark-dependent degradation of CO protein (parameter m6) had a large effect on FTAREA (Supplementary Figure 19). This can be explained by a combination of two effects. First, it is clear that reducing the degradation rate of CO protein will lead to an increase in CO protein levels, and therefore an increase in FT levels. Second, this reduction in degradation rate also extends the time window during which CO protein is present, coinciding with the later ZTs during which more CO transcript is present."

Here are also some remarks about Supplementary Table 4 :
- This Table should be explicitly mentioned in the main text or in the supplementary information.
This is now referred to under "Parameter optimization and simulation tools", Supplementary Information, page 7 (note: it is now Supp Table 6).

- Why are parameters linked to the translational rates in equations for the hypocotyl elongation so high compared to the flowering pathways (compare parameters p11-p16 with p1-p6). Does it mean that something is missing in the model and is « compensated » by these high values ?
We believe that some such combinations of parameters arise simply as a result of compensated changes in different parts of the model, and may be affected by the initial guess of the parameter values. For example, if the rate of translation of a transcription factor and its affinity for a target are changed in opposite directions, the same model behaviour may be acquired. The lack of 'true' dimensionality in the data (i.e. the fact that we only have information on relative concentrations) means that such differences in parameter values cannot yet be interpreted in a biologically meaningful way.

- The authors mentioned (SI, p11) a parameter τ. This parameter should also appear in this Table.
- At the end of the table, what are parameters a, b, ... h ?
- There are also two parameters « b », perhaps one of them could have another name.
- It could be useful to have not only the names of the parameters (with letters a, b, ...) but also a small description (rates of transcription of parameter a, ...).
- The units of « d0 » should be « days » (cfr Salazar et 2009).
The table has been updated appropriately and parameters referred to and renamed where necessary (note that the refactoring of the PIF model means that parameter names are not comparable between this model and the previous version).

New modelling and analysis

Identification of PIF targets through analysis of microarray datasets
We have applied clustering analysis to publically available microarray datasets from the DIURNAL database in a number of light conditions and under different genetic perturbations to identify sets of transcripts with dynamics that are consistent with PIF regulation in light:dark cycles. With this approach, we identify 191 PIF-induced and 209 PIF-repressed transcripts as PIF targets in light:dark cycles. Additionally, we were able to show that the model is consistent with PIF regulation of these transcripts (new Fig 4, new Supplementary Figs 9,10,20), and have extended the model to describe the dynamics of PIF-repressed transcripts in light:dark cycles (new Supplementary Fig 14):
"Recent experiments with dark-grown seedlings have identified an expansive transcriptional network downstream of the PIFs (Zhang et al., 2013), with 699 genes identified by RNA-Seq as having decreased transcript levels in pif1;4;5 mutants, while 755 genes were identified as having increased levels in pif1;4;5 mutants. We refer to these sets as PIF-induced and PIF-repressed, respectively. In order to evaluate whether our model of PIF activity could describe the dynamics of PIF targets other than ATHB2 and IAA29 in light:dark cycles, we used microarray timeseries data available from the DIURNAL database (Mockler et al., 2007). This database includes microarray data sampled across two days at 4h time resolution in diverse conditions, and has previously been used to assess interactions between circadian and light signalling (Dalchau et al., 2010). Clustering

of transcript dynamics for genes identified as PIF-induced revealed two large and coherent clusters of genes; genes within each cluster shared condition-specific transcript dynamics across 6 conditions, including SDs and LDs (see Supplementary Information for details of analysis; Frey and Dueck, 2007). These genes comprised 191 of the 699 PIF-induced genes, including the known examples of ATHB2 and IAA29, and showed significant overlap with PIF4,5-bound genes, relative to all PIF-induced genes (p<10-8, Supplementary Fig 12A). Two additional PIF target species were introduced into the model to represent these two clusters. Their dynamics could be matched in a straightforward way by fitting only 5 PIF target-specific parameters, as shown in Fig 4I-L for the comparison of SDs to LDs (microarray data from Michael et al., 2008b). Many of these genes also showed the SD-specific bimodal profile.

The generality of this model of PIF target regulation was further tested by considering genetic perturbations. Two classes of genetic perturbation are of particular interest – mutants with a defective EC (i.e. with the clock regulator of PIF transcription removed) and mutants with a defective circadian clock. In all cases, the model matched available datasets."

Further evidence for PIF activity during the day in diurnal conditions

In the previous submission, we made the hypothesis that PIF protein is present during the day based on observations of *FT* expression and the redundancy between phyB- and 'Interactor'-mediated suppression of PIF activity. Here, we are able to provide additional support for this hypothesis in two ways. First, by inspection of PIF target dynamics from plants grown in constant light (new Supplementary Figure 15) (page 12):

"…we inspected the dynamics of the clusters of PIF targets (as identified previously – see above; Supplementary Information) in constant light (LL) conditions under clock-entraining temperature cycles. Clear rhythms of PIF targets in these conditions are observable, with PIF-induced and PIF-repressed transcripts in phase and antiphase, respectively, with the phase of the PIF4 and PIF5 transcript rhythms (Supplementary Fig 15)."

Second, by inspection of PIF target dynamics in the *phyB* mutant (new Supplementary Fig 16) (page 13):

"If the degradation of PIF proteins during the day is not required for the observed dynamics of PIF activity in light:dark cycles, then removal of phyB should not affect the dynamics of PIF targets in these conditions. We assessed this possibility by inspecting the dynamics of the clusters of PIF targets (as identified previously – see above) in the phyB mutant (Supplementary Fig 16). The qualitative dynamics of both PIF-induced and PIF-repressed targets are unchanged in the phyB mutant"

Extension of the PIF model to include temperature regulation of EC binding to the PIF4 promoter

In the previous submission, we focussed on temperature regulation of the flowering pathway, since we lacked a mechanism for temperature regulation of the hypocotyl pathway. Recently, it has been shown that EC repression of *PIF4* expression is relieved at higher temperatures (e.g. 28°C vs 22°C), leading to higher *PIF4* levels during the night in these conditions (Mizuno *et al.*, 2014). This is now incorporated into the model, and allows us to more completely evaluate crosstalk between the hypocotyl and flowering pathways (new Figure 5) (page 14):

"It has been suggested that PIF4 plays a role in the temperature sensitivity of hypocotyl growth through stimulation of ATHB2, IAA29, and other hormone-related genes (Koini et al., 2011; Nomoto et al., 2012a), and in the temperature sensitivity of flowering through stimulation of FT (Kumar et al., 2012). The present model allows us to assess how PIF4 may achieve this combined regulation (Fig 5). Recently, it has been shown that EC repression of PIF4 expression is relieved at higher temperatures (e.g. 28°C vs 22°C), leading to higher PIF4 levels during the night in these conditions. The model reproduced these observations through mild temperature modulation of the affinity of the EC for the PIF4 promoter, resulting in less EC repression of PIF4 expression at the higher temperature (Fig 5 B-E). Altered affinity is sufficient to prevent full repression of PIF4 mRNA in the early night and to allow a 2-3h earlier rise of PIF4 before dawn at 28°C; thus warm temperature in LDs leads to night-time levels of PIF4 mRNA that are similar to the level in SDs at 22°C. In both model and data, this results in in 3-4-fold higher accumulation of ATHB2 at dawn, qualitatively consistent with the increased hypocotyl elongation observed at the higher temperature (Nomoto et al., 2012b)."

© European Molecular Biology Organization

2nd Editorial Decision 29 October 2014

Thank you again for submitting your work to Molecular Systems Biology. We have now heard back from the three referees who accepted to evaluate the study. As you will see, the referees find the topic of your study of potential interest and are in general supportive. They raise however a series of issues and make suggestions for modifications, which we would ask you to carefully address in a revision of the present work. The recommendations provided by the reviewers are very clear in this regard.

We also kindly ask you to include as 'dataset' files the data that are new to this study and refer to them accordingly in the text and Supp Table S4. With regard to the computational models, we greatly appreciate that you provide the annotated Matlab scripts. We would however also ask you to provide these models as SBML files (as 'dataset' files), deposit the models in BioModels and include the respective accession numbers.

With regard to the data re-used in this study, given that you made considerable efforts collecting the data from an impressive number of previous publications, I wonder whether it would not be worth collecting these datasets in a compendium dataset (ie as a zip file, with a README file at the top level specifying the source of each dataset as specified in Supp Table 4) so that readers can easily see the actual data used in the study.

Thank you for submitting this paper to Molecular Systems Biology.

Reviewer #1:

In this work, the authors extend their previous model of the circadian clock by linking it to two clock-regulated pathways, PIF-driven hypocotyl elongation and flowering time. More specifically, they combine their previous model with other known processes and a few specific hypothesis and show that their model can qualitatively match expression time courses of various genes in various genetic background in short or long days light conditions.

According to my understanding, their new claims in this paper are the following:

1. FKF1 is negatively regulated by both the EC and CCA/LHY
2. CDF1 is negatively regulated by the PRRs and positively regulated by CCA/LHY
3. GI also acts independently from FKF1 in reducing CDF1 protein stability
4. PIFs retain some activity during the day

Points 1 and 2 are in part validated by ChIP data, but the point is rather that the model as a whole can recapitulate many, but of course not all, observed features of the regulation.

This work is a serious attempt to make sense of large amounts of data and put some order into many previous studies that have focused on particular aspects of this regulation. Previous versions of this model have raised broad interest from quantitative biologists working in this field and this extended version should be no exception.

The challenge with such a large model is to characterize to what extent it reflects the reality. The authors often justify their models by showing that it "qualitatively matches" the observed time courses on which the model was trained and sometimes also predicts features of new times courses. Those qualitative features can be at times relatively trivial, so it is difficult to judge the performance of a model. A useful information could be provided by using negative control models, for example by considering relevant alternative models and see whether the one presented here better matches

the data.
For example, in Fig 4I-L, would it be possible to match the cluster exemplar as well if one assumed that they would be directly controlled by the circadian clock (for example EC)? What is the percentage of (PIF induced, or any) genes that can be matched as well or better than the exemplars?

A model must ultimately be judged by the insights it provides. This is also difficult to evaluate here as one cannot really distinguish between core features of the model and its artifacts that are dependent on specific choices of parameters. For example, in page 10, the authors mention a "secondary peak" at ZT14 in the simulated PIF activity in SD, and that this secondary peak has been observed in other studies. This peak is not very salient in the model, so it could well be a parameter-dependent artifact.

To me, the interest of this paper lies less in any particular biological insight that it provides than in the formalization of the interactions between the circadian clock and the two pathways mentioned above. I would recommend that the authors focus on a small number of non-trivial features for which the model provides explanations (such as the bimodal PIF activity), and show that those are robust features of that particular model (possibly stating what other kind of models could achieve such features). To achieve this, it would be needed to go beyond one model and one optimal parameter set and move in the direction of ensemble modeling or parameter distributions (see e.g., Vanlier et al. (2013), Mathematical Bioscience) and to infer significance statistics from permutation experiments, where the model is fitted to the "wrong" data to see if it can also model it.

Some minor points:

Sup Fig. 12: I could find how the set of PIF-bound genes was defined, making the interpretation of this result difficult
An aspect that this is in my opinion not discussed enough regards the initial state. How is it set, and what is it's influence on the simulations? Can it be shown that the whole model reaches a limit cycle?

Reviewer #3:

Open review from Seth Davis

Here the authors generate two models to connect outputs from diurnal signals to explain mathematically aspects of photoperiodic control of i) flowering time and ii) seedling-stem growth. There is first a refinement of the model generated in the 2012 Science paper on flowering time. Numerous gene expression patterns were measured as guided by that older model and these new parameters were used to refine the Song model. FKF1 transcription was inferred to be evening expressed in a smilier manner as GI. More novel, the expression of CDFs was explained, as the expectation that they would be solely controlled by PRRs was shown false, and the use of CCA1/LHY is proposed. Indeed CCA1 protein was found to associate to the chromatin of both FKF1 and CDF1 promoters. Next the role of FKF1 and GI was examined at the target CO. Interestingly, FKF1-dependent and FKF1-independent effects of GI on CDF1 protein stability were found. This was not necessarily connected to FT. But as it is a marker of flowering, that does not seem critical to the model. These were then connected to PRR9/7, CCA1 and ELF3. Effects on CDF1 and FKF1 correlated with biological responses. These models could be validated, and together, a refined understanding of the role of clock genes in driving photoperiodic modulators is improved. One example of the predictive power of this model is shown by the inability of the model to capture the behaviour of the cca1/lhy mutant, which means, that the new model predicts that there is still a missing element(s) to be discovered. That had not been previously expected. Next the paper jumps to PIF4/5 regulation to explain seedling growth. Clock control of PIF4/5 as modelled. From there, PIF4/5 control of auxin-related genes was modelled. PIF protein sterility was included in this model. Interestingly, combining the regulation of PIF protein activity by light with circadian regulation of PIF4 and PIF5 transcription was sufficient to describe the observed photoperiod response of ATHB2 and IAA29 mRNA accumulation. This was then connected to genome-wide studies and clustering of transcript accumulation patterns supported PIF4/5 control of ATHB2 and IAA29 (and other genes). The model was then tested by examination of RNA in genetic resource backgrounds.Clustering of transcripts was again performed. Here the model was not always suited.

© European Molecular Biology Organization

Again this supports the notion that missing factor(s) await to be discovered. One very interesting feature of the PF4/5 model was the supporting notion that these can work outside of the pre-dawn phase. That is quite novel. Importantly, next, the authors link the flowering time model to the auxin ideas from PIF4/5 with the description of ATHB2 control of FT expression. This is bridged to ambient temperature control of PFI4/5. That is also modelled. Experimental data supported the model findings that thermal inputs to the evening complex explain temperature effects. This connected back to flowering time and a prediction that PIF and CO coordinately regulate FT expression was experimentally supported.

This is an enormous amount of data. This paper will undoubtably be highlight cited, as it engages numerous disparate communities. If possible, the greatest benefit for manuscript improvement is an improved flow between thought lines. As written this reads as 2-4 papers stitched together. That is ok, but it could flow better if improved thought-line links could be found.

For the sentence, "The CCA1 & LHY proteins inhibit transcription of EVENING COMPLEX (EC) components, EARLY FLOWERING 4 (ELF4), ELF3 and LUX ARRYTHMO (LUX), delaying their accumulation until dusk." one key reference is missing. Lu et al. Plant Physiol. 2012 Feb;158(2):1079-88.

Most of the first paragraph of the results, from "Our previous model ..." to "... FKF1-dependent degradation of CDF1 (Song et al., 2012)", this is introduction and not a result. This paragraph need to be trimmed to start at "However, this model required FKF1 protein ..." and all other text needs to be moved to the introduction. Perhaps it is erred to be moved in a stream-lined way to the terminal introduction sentence to highlight why refined models are useful.

For the terminal paragraph of the introduction, it is not clear that two different models were made. I recommend a very small expansion to the sentence, "We demonstrate that known transcriptional and post-transcriptional regulation of PIF explains phenotypes and PIF target transcript dynamics under a variety of environmental and genetic manipulations." emphasising that this is different that the CDF1 / FKF1 model mentioned in the preceding sentence. Similarly, an expansion of the word "results" in the sentence "The results highlight that a complex network structure underlies circadian-, light- and temperature-regulated processes." could be improved to highlight that there are two sets of results.

There needs to be some kind of transition from the CDF1/FKF1 models to the PIF4/5 models, as as it reads now, it is a leap for the reader and there is no connection in the results. I like the first sentence in the PIF4/5 section, but I think something above in the CDF1/FKF1 section is needed as a bridge to finalise the connections.

For "The model including this regulation captures..." I recommend an expect statement of what model was made. More information of the intentions are needed to engage in this thought line. Maybe something like "Here we will describe the methods that led to the development of a model that described PFI4/5 expression and connected that to the physiology of photoperiodic induction of growth."

For "However, CCA1 ChIP experiments did not provide strong evidence to support this hypothesis." please be explicit that CCA1 binding to PIF4/5 does not match the hypothesis. As written the reader cannot understand why there is no strong evidence.

The paragraph "The generality of this model of PIF target regulation was further tested ..." starts well, but lacks an explanation of the findings. Then it lacks a brief conclusion sentence describing the implications of the testing of the model.

I cannot find grant BB/F59011/1 at the BBSRC website. Is this code correctly typed?

Errors exist in the references, such as:

"Bai MY, Fan M, Oh E, Wang ZY (2012b) A triple helix-loop-helix/basic helix-loop-helix cascade controls cell elongation downstream of multiple hormonal and environmental signaling pathways in Arabidopsis. Plant Cell doi: 10.1105/tpc.112.105163" has a volume and page as 24(12):4917-29

© European Molecular Biology Organization

Similarly fix, "Antagonistic basic helix-loop- helix/bZIP transcription factors form transcriptional modules that integrate light and reactive oxygen species signaling in Arabidopsis."

"Doyle MR, Davis SJ, Bastow RM, McWatters HG, Kozma-Bognar L, Nagy F, Millar AJ, Amasino RM (2002) The ELF4 gene controls circadian rhythms and flowering time in Arabidopsis thaliana. 419: 74-77." journal missing

Font problems in "Thines BC, Youn Y, Duarte MI, Harmon FG (2014) The time of day effects of warm temperature on flowering time involve PIF4 and PIF5. J Exp Bot. 65(4):1141-51"

For figure 2 and 3, supp 1, supp2 supp7, supp8, supp15 I strongly recommend the light blue and light green lines are put to stronger, easier to see colours. The light blue in particular is almost impossible to see and will poorly print.

Reviewer #4:

This paper represents an important advance in mathematical models of circadian phenomenon in plants. In previous work models had been developed to describe the behavior of the circadian oscillator itself. Separate models had been developed for control of flowering time (an important circadian output). However previous flowering time models did not incorporate the oscillator itself; they instead relied on measured oscillatory behaviors of the flowering time genes. This limited the ability of those models to be predictive for genetic or environmental perturbations of the clock.

The work here develops a new model for flowering time that overcomes the previous limitations by directly incorporating a model of the central oscillator and linking it to the relevant flowering time genes. This provide a much more complete and predictive model for flowering time. In addition, the authors develop a model for control of hypocotyl elongation that incorporates both the oscillator and key players that control rhythmic growth behaviors--the first of its kind to my knowledge.

The paper is well written, although somewhat dense (I don't think much can be done about that--there is a lot of information here). Conclusions are justified, explanations are clear. I have only minor comments that need to be addressed.

Minor comments:

p. 4. The first report that PIFs were degraded in the light came from Park (2004). Although Park studied PIF3 rather than PIF4 and 5 described here, the paper should still be cited.

p. 8. "with mutations to clock genes". I think this should read "with mutations IN clock genes".

p. 8. Not only does the model fail to capture the dynamics of CO and FT in the cca1;lhy1 double (discussed), it also fails to capture CO dynamics in the elf3 mutant (Sup Fig 7G--not discussed). This should be noted/briefly discussed.

Figure 4 Legend. SD and LD are swapped in the legend for 4B-G.

p. 9. The authors state: "In an elf3 background, the level of PIF4 transcripts is increased throughout the night...this behavior is matched by simulations (Sup 9A,D). This does not do justice to the differences between the data and the model. In the observed data there is an increase throughout the night, whereas the model has it constant. This is somewhat acknowledged a few sentences later "Residual rhythms..." but I think this section should be reworked. The same criticism is true of simulated ELF3 in the prr9;7;5 mutant.

p. 9. "...a small daytime peak in expression of PIF4 and PIF5...". Please clarify: do you mean in wild type or in elf3 mutants? I assume the latter but is not clear.

p. 11. In the middle paragraph ("The generality..."). the callouts to Sup. Fig. 9 and 10 are backwards.

© European Molecular Biology Organization

p. 11, same paragraph as previous comment. Interpretation of the results described in this paragraph should be given.

p. 13, first paragraph. It is great that the patterns are qualitatively the same. Please also discuss the quantitative differences that confirm an important role for light/phyB regulation of PIF targets.

p. 13-14. Figure 6 is called out before Figure 5.

p. 14-15. This section seems to be organized oddly. That is the fit of the model is described in detail first, and then the details of how the model was constructed (i.e. FLM and SVP) are discussed in a separate section. Consider reworking this.

1st Revision - authors' response 21 November 2014

We thank the reviewers for their time and constructive suggestions, which we reproduce for the reviewers' convenience and address in full below. Regarding the editorial points:

1. We have provided the data along the suggested lines. The data for our own experiments are included as separate files, with metadata specifying conditions and referencing the figure in which they are shown. All timeseries experimental data, both our own and the data we obtained from the literature, are included in a new supplementary spreadsheet, again with metadata specifying conditions, the original source (figure number and citation), and the figure in our paper. In addition, we note that many of the datasets used (including our own) are available, with detailed metadata, on the BioDare data repository (www.biodare.ed.ac.uk). A new supplementary table has been added listing the stable, public online identifiers for these data sets.

2. For the model, we regret that we have found it difficult to provide an SBML version, since the model includes a discrete phase advance of the EC component. This cannot be described in SBML. An alternative would be to describe it as a delay of the appropriate length (i.e. in standard conditions, a 22 h delay instead of 2 h advance). While this function can be described in valid SBML, we found no software tool that allowed us to create an SBML model including this function, and we further note that standard SBML tools (e.g. Copasi, SBSI, JigCell) are not able to simulate such models.

Reviewer #1:

….This work is a serious attempt to make sense of large amounts of data and put some order into many previous studies that have focused on particular aspects of this regulation. Previous versions of this model have raised broad interest from quantitative biologists working in this field and this extended version should be no exception.

We thank the reviewer for their summary and positive comments.

The challenge with such a large model is to characterize to what extent it reflects the reality. The authors often justify their models by showing that it "qualitatively matches" the observed time courses on which the model was trained and sometimes also predicts features of new times courses. Those qualitative features can be at times relatively trivial, so it is difficult to judge the performance of a model. A useful information could be provided by using negative control models, for example by considering relevant alternative models and see whether the one presented here better matches the data.
For example, in Fig 4I-L, would it be possible to match the cluster exemplar as well if one assumed that they would be directly controlled by the circadian clock (for example EC)? What is the percentage of (PIF induced, or any) genes that can be matched as well or better than the exemplars?

The reviewer is correct that it is often useful to illustrate the validity of a particular model by comparison with negative controls. However, we give separate, detailed consideration to each distinct subset of interactions. In this context, these negative controls are often trivial (e.g. *PIFs* without any circadian regulators; PIF activity not repressed by light), or are already dealt with implicitly. For example, a potential 'control' model of *CDF1* transcription that includes only repression by the PRRs is eliminated by the observed low level of *CDF1* transcription in the *cca1;lhy* mutant: PRR levels are reduced in this mutant, so *CDF1* transcription would increase in the mutant if it was only repressed by PRRs. As a result, we considered that the space that would be required to discuss such comparisons in each case was better spent justifying in detail the model structure with reference to the experimental literature.

In the particular case of whether cluster exemplars could equally be explained by an alternative circadian clock output, we note that no known circadian clock component has dynamics similar to the clusters of PIF targets. The underlying, related question of whether these clusters represent true targets of PIF regulation is answered in part by the consistency of their behaviour in datasets that did not inform the clustering (i.e. the constant light and *phyB* mutant datasets (Supplementary Figs 16 & 17)).

A model must ultimately be judged by the insights it provides. This is also difficult to evaluate here as one cannot really distinguish between core features of the model and its artifacts that are dependent on specific choices of parameters. For example, in page 10, the authors mention a "secondary peak" at ZT14 in the simulated PIF activity in SD, and that this secondary peak has been observed in other studies. This peak is not very salient in the model, so it could well be a parameter-dependent artifact.

To me, the interest of this paper lies less in any particular biological insight that it provides than in the formalization of the interactions between the circadian clock and the two pathways mentioned above. I would recommend that the authors focus on a small number of non-trivial features for which the model provides explanations (such as the bimodal PIF activity), and show that those are robust features of that particular model (possibly stating what other kind of models could achieve such features). To achieve this, it would be needed to go beyond one model and one optimal parameter set and move in the direction of ensemble modeling or parameter distributions (see e.g., Vanlier et al. (2013), Mathematical Bioscience) and to infer significance statistics from permutation experiments, where the model is fitted to the "wrong" data to see if it can also model it.

In response to the reviewer's comments, and since we believe the bimodal dynamics of PIF-induced transcripts in SDs is a model behaviour of particular interest, we investigated the robustness of this model behaviour by random perturbation of model parameters. The results of this are now presented in Supplementary Fig 12, and show that in many cases pronounced bimodal dynamics are observed. This demonstrates that the relatively mild bimodal dynamics observed in simulations of the particular case of *IAA29* referred to by the reviewer (Fig 4C) are not an extreme case that depends on a special choice of parameters. These results are now referenced in the main text (page 10):

"In the model, this behaviour is the result of high PIF4 and PIF5 transcript levels coinciding with darkness at ZT8-12 in SDs, resulting in an SD-specific increase in PIF activity at this time and a peak in target gene expression at ZT14. Analysis of model dynamics under random parameter perturbations confirmed that this behaviour is observed across a broad range of parameter values (Supplementary Fig 12; see Model Behaviour in Supplementary Information)."

And expanded upon in detail in the Supplementary Information (page 18):

"One of the interesting observations from model simulations was the SD-specific increase in PIF-induced transcript expression in the early night. This is seen in IAA29 (Fig 4C), and in several other PIF-induced transcripts (Nomoto et al., 2012b). A natural interpretation of this behaviour is that the presence of PIF4 and PIF5 transcript in the early night in SDs can lead to a secondary peak in PIF activity at this time. In order to assess how robust this feature of the model is to changes in parameters, we performed a random parameter perturbation experiment. Specifically, we perturbed the parameters g_{14}, g_9, n_{11}, p_{12}, p_{13}, m_{13}, m_{14}, p_{10}, p_{11}, and m_{12}. Changing these parameters changes

© European Molecular Biology Organization

the dynamics of PIF proteins (Eq 12), Interactor proteins (Eq 11), and a PIF-induced transcript (e.g. as in Eq 13) while leaving the dynamics of photoreceptors (Eqs 9 & 10) and PIF transcripts (Eqs 7 & 8) unchanged. Each parameter was perturbed according to:

$$p_{new} = 2^X p_{old} \qquad (30)$$

The random variable X was drawn from a standard normal distribution ($X \sim N(0,1)$), with the result that approximately 30% of parameters are expected to be perturbed by more than 2-fold overall. After perturbation of parameters, the resulting model was simulated in SD and LD conditions (Supplementary Fig 12). The diversity of PIF and Interactor dynamics observed in simulations demonstrates that these parameter perturbations were sufficient to strongly affect the model dynamics. Parameter sets in which the PIF-induced transcript dynamics departed qualitatively from known dynamics of PIF activity were then filtered out. This includes, for example, cases where the PIF-induced transcript was not expressed in either condition (such cases are expected given the magnitude of the parameter perturbations).

Parameter sets were classified in two stages according to the simulated dynamics of PIF-induced transcript. First, classification of dynamics as significantly bimodal in SDs was determined according to whether a secondary peak and subsequent trough was observed during the time window ZT12-18, and whether its peak-to-trough ratio was greater than 1.2. Of the remaining parameter sets, dynamics were classified as having a SD-specific increase in the early night if there was a greater than 3-fold induction (measured at maximal expression) in SDs than LDs during the time window ZT8-20. The remaining parameter sets displayed neither behaviour, and by process of elimination only displayed a SD-specific peak at ZT0. The ability of this procedure to distinguish between qualitatively distinct dynamics is apparent from the classification of 20 example parameter sets presented in Supplementary Fig 12."

Some minor points:

Sup Fig. 12: I could find how the set of PIF-bound genes was defined, making the interpretation of this result difficult

We are grateful to the reviewer for pointing out this gap and have clarified this in the figure legend.

An aspect that this is in my opinion not discussed enough regards the initial state. How is it set, and what is it's influence on the simulations? Can it be shown that the whole model reaches a limit cycle?

The reviewer is correct to point out that this requires clarification. We have added a remark to this effect in the Supplementary Information (page 2):

"All simulations were run in simulated light:dark cycles until successive days displayed identical dynamics, as done previously for the circadian clock model (Pokhilko et al., 2012)."

Reviewer #3:

Open review from Seth Davis

...This is an enormous amount of data. This paper will undoubtably be highlight cited, as it engages numerous disparate communities. If possible, the greatest benefit for manuscript improvement is an improved flow between thought lines. As written this reads as 2-4 papers stitched together. That is ok, but it could flow better if improved thought-line links could be found.

We thank the reviewer for their comments and for their detailed assessment of our work.

For the sentence, "The CCA1 & LHY proteins inhibit transcription of EVENING COMPLEX (EC) components, EARLY FLOWERING 4 (ELF4), ELF3 and LUX ARRYTHMO (LUX), delaying their accumulation until dusk." one key reference is missing. Lu et al. Plant Physiol. 2012 Feb;158(2):1079-88.

This reference has been inserted in this location.

Most of the first paragraph of the results, from "Our previous model ..." to "... FKF1-dependent degradation of CDF1 (Song et al., 2012)", this is introduction and not a result. This paragraph need to be trimmed to start at "However, this model required FKF1 protein ..." and all other text needs to be moved to the introduction. Perhaps it is erred to be moved in a stream-lined way to the terminal introduction sentence to highlight why refined models are useful.

The introduction has been reworked to include an overview of the previous model.

For the terminal paragraph of the introduction, it is not clear that two different models were made. I recommend a very small expansion to the sentence, "We demonstrate that known transcriptional and post-transcriptional regulation of PIF explains phenotypes and PIF target transcript dynamics under a variety of environmental and genetic manipulations." emphasising that this is different that the CDF1 / FKF1 model mentioned in the preceding sentence. Similarly, an expansion of the word "results" in the sentence "The results highlight that a complex network structure underlies circadian-, light- and temperature-regulated processes." could be improved to highlight that there are two sets of results.

The introduction now considers the two parts of the model separately. First, by giving an overview of the (Song et al, 2012) model of the flowering pathway (as discussed above), and then by separately discussing the PIF pathway and how the two are combined. With these combined changes, the introduction now finishes (page 5):

"In this study, we have constructed a mathematical model to integrate and reconcile the complex molecular mechanisms in the photoperiodic pathways of flowering and hypocotyl elongation in Arabidopsis. In the flowering pathway, we extended our previous model, which was built to determine how FKF1 protein regulates levels of FT mRNA through CDF1 and CO protein interactions (Song et al., 2012). This previous model was able to match CO and FT mRNA rhythmic expression data in different photoperiods and in mutants of the flowering pathway (e.g. fkf1, gi, cdf1, CO-ox, CO-ox;fkf1, CO-ox;CDF1-ox). However, this model required FKF1 protein and CDF1 mRNA timeseries data to be input into the model, meaning that simulation of multiple photoperiods and mutants would require the generation of multiple input datasets (Fig 1, top inset; Song et al., 2012). To improve this aspect of the model, we wished to incorporate circadian regulation of CDF1 and FKF1 mRNA, removing data inputs to the model (Fig 1, bottom inset). This allowed all components to be simulated in any given condition, and therefore allowed us to investigate how changes in clock dynamics (e.g. in clock mutants or different photoperiods) affect components of the flowering pathway. By postulating and experimentally validating circadian regulators of CDF1 and FKF1 mRNA, the model recapitulates the acceleration of flowering in LDs.

In the hypocotyl elongation pathway, we demonstrate that known transcriptional and post-transcriptional regulation of PIF explains phenotypes and PIF target transcript dynamics under a

© European Molecular Biology Organization

variety of environmental and genetic manipulations. We then use microarray data to identify other transcripts that have similar dynamics, and that are therefore likely to be under the control of PIFs in light:dark cycles. Finally, we explore crosstalk between the flowering and hypocotyl pathways by simulating PIF regulation of FT mRNA, in order to test the hypothesis that temperature regulates flowering independently of CO. The results highlight the complexity of the network structure underlying circadian-, light- and temperature-regulated processes."

There needs to be some kind of transition from the CDF1/FKF1 models to the PIF4/5 models, as as it reads now, it is a leap for the reader and there is no connection in the results. I like the first sentence in the PIF4/5 section, but I think something above in the CDF1/FKF1 section is needed as a bridge to finalise the connections.

We have added modified the concluding sentences to the CDF1/FKF1 section as follows (page 9):

"Thus the present model is consistent with past results as well as additional molecular and genetic data, providing a suitable basis for further extension. In particular, we extended the model to consider the combined circadian and light regulation of PIF4 and PIF5 activity, allowing us to investigate the regulation of rhythmic growth by PIF4 and PIF5, and to understand crosstalk between PIF4, PIF5, and CO in the regulation of FT."

For "The model including this regulation captures..." I recommend an expect statement of what model was made. More information of the intentions are needed to engage in this thought line. Maybe something like "Here we will describe the methods that led to the development of a model that described PFI4/5 expression and connected that to the physiology of photoperiodic induction of growth."

We have modified this section so that the motivations and objectives of this modelling are clear (page 9):

"Hypocotyl elongation, like flowering time, is subject to photoperiodic regulation. In contrast to the FKF1-CO-FT pathway, which is active in LDs, PIF4,5-induced hypocotyl extension is observed in SDs (Niwa et al., 2009; Kunihiro et al., 2011). Here, we describe the development of a model describing the photoperiodic induction of hypocotyl elongation through PIF4 and PIF5. As a first step, we constructed a model of PIF4 and PIF5 transcription, which is known to be controlled by the circadian clock (Yamashino et al., 2003). This regulation has been shown to involve direct inhibition of transcription by the EC (Fig 4A; Nozue et al., 2007; Nusinow et al., 2011). In order to test whether this regulation is sufficient to explain observed patterns of PIF4 and PIF5 expression, we started by constructing a model in which the EC is the sole regulator of PIF4 and PIF5 transcription."

For "However, CCA1 ChIP experiments did not provide strong evidence to support this hypothesis." please be explicit that CCA1 binding to PIF4/5 does not match the hypothesis. As written the reader cannot understand why there is no strong evidence.

This sentence now reads (page 9):

"However, our CCA1 ChIP experiments did not detect binding of CCA1 to the PIF4 and PIF5 promoters (Supplementary Fig 3)."

The paragraph "The generality of this model of PIF target regulation was further tested ..." starts well, but lacks an explanation of the findings. Then it lacks a brief conclusion sentence describing the implications of the testing of the model.

This section now concludes (page 11):

"The consistency of model simulations with experimental data for the identified PIF-induced transcripts under diverse perturbations suggests that PIF4 and PIF5 are the dominant regulators of these transcripts in light:dark cycles."

I cannot find grant BB/F59011/1 at the BBSRC website. Is this code correctly typed?

This is not listed on the website as it was a graduate training grant (now specified as such).

Errors exist in the references, such as:

"Bai MY, Fan M, Oh E, Wang ZY (2012b) A triple helix-loop-helix/basic helix-loop-helix cascade controls cell elongation downstream of multiple hormonal and environmental signaling pathways in Arabidopsis. Plant Cell doi: 10.1105/tpc.112.105163" has a volume and page as 24(12):4917-29

Similarly fix, "Antagonistic basic helix-loop- helix/bZIP transcription factors form transcriptional modules that integrate light and reactive oxygen species signaling in Arabidopsis."

"Doyle MR, Davis SJ, Bastow RM, McWatters HG, Kozma-Bognar L, Nagy F, Millar AJ, Amasino RM (2002) The ELF4 gene controls circadian rhythms and flowering time in Arabidopsis thaliana. 419: 74-77." journal missing

Font problems in "Thines BC, Youn Y, Duarte MI, Harmon FG (2014) The time of day effects of warm temperature on flowering time involve PIF4 and PIF5. J Exp Bot. 65(4):1141-51"

We thank the reviewer for pointing out these errors, and have duly corrected them.

For figure 2 and 3, supp 1, supp2 supp7, supp8, supp15 I strongly recommend the light blue and light green lines are put to stronger, easier to see colours. The light blue in particular is almost impossible to see and will poorly print.

We have changed these colours in the above referenced figures, and increased the line thickness in many figures to improve visibility.

Reviewer #4:

This paper represents an important advance in mathematical models of circadian phenomenon in plants. In previous work models had been developed to describe the behavior of the circadian oscillator itself. Separate models had been developed for control of flowering time (an important circadian output). However previous flowering time models did not incorporate the oscillator itself; they instead relied on measured oscillatory behaviors of the flowering time genes. This limited the ability of those models to be predictive for genetic or environmental perturbations of the clock.

The work here develops a new model for flowering time that overcomes the previous limitations by directly incorporating a model of the central oscillator and linking it to the relevant flowering time genes. This provide a much more complete and predictive model for flowering time. In addition, the authors develop a model for control of hypocotyl elongation that incorporates both the oscillator and key players that control rhythmic growth behaviors--the first of its kind to my knowledge.

The paper is well written, although somewhat dense (I don't think much can be done about that--there is a lot of information here). Conclusions are justified, explanations are clear. I have only minor comments that need to be addressed.

We thank the reviewer for their comments and for their detailed reading of the manuscript.

Minor comments:

p. 4. The first report that PIFs were degraded in the light came from Park (2004). Although Park studied PIF3 rather than PIF4 and 5 described here, the paper should still be cited.

© European Molecular Biology Organization

This is now referenced (page 4):

"During the day, PIF activity is thought to be compromised due to interactions with phytochrome B (phyB), the key red light photoreceptor, resulting in rapid PIF phosphorylation and degradation (Park et al., 2004; Al-Sady et al., 2006; Nozue et al., 2007; Jang et al., 2010; Park et al., 2012)."

p. 8. "with mutations to clock genes". I think this should read "with mutations IN clock genes".

We have modified this text as suggested.

p. 8. Not only does the model fail to capture the dynamics of CO and FT in the cca1;lhy1 double (discussed), it also fails to capture CO dynamics in the elf3 mutant (Sup Fig 7G--not discussed). This should be noted/briefly discussed.

This is now mentioned (page 8):

"In the case of the elf3 mutant, model simulations matched the increase in FT expression in both SDs and LDs despite overestimating the increase in CO mRNA during the day (Lu et al., 2012; Supplementary Fig 7; data not used for parameter optimisation)."

Compared to the case of *cca1;lhy*, there is less complete data for *elf3*. For example, it's not known how *FKF1* and *CDF1* are changed in the *elf3* mutant, so it's difficult to identify the root cause for the quantitative misprediction of *CO* mRNA dynamics. As a result, we don't make a prediction about alternative roles of *ELF3*.

Figure 4 Legend. SD and LD are swapped in the legend for 4B-G.

We have corrected this error.

p. 9. The authors state: "In an elf3 background, the level of PIF4 transcripts is increased throughout the night...this behavior is matched by simulations (Sup 9A,D). This does not do justice to the differences between the data and the model. In the observed data there is an increase throughout the night, whereas the model has it constant. This is somewhat acknowledged a few sentences later "Residual rhythms..." but I think this section should be reworked. The same criticism is true of simulated ELF3 in the prr9;7;5 mutant.

We have highlighted this difference, and noted that the difference is consistent across both the elf3 and prr975 mutants (page 9):

"Additionally, it should be noted that model simulations do not reproduce the steady increase in PIF4 expression observed during the night in both the elf3 and prr9;7;5 mutants (Supplementary Figs 9A,B & 10A,B). Rather, model simulations in both cases predict a constant high level of PIF4 transcript."

p. 9. "...a small daytime peak in expression of PIF4 and PIF5...". Please clarify: do you mean in wild type or in elf3 mutants? I assume the latter but is not clear.

We have clarified that this refers specifically to the *elf3* mutant (page 9):

"In particular, a small daytime peak in expression of PIF4 and PIF5 in the elf3 mutant has been observed in multiple experiments (Lu et al., 2012; Nomoto et al., 2012b)..."

p. 11. In the middle paragraph ("The generality..."). the callouts to Sup. Fig. 9 and 10 are backwards.

We have corrected this error.

p. 11, same paragraph as previous comment. Interpretation of the results described in this

paragraph should be given.

A concluding sentence has been added to this paragraph (see similar comment from reviewer #3,above).

p. 13, first paragraph. It is great that the patterns are qualitatively the same. Please also discuss the quantitative differences that confirm an important role for light/phyB regulation of PIF targets.

We now discuss this point, which provides insight into how the role of phyB may differ between constant light and light:dark cycle conditions (page 13):

"Interestingly, a significant increase in PIF-induced transcript levels is observed during the night in the phyB mutant (Supplementary Fig 16A). This may explain the long hypocotyl phenotype of this mutant in light:dark cycles despite phyB's apparent redundancy in repressing PIF activity during the day, and suggests that phyB regulates PIF activity during the night through a separate mechanism."

p. 13-14. Figure 6 is called out before Figure 5.

We have amended the ordering of the figures.

p. 14-15. This section seems to be organized oddly. That is the fit of the model is described in detail first, and then the details of how the model was constructed (i.e. FLM and SVP) are discussed in a separate section. Consider reworking this.

The model of temperature regulation was constructed by first modelling the behaviour at 22°C, as described in the paper up to this point. The additional inclusion of FLM/SVP/additional temperature regulation does not affect the behaviour of the model at 22°C, meaning that the extension of the model to 27°C can be treated as a separate case. This is now clarified in the text (page 16):

"The action of these regulators can be modelled by introducing a uniform activation of FT expression at 27°C, leaving the model behaviour at 22°C unchanged."

Other changes:

In addition to the changes detailed above in response to reviewers, we have also modified the text to include references to two relevant recent publications: Bernardo-García et al., 2014 provide independent confirmation of significant daytime levels of PIF4 protein, in agreement with Yamashino et al., 2013. Berns et al., 2014 performed a detailed analysis of diurnal regulatory elements in the GI and FKF1 promoters, providing further evidence that they are similarly regulated (as we argued based on literature data).

© European Molecular Biology Organization
